# Proteomics Unravels Emodin Causes Liver Oxidative Damage Elicited by Mitochondrial Dysfunction

**DOI:** 10.3389/fphar.2020.00416

**Published:** 2020-04-29

**Authors:** Yinhuan Zhang, Xiaowei Yang, Zhixin Jia, Jie Liu, Xiaoning Yan, Yihang Dai, Hongbin Xiao

**Affiliations:** ^1^Research Center of Chinese Medicine Analysis and Transformation, Beijing University of Chinese Medicine, Beijing, China; ^2^Institute of Chinese Materia Medica, China Academy of Chinese Medical Sciences, Beijing, China

**Keywords:** Emodin, liver injury, proteomics, mitochondrial dysfunction, redox homeostasis

## Abstract

Emodin is one of the main active compounds in many Chinese traditional herbs. Due to its potential toxic effect on the liver, the possible injury mechanism needs to be explored. In the present study, we investigated liver injury mechanisms of emodin on rats by the technology of proteomics. Firstly, 4530 proteins were identified from the liver of rats treated with emodin by label free proteomics. Inside, 892 differential proteins were selected, presenting a downward trend. Bioinformatics analysis showed that proteins interfered with by emodin were mainly involved in oxidation-reduction biological processes and mitochondrial metabolic pathways, such as mitochondrial fatty acid β-oxidation, citric acid cycle, and oxidative phosphorylation, which were further confirmed by western blot. The decrease in maximal respiration, ATP production, spare respiratory capacity, and coupling efficiency and increase in proton leakage were detected by seahorse XFe 24 analyzer, which confirmed the damage of mitochondrial function. The down-regulated expressions in antioxidant proteins were verified by western blot and a significant increase of ROS levels were detected in emodin group, which showed that emodin disrupted redox homeostasis in livers. Molecular docking revealed that the main targets of emodin might be acadvl and complex IV. Generally, emodin could induce oxidative stress in livers by directly targeting acadvl/complex IV and inhibiting fatty acid β-oxidation, citric acid cycle, and oxidative phosphorylation taken place in mitochondria.

## Introduction

Due to its wide and unpredictable effects, drug-induced liver injury (DILI) has become one of the main obstacles in drug safety development and clinical application. Hence, a better understanding of key signaling mechanisms driving initiation and progression of this untoward reaction may contribute to both early diagnosis and safe use of drugs. Critical events in the development of DILI are mitochondrial damage and oxidative stress (Lim et al., 2006; Berson et al., 2006). Mitochondria are important organelles; on one hand, they are the sources of power which are responsible for the production of the majority of adenosine triphosphate (ATP) through oxidative metabolism by tricarboxylic acid (TCA) cycle, fatty acid β-oxidation and oxidative phosphorylation (OXPHOS). On the other hand, they are the main source of reactive oxygen species (ROS) in mammalian organs. Oxygen accepts a single electron derived from FMN**.** of the FMNH2/FMN coenzyme of mitochondrial respiratory chain complex I (Turrens and Boveris, 1980) and double electron from UQH**.** of the complex III (Boveris et al., 1976) to be reduced to form superoxide radicals and hydrogen peroxide, which constitute a constant source of the largest amount of ROS in the organism. Mitochondrial damage gives rise to the increase of ROS (Wang et al., 2013) such as the lack of any component of the TCA cycle or electron transport chain. Interestingly, to prevent oxidative damage, there is a set of scavenging systems including enzymatic and non-enzymatic antioxidants to protect cells from the attack by ROS (Valko et al., 2007). The imbalance between ROS and antioxidant defenses result in oxidative stress (Myatt and Cui, 2004; Burton and Jauniaux, 2011). Oxidative stress leads to the damage of important biomolecules and cells, which has a potential impact on the whole organism (Ďuračková, 2010). Moreover, oxidation stress can further damage the mitochondrial metabolic process (Duchen, 2004) and lead to liver injury.

Emodin (1, 3, 8-trihydroxy-6-methylanthraquinone) is a natural active compound isolated from *Rheum palmatum* (Wang et al., 2011), *Polygonum cuspidatum* (Wang et al., 2012), *Polygonum multifloyum* (Lee et al., 2011), *Aloe vera* (Naqvi et al., 2010), and *Cassia obtusifolia* (Yang et al., 2003) etc. As one of their mutual active ingredients, emodin is considered to be responsible for the toxic effect on livers (Yang et al., 2019). Previous studies have shown that emodin can elevate ROS levels accompanied by consumption of SOD and GSH (Ma et al., 2015; Jiang et al., 2017; Jiang et al., 2018) and lead to oxidative stress (Cui et al., 2014). Further studies have found that emodin can alter mitochondrial membrane potential (Cui et al., 2014), down-regulate GAPDH expression and MDH activities (Yang et al., 2018), and inhibit protein expressions and activities of mitochondrial respiratory chain (Lin et al., 2019), which implied that emodin has a potential damaging effect on liver mitochondrial function. Moreover, emodin can also induce DILI in the way of hepatocyte apoptosis *via* mitochondria-dependent pathways including up-regulating apoptosis protein expressions of cyt c, caspase 3, and caspase 9 (Yang et al., 2018). In addition, oxidative stress has been shown to destroy mitochondria metabolic processes (ie, Fatty acid oxidation, ATP production) (Galluzzi et al., 2012). In hepatocytes, the inhibition of mitochondrial function induces oxidative stress, which decreases fatty acid oxidation (Pettinelli et al., 2011; Bechmann et al., 2012). Our previous study had reported that emodin interfered with three fatty acid β-oxidation metabolites including N-undecanoyl glycine, L-palmitoyl carnitine, and eradi carnitine based on metabonomics (Liu et al., 2015a). Yet it is unclear how emodin induces oxidative stress by affecting mitochondrial metabolism function.

In recent years, mass spectrometry-based proteomics has been widely used to determine the modes of action and mechanisms involved in drug- or chemical-induced toxicity (Tan et al., 2012; Lee et al., 2013). A label-free approach generally quantifies based on peak intensity, and it has no sample limitation, so this approach has the ability to accurately quantitate more samples. Besides, there are few restrictions in terms of experimental conditions, so almost any type of sample can be used. Despite the popularity of proteomics, the information on emodin-induced hepatotoxicity is still limited which challenges toxicity monitoring and evaluation.

Therefore, in this study, we try to explore the effect of emodin on oxidative stress and mitochondrial function in rat livers using proteomic technology. This work is expected to sharpen understanding of the liver injury mechanism induced by emodin and provide reference for the further development and application of drugs containing emodin.

## Materials and Methods

### Drugs and Reagents

Emodin (1, 3, 8-trihydrow-6-meth-ylanthraquinone) was purchased from Chengdu Ruifen Si Biological Technology Co., Ltd. (Chengdu, China, purity≥98%). Electrophoresis agents [sodium lauryl sulfate (SDS), acrylamide, N, N'-Methylenebisacrylamide (Bis), Tris (hydroxymethyl) amino methane (Tris), Glycine (Gly), ammonium persulfate (Aps), N, N, N', N'-Tetramethylethylenediamine (TEMED), Bromophenol Blue (BPB), glycerol, urea, and mercaptoethanol] were obtained from Beijing Bio Dee Biotechnology Co.Ltd. (Beijing China). Hcl was purchased from Beijing Chemical Works (Beijing, China). A protease inhibitor cocktail was obtained from Roche (Mannheim, Germany). Coomassie Blue R250 was purchased from Sigma (USA).All other chemicals were of analytical grade reagent. Deionized water (R > 18.2 MΩ) used for all experiments was purified by using Millipore purification system (Billerica, MA, USA). Chloraldehyde hydrate (S24149) was purchased from Beijing Honghu United Chemical Products Co., Ltd. Skimmed milk powder (Q/NYLB 0039 S) was purchased from Yili Company. NaCl (PBZ0637-3) was purchased from Beijing Oubei Biotechnology Co., Ltd. Tween 20 and dithiothreitol were purchased from amresco, USA; iodoacetamide and ammonium bicarbonate were purchased from Beijing Inoke Technology Co., Ltd. Bradford Assay kits, ECL kits were purchased from Biyuntian Biotechnology Co., Ltd. Color prestained protein Marker was purchased from Abfans. PVDF membrane (Immobilon-P; Millipore Corp) was purchased from Bedford, MA, USA. Seahorse XF cell mitochondrial stress test kit (oligomycin 2.5 mmol/L, carbonyl cyanide 4-(trifluoromethoxy) phenylhydrazone (FCCP) 2.5 mmol/L, Antimycin 2.5 mmol/L, Rotenone 2.5 mmol/L) were purchased from Agilent, USA. Anti-Ndufs3, Anti-Txn2, Anti-Prdx3, Anti-Gpx3, and Anti-Glrx5 were obtained from Bioss Co., Ltd. Anti-Atp5g1, Anti-Acads, Anti-Hadhb, Anti-Sdhb, Anti-Uqcrh, Anti-Cox5a, Anti-CS, and Anti-Idh3a were obtained from Abcam Co., Ltd (England).

### Animal Treatments

Male Sprague-Dawley rats (9 weeks old, 200-300 g) were purchased from Beijing Vital River Laboratory Animal Technology Co., Ltd. (Beijing, China). Rats were housed at a constant room temperature and humidity (21 ± 2°C; 45−55%) under a 12−h light/dark cycle. The rats had ad libitum access to food and water. All animal experiments were approved by the Committee on Animal Care and Use of Institute of Chinese Materia Medica, China Academy of Chinese Medical Sciences, and the protocol was approved by Animal Ethical and Welfare Committee of CACMS.

Twelve rats were randomly assigned to either a control group (6 rats) or an emodin group (6 rats). Rats in the emodin group were orally administrated with emodin (150 mg/kg daily, 10 times the clinical equivalent of rhubarb), and rats in the control group received the same volume of physiological saline. After four weeks administration, all rats were sacrificed. Blood was collected and centrifuged to get serum for pathological examination. After blood collection, livers of rats in each group were quickly removed by laparotomy and divided into several small pieces, some of which were fixed in 4% polyformaldehyde solution for histopathology, while the rest of the liver tissue samples were then immediately frozen by immersion in liquid nitrogen, and stored at -80°C for proteomic analysis.

### Extraction and Gel Separation of Rat Liver Proteins

Equal amounts of liver samples from all animals in each group were pooled and prepared. Three biological replicates were prepared for the control group and emodin group, which were used for proteomics study. The detailed steps of protein extraction are as follows: Rat livers were homogenized in the PBS buffer containing protease inhibitor cocktail with a high-throughput tissue homogenizer (Sceintz-48, Ningbo, China). Then, proteins of rat livers were extracted with 8 M urea and total protein concentration was detected with the Bradford Assay kit (P0006, Beyotime Institute of Biotechnology, China). After quantiﬁcation, equal amounts of proteins (30 µg) were separated with 10 or 12% sodium dodecyl sulfate polyacrylamide gel electrophoresis (SDS-PAGE). The gels ran on the Bio-Rad electrophoresis system at 80 W/gel for 20 min and then ran at 120 W/gel until bromophenol blue reaches the bottom of the gel. The gels were stained using coomassie brilliant blue.

### Gel Tryptic Digestion

Protein bands of interest were excised from the preparative gels, and reduced with 25 mmol/L dithiotreitol, then alkylated in 55 mmol/L iodoacetamide and carried out overnight at 37°C with sequencing grade modified trypsin in 50 mmol/L ammonium bicarbonate. The peptides were extracted twice with 0.1% trifluoroacetic acid in 50% acetonitrile aqueous solution for 30 min. The extractions were centrifuged to reduce the volume. The digested peptides were dissolved into 0.1% formic acid. Equal amounts of proteins from rat livers were combined and analyzed by LC-MS/MS.

### Label-Free Quantitative Proteomics

The identification of trypsin-digested proteins was performed through a Thermo-Dionex Ultimate 3000 HPLC coupled to a Thermo LTQ-Orbitrap Velos Pro mass spectrometer. Peptides were separated on an analytical homemade fused silica capillary column (75 μm ID, 150 mm length; Upchurch, Oak Harbor, WA) which was packed with C-18 resin (300 A, 5 μm; Varian, Lexington, MA) at a flow rate 0.250 μL/min. Mobile phase A was 0.1% formic acid aqueous solution, and mobile phase B was 100% acetonitrile containing 0.1% formic acid. Data acquisition was performed using Xcalibur 2.0.7 software. Mass spectrometry acquisition was in positive ion mode. Detailed parameters are as follows: capillary voltage8 V, cone voltage 100 V, ion source voltage 2.4 kV, Data acquisition range m/z 400-1800, Scanning in centroid mode with 60000 resolution, Linear ion trap dynamic exclusion scanning mode is adopted in the detection of secondary mass spectrometry Orbitra, first level full scan with 10 strongest LTQ secondary scans, signals with the same m/z appeared within 20 s were dynamically eliminated using secondary scan. The secondary scan chose the CID mode, the collision gas is high-purity helium (99.99% He), collision standardized energy is controlled at 35%.

Proteins were identified by their peptide mass fingerprint data search against the Rattus norvegicus database with Proteome Discoverer (Version 1.4). The criteria of identification were set as the following: full tryptic specificity was required; two missed cleavages were allowed; carbamidomethylation was set as fixed modification; oxidation (M) were set as variable modifications; precursor ion mass tolerance was 10 ppm for all MS acquired in the Orbitrap mass analyzer; and fragment ion mass tolerance was 0.8 Da for all MS2 spectra acquired in the LTQ. High confidence score filter (FDR < 1%) was used to select the “hit” peptides. Finally, the proteins were identified and analyzed artificially to confirm the authenticity.

### Bioinformatics Analysis of Differentially Expressed Proteins

GO enrichment analysis with biological process and molecular function of potential targets were carried out for biological function annotation based on a DAVID bioinformatics database (https://david.ncifcrf.gov/gene2gene.jsp). KEGG Pathway database (http://www.genome.jp/kegg/pathway.html) was utilized to analyze the representative pathways of the differentially expressed proteins and a p-value ≤ 0.05 significant level was used. Protein interaction analysis was performed by string database (https://string-db.org/). The visualization of graphs was made by Cytoscape (version 3.6.0).

### Western Blot Analysis

Equal amounts of protein (30 µg) from each sample were separated on SDS-PAGE and electrophoretically transferred onto a PVDF membrane (Immobilon-P; Millipore Corp., Bedford, MA, USA) and blocked in 10% nonfat powdered milk in Tris-Buffered Saline Tween-20 (TBST: 10 mmol/L Tris-HCl; 150 mmol/L NaCl; 0.05% tween-20; pH 7.6) overnight at 4°C. The membranes were incubated in primary antibodies overnight at 4°C or at 37°C for 2 h. After washing three times in TBST, HRP-labeled Goat Anti-Mouse IgG antibody or HRP-labeled Goat Anti-Rabbit IgG antibody was added to membranes and incubated for 2 h at room temperature. The membranes were then washed three times in TBST for 15 min, followed by signal detection using an ECL detection kit from Millipore Corporation. β-actin was utilized as a housekeeping protein.

### Mitochondrial Stress Test Assay

Mitochondrial function was assessed using the Seahorse XFe 24 Analyzer (Agilent Technologies, Santa Clara, CA). 5, 000 L-02 cells per well were seeded on Cell-Tak coated Seahorse plates. L-02 cells were treated with 10.93 μmol/L of emodin for 24 h (Zhang et al., 2019) prior to assessing mitochondrial function. Cells were equilibrated in XF media before testing the mitochondrial oxygen consumption rate (OCR). The mitochondrial stress test was carried out *via* the manual steps described. Using the mitochondrial stress test inhibitors, oligomycin (0.5μmol/L), FCCP (0.5mol/L), and rotenone and antimycin A (10 μmol/L) were used to gain basal respiration, maximal respiration, ATP production, proton leak, spare respiratory capacity, and coupling efficiency.

### Measurement of Reactive Oxygen Species

Take 0.5 g liver tissue and homogenate on ice for 10 minutes with 0.09% normal saline using a high-throughput tissue homogenizer (Sceintz-48, Ningbo, China), and then centrifuge at 10000g for 10min at 4°C, collect the supernatant, then detect the level of ROS by Luminol chemiluminescence method.

### Molecular Docking

Three-dimensional structures of the target proteins were downloaded from Protein Data Bank database (https://www.rcsb.org/pdb/home/home.do). Firstly, emodin was imported into the Discovery Studio 3.5 software for hydrogenation optimization. Next, the three-dimensional structure of target proteins was introduced and optimized through dehydration, hydrogenation, and structural modification. Emodin was docked with target proteins respectively. The higher the total score, the more stable the binding between the ligand and the receptor. If the total score is ≥ 6, it can be inferred that there is a strong interaction between the component molecule and the corresponding protein target. Moreover, the closer the absolute value of crash is to 0, the more likely the target protein can be considered.

### Statistical Analysis

All experimental data obtained from rats were expressed as mean ± standard deviation (SD). The signiﬁcance of the differences among different treatment groups was determined using Student's t-test by GraphPad Prism 6.0 software. The level P < 0.05 was considered to indicate a statistically significant difference.

## Results

### Comparative Proteomic Analysis in Livers Between Emodin Group and Control Group

**Figure 1:**
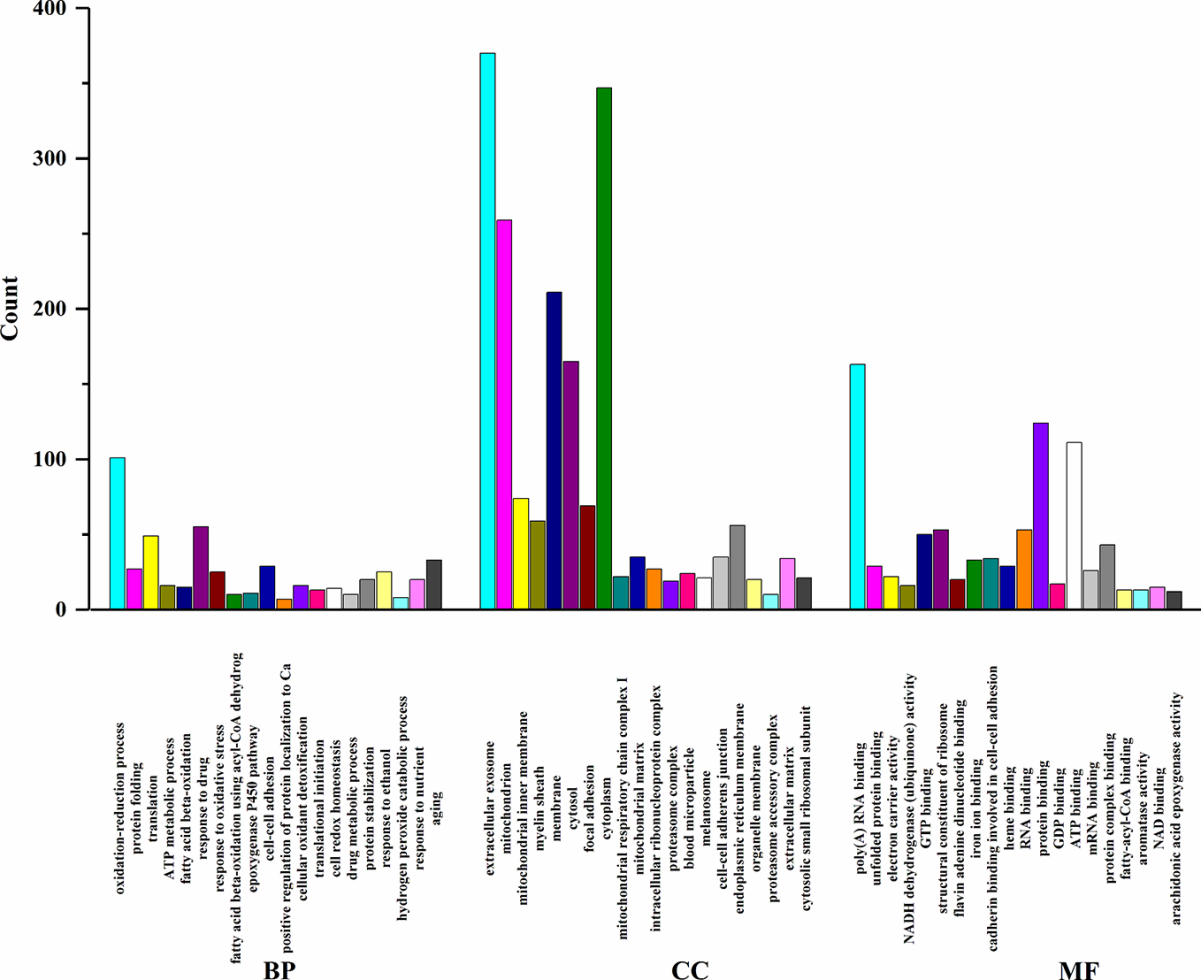
GO enrichment analysis of differentially expressed proteins.

**Figure 2:**
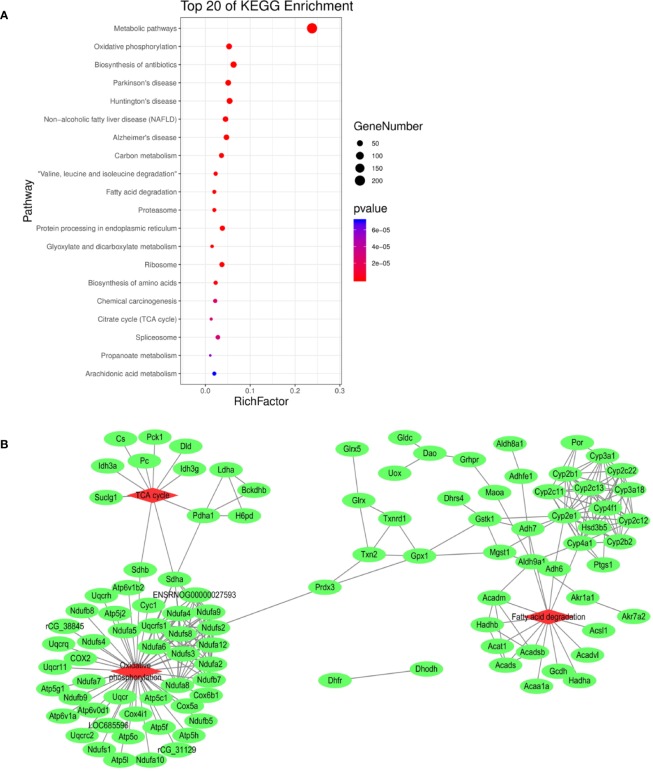
**(A)** KEGG pathway analysis of differentially expressed proteins. **(B)** Interaction analysis of proteins involved in oxidation-reduction biological process. The green ellipse represents the proteins, and the red diamond represents the pathways.

In order to elucidate the effects of emodin on liver function, we measured the activities of serum alanine aminotransfease (ALT) and aspartate transaminase (AST) in different groups before (Yang et al., 2018), results showed that in emodin group, increased ALT and AST levels were detected in rats and these changes were significant (P < 0.05). Histopathology observation showed that HE-stained hepatic sections in the control group were microscopically normal. After being treated with emoin (150 mg/kg daily) for four weeks, mild inflammation changes were found in the liver (Yang et al., 2018). Further, we explored the liver injury mechanism induced by emodin. To characterize the expressions of proteins in response to emodin exposure, the label-free quantitative proteomics technique was used. Firstly, equal amounts of the control group and emodin-treated group were initially separated by SDS-PAGE, then differentially expressed proteins were qualitatively and quantitatively tested by unlabeled methods. The experiment was repeated three times, and each sample identified approximately 4,530 proteins (≥1 peptide). According to P < 0.05 and F > 1.2 or <0.67, 892 differentially expressed proteins were screened and their expressions were all down-regulated under emodin administration. The 892 differentially expressed proteins data can be seen in **Table S1**. In order to analyze the biological significance of differential proteins, the David Bioinformatics Platform was used. The top 20 significantly enriched terms in Biological Process, Molecular Function, and Cellular Component categories were selected, according to P < 0.05, P-values were corrected using the Benjamini-Hochberg procedure, as shown in **Figure 1**. In the biological process category, proteins were mainly involved in the oxidation-reduction biological process, ATP metabolic process, or fatty acid β-oxidation. Based on cell composition, proteins were mainly attributed to extracellular exosome, mitochondria, and mitochondrial inner membrane. In the molecular function category, proteins were mainly involved in poly RNA binding, unfolded protein binding, and electron carrier activity (**Figure 1**). In addition, KEGG database was used to enrich differentially expressed proteins, according to BH-corrected P-values < 0.05, the top 20 pathways were screened. Results demonstrated that the diﬀerentially expressed proteins mainly participated in metabolic pathways, OXPHOS, and Biosynthesis of antibiotics (**Figure 2A**).

Redox reaction is the most basic motive force and the most common metabolic reaction mechanism of biological evolution and life activities. To explore the relationship between mitochondrial metabolism and redox disequilibrium under the influence of emodin, we mainly analyzed 101 proteins in oxidation-reduction biological process. The 101 proteins data can be seen in **Table S2**. The proteins were submitted to string database for PPI network construction. High confidence of protein interaction data with a score of more than 0.7 were selected and hide disconnected nodes in the network. The results visualized by cytoscape (**Figure 2B**) showed that fatty acid degradation, TCA. and OXPHOS were involved. Taken together, by analyzing the proteomics data, it preliminarily speculated that emodin may mainly interfere with fatty acid β-oxidation, TCA, and OXPHOS to destroy redox homeostasis and cause hepatic injury.

### Emodin Led to Mitochondrial Dysfunction

**Figure 3:**
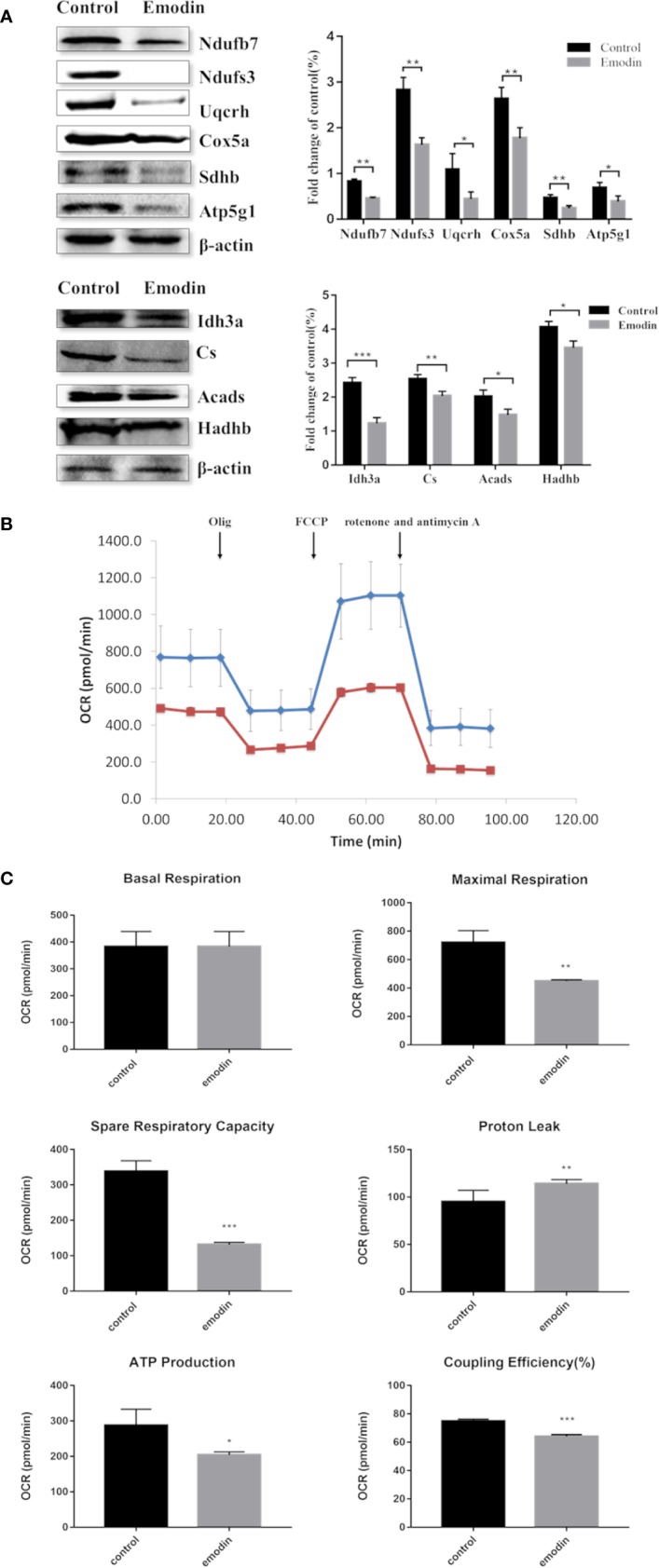
**(A)** KEGG pathway analysis of differentially expressed proteins. **(B)** Interaction analysis of proteins involved in oxidation-reduction biological process. The green ellipse represents the proteins, and the red diamond represents the pathways.

Among the 892 differential proteins, we specifically found the key proteins involved in fatty acid β-oxidation (**Table S3**), mitochondrial respiratory chain (**Table S4**), and TCA cycle (**Table S5**) in proteomic data, whose protein expressions were down-regulated by emodin. Thus, we next tested if emodin can alter the metabolic activity of mitochondria. According to the functional identification and fold-change-ratio changes, Acads, Hadhb, Ndufs3, Ndufb7, Sdhb, Uqcrh, Cox5a, Atp5g1, Cs, and Idh3a were selected for western blot analysis. Results (**Figure 3A**) demonstrated that emodin inhibited protein expressions of Acads, Hadhb, Ndufs3, Ndufb7, Sdhb, Uqcrh, Cox5a, Atp5g1, Cs, and Idh3a in various degrees, which indicated that emodin might inhibit the function of fatty acid β-oxidation, TCA cycle, and OXPHOS taken place in mitochondria.

Next, a mitochondrial stress test was performed by sequential use of oligomycin, FCCP, rotenone, and antimycin A. The overall OCR profile was lower in the emodin group compared to the control group (**Figure 3B**). Between them, maximal respiration, ATP production, spare respiratory capacity, and coupling efficiency showed progressive decline with emodin treatment (**Figure 3C**). Only proton leak increased in the emodin group (**Figure 3C**) which can be increased by oxidative stress. However, basal respiration was not affected (**Figure 3C**). These results indicated that emodin disturbed the function of mitochondria by inhibiting the expressions of proteins related to fatty acid β oxidation, citric acid cycle, and mitochondrial respiratory chain.

### Emodin Destroyed Redox Equilibrium

**Figure 4:**
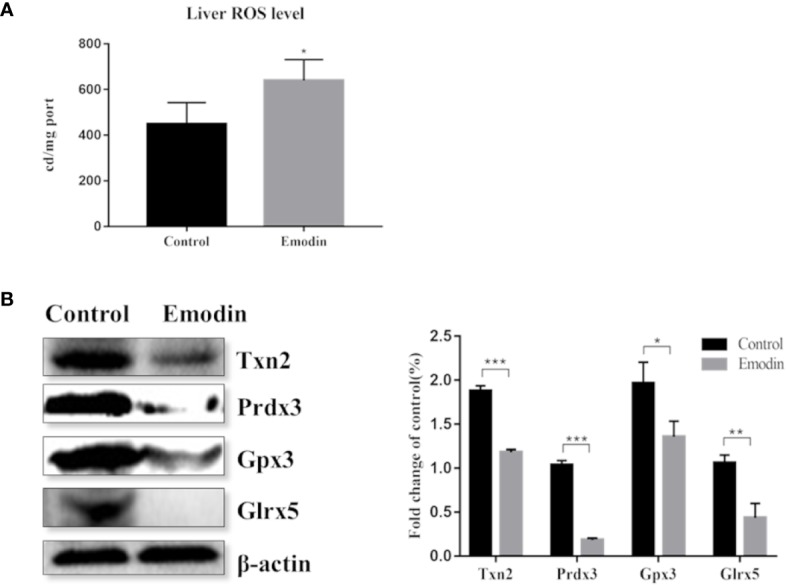
**(A)** Change of the ROS level between the emodin group and the control group. **(B)** Western blot analysis of differentially expressed antioxidant proteins, including protein bands and relative abundance. Values are mean ± SD, n = 3. * for P value <0.05, ** for P value <0.01, *** for P value <0.001.

Proteomic data showed that emodin mainly affected the redox biological process. Therefore, we investigated whether emodin can induce the generation of oxidative stress in livers. By detecting ROS with reference to protocol of ROS test kit (E004, Nanjing Jiancheng Bioengineering Research Institute), result showed that emodin led to a significant increase in ROS levels (**Figure 4A**).

Inhibition of the antioxidant defense system contributes to oxidative stress. By digging up the proteomic data, 11 antioxidant proteins were down-regulated (**Table S6**) in emodin group, including glutathione reductase, thioredoxin, glutathione peroxidase, and peroxide reductase protein. Based on western blot, proteins with high protein fold-change-ratio were chosen to be verified. The results (**Figure 4B**) showed that their protein expressions in the emodin group were significantly lower than those in the control group. Results showed that emodin inhibited antioxidant function in livers and induced oxidative stress.

### Exploration of Emodin Targets Through Molecular Docking

**Figure 5:**
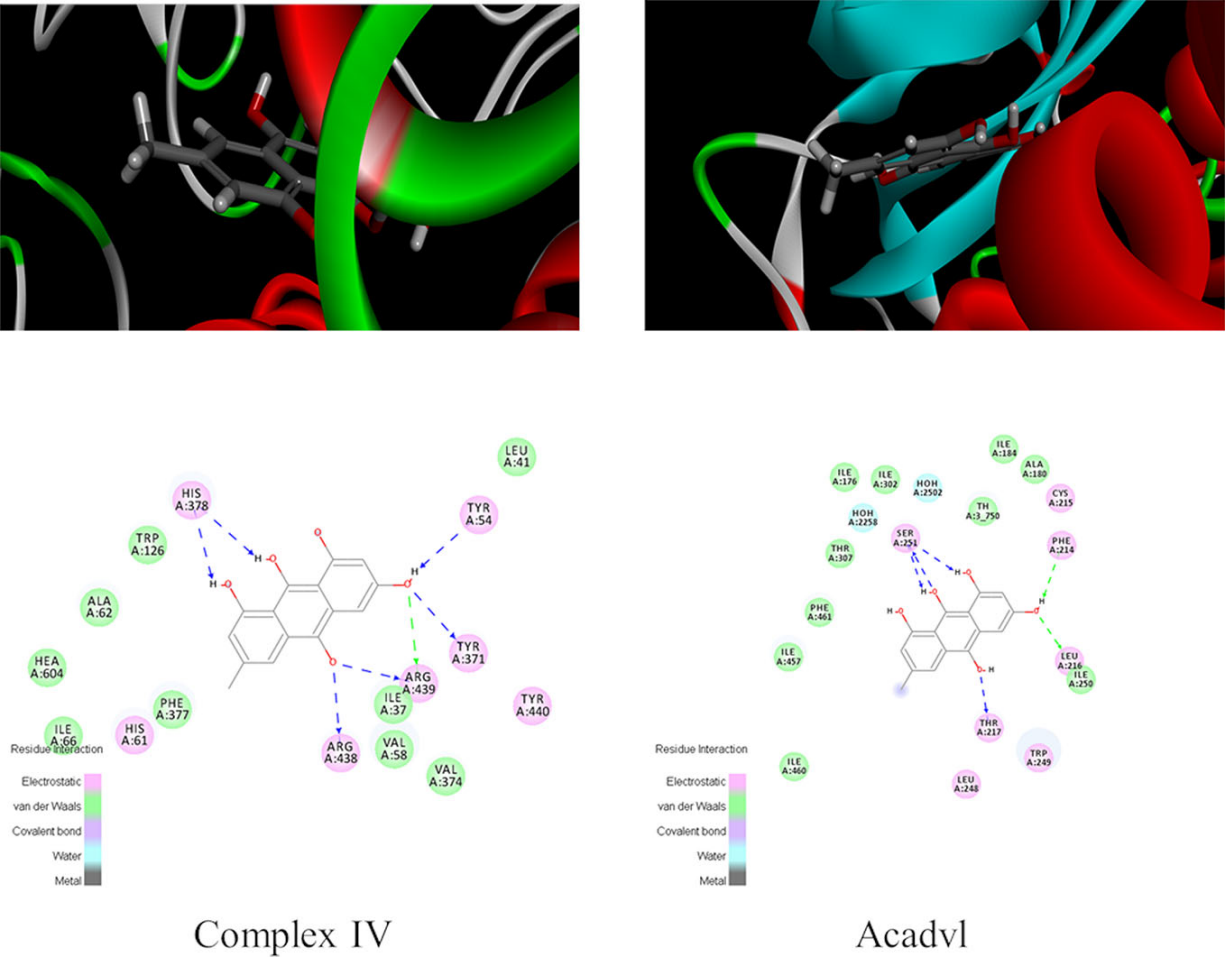
Graphs of molecular docking results.

Molecular docking experiment was conducted to further explore the liver injury mechanism of emodin and to clarify the targets of emodin in affecting redox balance *via* mitochondrial metabolism. Firstly, fatty acid oxidation, TCA cycle, OXPHOS, and antioxidant related proteins in proteomic data were found in PDB database. Secondly, human proteins with ligands were selected. Proteins with emodin/ligand value close to 1 were selected to remove the negative results. Then, according to the total score, protein with high scores were selected. Generally, a total score greater than 6 was considered. The scores of Acadvl and complex IV were 7.6312 and 8.0158 respectively, hence, Acadvl and complex IV were selected (**Table S7**). The molecular docking results of emodin and ligands were shown in **Figure 5**. Emodin could form hydrogen bonds with His, Tyr, and Arg residues in Complex IV, and it also had hydrogen bonding forces with Ser, Phe, Leu, and Thr of Acadvl. The results showed that there were interactions between emodin and residues of Acadvl and complex IV. Hence, Acadvl and complex IV may be the possible targets of emodin in liver injury signal pathway.

## Discussion

Recent evidence suggested that oxidative stress and mitochondrial pathways cooperate to mediate the development of DILI induced by emodin. Here, we show that emodin can promote the occurrence of oxidative stress by impairment of fatty acid β-oxidation, TCA cycle, and OXPHOS as well as an imbalance in the expression of antioxidant enzymes. Hence, we prove a functional relationship between two major pathways driving the development of DILI and also provide additional insight into how emodin affected the mitochondrial metabolic process.

Serum ALT and AST are important enzyme indexes in liver disease screening. Analysis of serum samples from rats in the control and emodin groups indicated that emodin induced liver injury (Yang et al., 2018). Previous studies have also found that emodin induced liver injury *in vivo* and *in vitro* to some extent (Li et al., 2012; Zhang et al., 2010; Liu et al., 2015b; Ma et al., 2015; Jiang et al., 2017), similar to our observation.

Mitochondrial is an appealing target, as it plays an important role in the regulation of redox balance, which is crucial for cell life and death (Smith et al., 2012). Our proteomic analysis revealed emodin affected the redox balance by interfering with fatty acid β-oxidation, TCA cycle, and OXPHOS in mitochondria. Coenzyme A dehydrogenase and L-β-Hydroxyl acyl CoA dehydrogenase are involved in the fatty acid beta-oxidation pathway, which can catalyze dehydrogenation of fatty acyl CoA with FAD as auxiliary group and oxidize L-beta-hydroxyalkyl CoA at the same time, generating FADH_2_ and NADH. CoA produced by fat degradation is further oxidized by TCA cycle, reducing NADH and NAD ^+^ to FADH_2_ and NADH at the same time. Specifically, emodin bound to acadvl protein inhibited the normal expressions of downstream products. To adapt to this change, the body would appropriately down-regulate the protein expressions of key catalytic enzymes involved in downstream cascades, including L-β-Hydroxyl acyl CoA dehydrogenase, citrate synthase, isocitrate dehydrogenase, and succinate dehydrogenase, which were down-regulated in the proteomic data (**Table S3**, **S5**). Furthermore, it inhibited fatty acid β-oxidation, TCA cycle in downstream. The cascade feedback eventually caused the protein expression of CoA dehydrogenase to be down-regulated. Besides, the results of this cascade feedback indicated the decrease in NADH and FADH2 and the deformity of electron and energy production in mitochondria. In addition, our previous studies have found that emodin inhibited the transport of NADH and FADH2 to mitochondria (Yang et al., 2018). Moreover, the loss of hepatic fatty acid β-oxidation alters systemic lipid metabolism, which may be related to our previous studies that emodin can cause lipid accumulation in hepatocytes (Zhang et al., 2019). Electrons produced in the process of fatty acid beta-oxidation and TCA cycle are not only transferred to O_2_ *via* complex I~IV but also drive proton transfer and ATP production.

Complex IV is a complex at the distal end of the mitochondrial respiratory chain, which can transfer a pair of hydrogen atoms removed from metabolism to oxygen to generate H_2_O. Emodin passed through the outer membrane of mitochondria and acted on complex IV which was attached to the mitochondria inner membrane. On the one hand, it suppressed electron transfer of the respiratory chain, leading to a decrease in the production of intracellular ubiquinone, which in turn led to an increase in ROS (Kim et al., 2003). On the other hand, it inhibited the function of the mitochondrial respiratory chain, causing the down-regulation of protein expressions of the respiratory chain complex, which were reflected in the proteomics data (**Table S4**). It caused the decrease of mitochondrial maximal respiration and spare respiratory capacity and the increase of proton leakage (**Figure 3C**). ATP synthase is responsible for transferring protons out of the inner membrane to generate ATP. The down-regulation of ATP synthase restrained mitochondrial ATP production (**Figure 3C**). These findings suggested that emodin might interfere with fatty acid β-oxidation, TCA cycle, and OXPHOS by targeting to acadvl and complex IV.

Mitochondrial dysfunction may result in an excessive production of ROS, which was detected in the livers of the emodin group (**Figure 4A**). In order to maintain redox homeostasis, cells produce small molecule antioxidants as well as various antioxidant proteins (Walsh et al., 2009). H_2_O_2_ can be degraded by peroxiredoxins (Prdx) or by glutathione peroxidases (Gpx) which use thioredoxin (Trx) or GSH as electron donors (Finkel, 2003). Trx and glutaredoxin (Glrx) are important thiol-disulfide bond oxidoreductase which are responsible for the reduction of disulfide bonds in target proteins and are important components in the thioredoxin system and the glutamate reductase system. In the down-regulated proteins, 11 proteins related to redox regulatory were found (**Table S6**), which increased susceptibility to oxidative stress and contributed to the disruption of redox homeostasis and ultimately damaged biomolecules (Liu et al., 2009). Previous studies have found that emodin could induce oxidative stress by producing ROS and consuming antioxidants such as GSH and SOD (Ma et al., 2015; Jiang et al., 2017; Jiang et al., 2018). However, in this study, emodin could inhibit Prdx, Gpx, thioredoxin system and glutaredoxin system, which is a new discovery.

**Figure 6:**
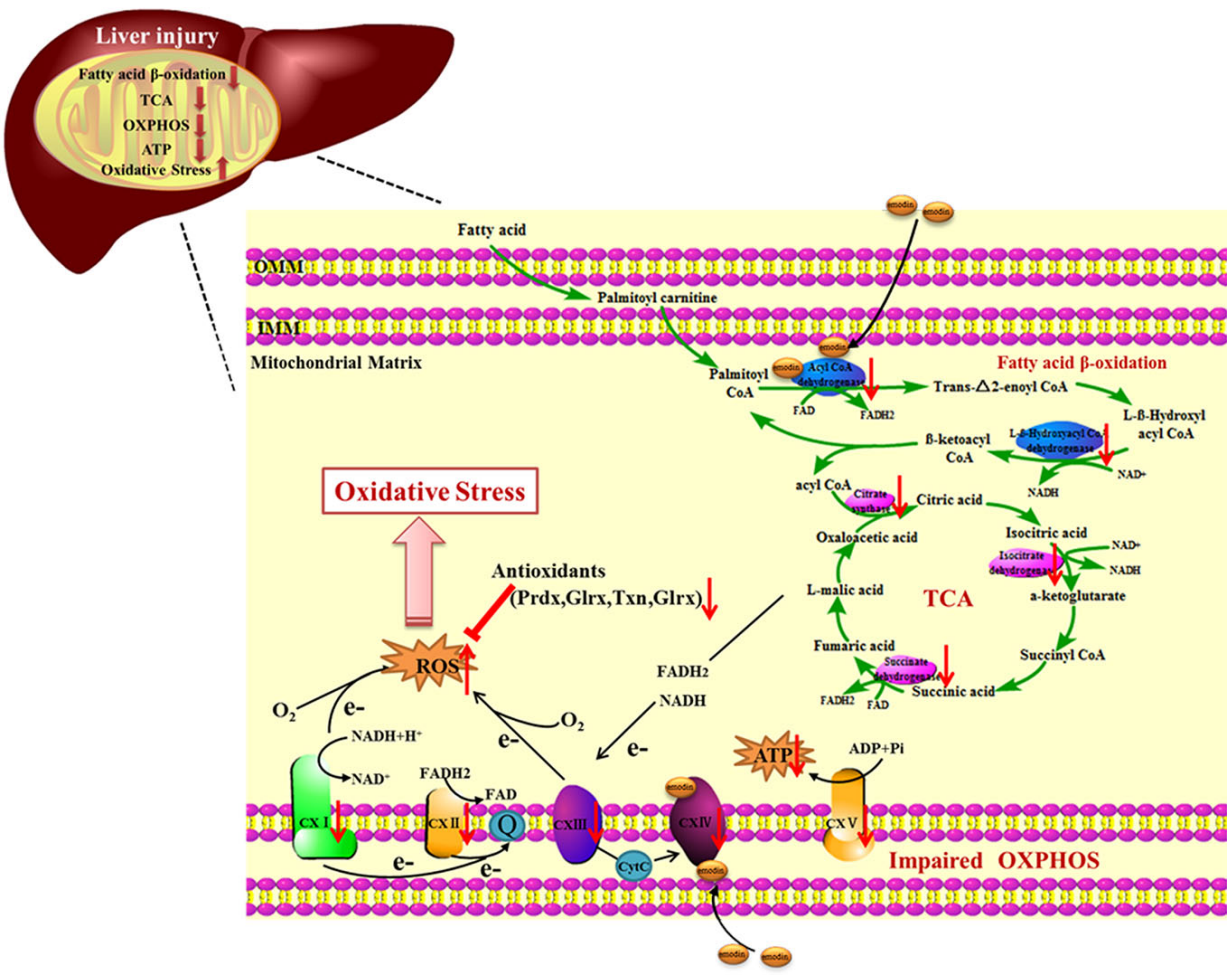
The proposed mechanism of liver injury induced by emodin in SD rats.

Mitochondria are crucial for cell function and viability, and play a central role in ROS generation (Cherukuri and Nelson, 2008; Chatterjee et al., 2008). Mitochondrial respiratory chain has been shown to be involved in ROS generation (Poyton et al., 2009). However, it remains to be determined how mitochondria metabolism initiates the generation of ROS under the influence of emodin. There are reports showing that emodin can induce suppression of respiratory chain complex function and cause mitochondrial dysfunction (Lin et al., 2019). Our results supported the fact that emodin, through targeting to acadvl and complex IV, impaired mitochondrial function by interfering with fatty acid β-oxidation, TCA cycle, and OXPHOS. The damage to mitochondrial function and imbalance of expressions of antioxidant enzymes could lead to further overproduction of ROS, which in turn leads to oxidative damage (**Figure 6**). Extensive research has shown that oxidative stress can lead to the development of chronic inflammation (Goldstein et al., 1992), which was revealed by histopathological evaluation.

## Conclusion

In this study, we aimed to explore the hepatotoxicity mechanisms of emodin on rats. Results showed that emodin (150 mg/kg daily) exposure for four weeks in SD rats could cause liver injury. Our experiments proved that oxidative stress injury caused by emodin may be the result of some deficiency in mitochondrial function, including fatty acid β-oxidation, TCA cycle, OXPHOS, and antioxidant defenses in rats *via* targeting to bind acadvl and complex IV.

## Data Availability Statement

The original contributions presented in the study are included in the article/supplementary files, further inquiries can be directed to the corresponding author.

## Ethics Statement

All the animal experiments were approved by the Committee on Animal Care and Use of Institute of Chinese Materia Medica, China Academy of Chinese Medical Sciences (Beijing, China). This study was carried out in accordance with the principles of the Basel Declaration and recommendations of guidelines of the National Institutes of Health Conflict of Interest.

## Author Contributions

YZ, XWY, and HX designed the experiments. YZ and XWY carried out the experiments. YZ and XWY analyzed the data. ZJ, JL, XNY, and YD contributed reagent/materials./analysis tools. YZ and HX wrote the manuscript.

## Funding

The project was financially supported by the National Science and Technology Major Project (2019ZX09201004-001) and the National Natural Science Foundation of China (NO. 81573839 and NO. 81774155). We thank The Center of Biomedical Analysis, Tsinghua University for the help.

## Conflict of Interest

The authors declare that the research was conducted in the absence of any commercial or financial relationships that could be construed as a potential conflict of interest.

## Supplementary Material

The Supplementary Material for this article can be foundonline at: https://www.frontiersin.org/articles/10.3389/fphar.2020.00416/full#supplementary-material

## Abbreviations

DILI, drug-induced liver injury; ATP, adenosine triphosphate; TCA, tricarboxylic acid; OXPHOS, oxidative phosphorylation; ROS, reactive oxygen species; ALT, Serum alanine aminotransferase; AST, aspartate transaminase; HE, hematoxylin and eosin; SDS-PAGE, sodium dodecyl sulfate polyacrylamide gel electrophoresis; ECL, enhanced chemiluminescence; OCR, oxygen consumption rate; FCCP, carbonyl cyanide 4-(trifluoromethoxy) phenylhydrazone; Acads, Short-chain specific acyl-CoA dehydrogenase; Hadhb, Trifunctional enzyme subunit alpha; Ndufs5, NADH dehydrogenase (Ubiquinone) Fe-S protein 5; Sdhb, Succinate dehydrogenase [ubiquinone] iron-sulfur subunit; Uqcrh, ubiquinol-cytochrome c reductase hinge protein; Cox5a, Cytochrome c oxidase subunit 5A; Atp5g1, ATP synthase F(0) complex subunit C1G1; Cs, Citrate synthase; Idh3a, Isocitrate dehydrogenase [NAD] subunit alpha; Trx, thioredoxin; Prdx, Peroxiredoxins; Gpx, glutathione peroxidases; Glrx, Glutaredoxin.
