# Supplementary material for: Proteomics Unravels Emodin Causes Liver Oxidative Damage Elicited by Mitochondrial Dysfunction
Source: Front Pharmacol. 2020 Apr 29;11:416. doi: 10.3389/fphar.2020.00416 (PMC7201015; doi:10.3389/fphar.2020.00416)
Supplement: Supplementary file 1 [file DataSheet_1.pdf]

Table S1 892 differentially expressed proteins.

Accession	Description	D1	D2	D3	K1	K2	K3
P80254	D-dopachrome decarboxylase OS=Rattus norvegicus GN=Ddt PE=1 SV=3 - [DOPD_RAT]	2E+10	1E+10	2E+10	4E+10	3E+10	3E+10
P52759	Ribonuclease UK114 OS=Rattus norvegicus GN=Hrsp12 PE=1 SV=3 - [UK114_RAT]	2E+10	2E+10	4E+09	7E+10	3E+10	6E+10
P02091	Hemoglobin subunit beta-1 OS=Rattus norvegicus GN=Hbb PE=1 SV=3 - [HBB1_RAT]	9E+10	5E+10	9E+10	1E+11	2E+11	1E+11
P11517	Hemoglobin subunit beta-2 OS=Rattus norvegicus PE=1 SV=2 - [HBB2_RAT]	9E+10	4E+10	8E+10	1E+11	1E+11	1E+11
P31044	Phosphatidylethanolamine-binding protein 1 OS=Rattus norvegicus GN=Pebp1 PE=1 SV=3 - [PEBP1_RAT]	4E+09	4E+09	4E+09	1E+10	8E+09	8E+09
D3ZEN5	Peroxiredoxin-5, mitochondrial (Fragment) OS=Rattus norvegicus GN=Prdx5 PE=4 SV=2 - [D3ZEN5_RAT]	2E+09	4E+09	5E+09	1E+10	1E+10	9E+09
P01946	Hemoglobin subunit alpha-1/2 OS=Rattus norvegicus GN=Hba1 PE=1 SV=3 - [HBA_RAT]	9E+10	1E+11	1E+11	2E+11	3E+11	2E+11
P61459	Pterin-4-alpha-carbinolamine dehydratase OS=Rattus norvegicus GN=Pcbd1 PE=1 SV=2 - [PHS_RAT]	2E+09	2E+09	2E+09	4E+09	4E+09	4E+09
Q6AYW2	Phenylalanine hydroxylase OS=Rattus norvegicus GN=Pah PE=2 SV=1 - [Q6AYW2_RAT]	7E+09	6E+09	3E+09	1E+10	1E+10	1E+10
Q66HT1	Fructose-bisphosphate aldolase OS=Rattus norvegicus GN=Aldob PE=2 SV=1 - [Q66HT1_RAT]	5E+10	3E+10	2E+10	6E+10	5E+10	6E+10
P04762	Catalase OS=Rattus norvegicus GN=Cat PE=1 SV=3 - [CATA_RAT]	3E+10	3E+10	2E+10	4E+10	5E+10	4E+10
P07824	Arginase-1 OS=Rattus norvegicus GN=Arg1 PE=1 SV=2 - [ARGI1_RAT]	2E+10	1E+10	6E+09	4E+10	3E+10	3E+10
P04642	L-lactate dehydrogenase A chain OS=Rattus norvegicus GN=Ldha PE=1 SV=1 - [LDHA_RAT]	1E+10	9E+09	5E+09	2E+10	1E+10	2E+10
V9GZ85	Protein LOC100361457 (Fragment) OS=Rattus norvegicus GN=LOC100361457 PE=3 SV=1 - [V9GZ85_RAT]	3E+10	3E+10	2E+10	7E+10	6E+10	5E+10
P04176	Phenylalanine-4-hydroxylase OS=Rattus norvegicus GN=Pah PE=1 SV=3 - [PH4H_RAT]	7E+09	6E+09	3E+09	1E+10	1E+10	1E+10
P17988	Sulfotransferase 1A1 OS=Rattus norvegicus GN=Sult1a1 PE=1 SV=1 - [ST1A1_RAT]	7E+09	7E+09	4E+09	1E+10	9E+09	1E+10
P24329	Thiosulfate sulfurtransferase OS=Rattus norvegicus GN=Tst PE=1 SV=3 - [THTR_RAT]	3E+10	2E+10	1E+10	4E+10	4E+10	4E+10
O09171	Betaine--homocysteine S-methyltransferase 1 OS=Rattus norvegicus GN=Bhmt PE=1 SV=1 - [BHMT1_RAT]	5E+10	3E+10	2E+10	7E+10	7E+10	1E+11
P09034	Argininosuccinate synthase OS=Rattus norvegicus GN=Ass1 PE=2 SV=1 - [ASSY_RAT]	2E+10	1E+10	1E+10	3E+10	2E+10	3E+10
P22734-2	Isoform 2 of Catechol O-methyltransferase OS=Rattus norvegicus GN=Comt - [COMT_RAT]	7E+09	7E+09	2E+09	2E+10	2E+10	3E+10
P00481	Ornithine carbamoyltransferase, mitochondrial OS=Rattus norvegicus GN=Otc PE=1 SV=1 - [OTC_RAT]	2E+10	2E+10	8E+09	4E+10	3E+10	3E+10
Q62669	Protein Hbb-b1 OS=Rattus norvegicus GN=Hbb-b1 PE=3 SV=1 - [Q62669_RAT]	7E+09	3E+09	3E+09	1E+10	1E+10	8E+09
Q9JLJ3	4-trimethylaminobutyraldehyde dehydrogenase OS=Rattus norvegicus GN=Aldh9a1 PE=1 SV=1 - [AL9A1_RAT]	1E+10	1E+10	7E+09	1E+10	1E+10	2E+10
P36972	Adenine phosphoribosyltransferase OS=Rattus norvegicus GN=Aprt PE=1 SV=1 - [APT_RAT]	7E+08	8E+08	1E+09	2E+09	1E+09	2E+09
Q64640	Adenosine kinase OS=Rattus norvegicus GN=Adk PE=1 SV=3 - [ADK_RAT]	5E+09	4E+09	2E+09	8E+09	7E+09	9E+09
P02692	Fatty acid-binding protein, liver OS=Rattus norvegicus GN=Fabp1 PE=1 SV=1 - [FABPL_RAT]	1E+10	3E+10	2E+10	4E+10	4E+10	3E+10
P32755	4-hydroxyphenylpyruvate dioxygenase OS=Rattus norvegicus GN=Hpd PE=1 SV=3 - [HPPD_RAT]	6E+09	6E+09	3E+09	1E+10	1E+10	8E+09
P06214	Delta-aminolevulinic acid dehydratase OS=Rattus norvegicus GN=Alad PE=1 SV=1 - [HEM2_RAT]	2E+09	1E+09	6E+08	4E+09	4E+09	3E+09
Q9ESH6	Glutaredoxin-1 OS=Rattus norvegicus GN=Glrx PE=3 SV=3 - [GLRX1_RAT]	2E+08	8E+07	1E+08	3E+08	3E+08	3E+08
P27867	Sorbitol dehydrogenase OS=Rattus norvegicus GN=Sord PE=2 SV=4 - [DHSO_RAT]	6E+09	6E+09	4E+09	1E+10	1E+10	1E+10
P62963	Profilin-1 OS=Rattus norvegicus GN=Pfn1 PE=1 SV=2 - [PROF1_RAT]	2E+09	2E+09	3E+09	9E+09	8E+09	6E+09
P00884	Fructose-bisphosphate aldolase B OS=Rattus norvegicus GN=Aldob PE=1 SV=2 - [ALDOB_RAT]	5E+10	3E+10	2E+10	6E+10	5E+10	6E+10
D3ZXY4	Protein Aldh8a1 OS=Rattus norvegicus GN=Aldh8a1 PE=3 SV=2 - [D3ZXY4_RAT]	1E+10	1E+10	9E+09	2E+10	2E+10	3E+10
P19112	Fructose-1,6-bisphosphatase 1 OS=Rattus norvegicus GN=Fbp1 PE=1 SV=2 - [F16P1_RAT]	9E+09	8E+09	4E+09	2E+10	1E+10	1E+10
P23680	Serum amyloid P-component OS=Rattus norvegicus GN=Apcs PE=2 SV=2 - [SAMP_RAT]	7E+08	7E+08	2E+08	2E+09	1E+09	1E+09
P07379	Phosphoenolpyruvate carboxykinase, cytosolic [GTP] OS=Rattus norvegicus GN=Pck1 PE=1 SV=1 - [PCKGC_RAT]	2E+09	4E+09	2E+09	6E+09	7E+09	6E+09
P63018	Heat shock cognate 71 kDa protein OS=Rattus norvegicus GN=Hspa8 PE=1 SV=1 - [HSP7C_RAT]	8E+09	1E+10	8E+09	2E+10	2E+10	2E+10
P31399	ATP synthase subunit d, mitochondrial OS=Rattus norvegicus GN=Atp5h PE=1 SV=3 - [ATP5H_RAT]	2E+09	1E+09	1E+09	4E+09	5E+09	4E+09
P50137	Transketolase OS=Rattus norvegicus GN=Tkt PE=1 SV=1 - [TKT_RAT]	8E+09	8E+09	6E+09	1E+10	2E+10	1E+10
P84079	ADP-ribosylation factor 1 OS=Rattus norvegicus GN=Arf1 PE=1 SV=2 - [ARF1_RAT]	7E+08	7E+08	4E+08	2E+09	2E+09	2E+09
P00507	Aspartate aminotransferase, mitochondrial OS=Rattus norvegicus GN=Got2 PE=1 SV=2 - [AATM_RAT]	2E+10	2E+10	1E+10	4E+10	4E+10	3E+10
P61751	ADP-ribosylation factor 4 OS=Rattus norvegicus GN=Arf4 PE=2 SV=2 - [ARF4_RAT]	7E+08	7E+08	4E+08	2E+09	1E+09	2E+09
P08683	Cytochrome P450 2C11 OS=Rattus norvegicus GN=Cyp2c11 PE=1 SV=1 - [CP2CB_RAT]	7E+09	4E+09	3E+09	1E+10	9E+09	1E+10
Q6AYS8	Estradiol 17-beta-dehydrogenase 11 OS=Rattus norvegicus GN=Hsd17b11 PE=2 SV=1 - [DHB11_RAT]	7E+08	5E+08	2E+08	1E+09	9E+08	8E+08
Q5XIF6	Tubulin alpha-4A chain OS=Rattus norvegicus GN=Tuba4a PE=2 SV=1 - [TBA4A_RAT]	9E+09	7E+09	4E+09	1E+10	1E+10	1E+10
P04041	Glutathione peroxidase 1 OS=Rattus norvegicus GN=Gpx1 PE=1 SV=4 - [GPX1_RAT]	5E+09	4E+09	3E+09	9E+09	6E+09	1E+10
Q68G44	3-hydroxy-3-methylglutaryl-Coenzyme A synthase 2 (Mitochondrial) OS=Rattus norvegicus GN=Hmgcs2 PE=2 SV=1 - [Q68G44_RAT]	3E+10	3E+10	2E+10	5E+10	6E+10	4E+10
O88767	Protein DJ-1 OS=Rattus norvegicus GN=Park7 PE=1 SV=1 - [PARK7_RAT]	2E+09	2E+09	1E+09	4E+09	2E+09	5E+09
P57113	Maleylacetoacetate isomerase OS=Rattus norvegicus GN=Gstz1 PE=1 SV=2 - [MAAI_RAT]	4E+09	3E+09	2E+09	8E+09	5E+09	7E+09
Q68FP2	Serum paraoxonase/lactonase 3 OS=Rattus norvegicus GN=Pon3 PE=2 SV=1 - [PON3_RAT]	9E+08	9E+08	5E+08	2E+09	2E+09	2E+09
P19804	Nucleoside diphosphate kinase B OS=Rattus norvegicus GN=Nme2 PE=1 SV=1 - [NDKB_RAT]	2E+09	2E+09	2E+09	4E+09	4E+09	4E+09
P08011	Microsomal glutathione S-transferase 1 OS=Rattus norvegicus GN=Mgst1 PE=1 SV=3 - [MGST1_RAT]	5E+09	4E+09	2E+09	6E+09	8E+09	1E+10

Q9WUC4	Copper transport protein ATOX1 OS=Rattus norvegicus GN=Atox1 PE=1 SV=1 - [ATOX1_RAT]	3E+08	2E+08	0	7E+08	3E+08	7E+08
P02696	Retinol-binding protein 1 OS=Rattus norvegicus GN=Rbp1 PE=1 SV=2 - [RET1_RAT]	6E+07	3E+08	2E+08	5E+08	9E+08	6E+08
D3ZCS9	Protein LOC100911028 OS=Rattus norvegicus GN=RGD1309350 PE=3 SV=1 - [D3ZCS9_RAT]	7E+08	6E+08	1E+09	2E+09	2E+09	2E+09
P10760	Adenosylhomocysteinase OS=Rattus norvegicus GN=Ahcy PE=1 SV=3 - [SAHH_RAT]	7E+09	7E+09	4E+09	1E+10	9E+09	1E+10
B0BN46	Grhpr protein OS=Rattus norvegicus GN=Grhpr PE=2 SV=1 - [B0BN46_RAT]	4E+09	3E+09	2E+09	7E+09	6E+09	6E+09
F1LZW6	Protein Slc25a13 (Fragment) OS=Rattus norvegicus GN=Slc25a13 PE=3 SV=1 - [F1LZW6_RAT]	1E+09	1E+09	1E+09	2E+09	2E+09	2E+09
M0RAK2	Protein Isoc2a OS=Rattus norvegicus GN=Isoc2a PE=4 SV=1 - [M0RAK2_RAT]	7E+08	6E+08	2E+08	2E+09	1E+09	2E+09
Q63342	Dimethylglycine dehydrogenase, mitochondrial OS=Rattus norvegicus GN=Dmgdh PE=1 SV=1 - [M2GD_RAT]	5E+09	8E+09	5E+09	1E+10	1E+10	1E+10
G3V826	Transketolase OS=Rattus norvegicus GN=Tkt PE=3 SV=1 - [G3V826_RAT]	8E+09	8E+09	6E+09	1E+10	2E+10	1E+10
P62775	Myotrophin OS=Rattus norvegicus GN=Mtpn PE=1 SV=2 - [MTPN_RAT]	5E+08	4E+08	4E+08	1E+09	9E+08	1E+09
Q66HG4	Aldose 1-epimerase OS=Rattus norvegicus GN=Galm PE=1 SV=1 - [GALM_RAT]	1E+09	9E+08	5E+08	2E+09	1E+09	2E+09
P46462	Transitional endoplasmic reticulum ATPase OS=Rattus norvegicus GN=Vcp PE=1 SV=3 - [TERA_RAT]	4E+09	6E+09	5E+09	7E+09	7E+09	7E+09
Q63362	NADH dehydrogenase [ubiquinone] 1 alpha subcomplex subunit 5 OS=Rattus norvegicus GN=Ndufa5 PE=1 SV=3 - [NDUA5_RAT]	3E+08	2E+08	1E+08	7E+08	5E+08	6E+08
O88618	Formimidoyltransferase-cyclodeaminase OS=Rattus norvegicus GN=Ftcd PE=1 SV=4 - [FTCD_RAT]	5E+09	5E+09	4E+09	8E+09	8E+09	8E+09
P70552	GTP cyclohydrolase 1 feedback regulatory protein OS=Rattus norvegicus GN=Gchfr PE=1 SV=3 - [GFRP_RAT]	1E+08	1E+08	4E+07	4E+08	3E+08	4E+08
Q5RKL4	Dimethylglycine dehydrogenase OS=Rattus norvegicus GN=Dmgdh PE=2 SV=1 - [Q5RKL4_RAT]	5E+09	8E+09	5E+09	1E+10	1E+10	1E+10
D3ZGY4	Glyceraldehyde-3-phosphate dehydrogenase OS=Rattus norvegicus GN=RGD1560797 PE=3 SV=1 - [D3ZGY4_RAT]	2E+10	2E+10	8E+09	3E+10	3E+10	4E+10
P18418	Calreticulin OS=Rattus norvegicus GN=Calr PE=1 SV=1 - [CALR_RAT]	9E+09	1E+10	9E+09	2E+10	2E+10	2E+10
P16617	Phosphoglycerate kinase 1 OS=Rattus norvegicus GN=Pgk1 PE=1 SV=2 - [PGK1_RAT]	9E+09	9E+09	4E+09	2E+10	2E+10	1E+10
P52873	Pyruvate carboxylase, mitochondrial OS=Rattus norvegicus GN=Pc PE=1 SV=2 - [PYC_RAT]	1E+10	1E+10	1E+10	2E+10	2E+10	2E+10
Q4KLP0	Probable 2-oxoglutarate dehydrogenase E1 component DHKTD1, mitochondrial OS=Rattus norvegicus GN=Dhtkd1 PE=2 SV=1 - [DHTK1_RAT]	2E+09	5E+09	3E+09	6E+09	6E+09	6E+09
P11598	Protein disulfide-isomerase A3 OS=Rattus norvegicus GN=Pdia3 PE=1 SV=2 - [PDIA3_RAT]	8E+09	7E+09	5E+09	1E+10	1E+10	1E+10
Q4QQW3	Hydroxyacid-oxoacid transhydrogenase, mitochondrial OS=Rattus norvegicus GN=Adhfe1 PE=1 SV=1 - [HOT_RAT]	1E+09	2E+09	7E+08	3E+09	2E+09	3E+09
G3V8V0	ADP-ribosylation factor-like 2, isoform CRA_a OS=Rattus norvegicus GN=Arl2 PE=3 SV=1 - [G3V8V0_RAT]	7E+06	2E+07	5E+07	7E+07	5E+07	7E+07
Q4KLZ6	Bifunctional ATP-dependent dihydroxyacetone kinase/FAD-AMP lyase (cyclizing) OS=Rattus norvegicus GN=Dak PE=1 SV=1 - [DHAK_RAT]	3E+09	4E+09	4E+09	8E+09	5E+09	6E+09
Q8CHM7	2-hydroxyacyl-CoA lyase 1 OS=Rattus norvegicus GN=Hacl1 PE=1 SV=1 - [HACL1_RAT]	3E+09	3E+09	2E+09	4E+09	5E+09	5E+09
P62630	Elongation factor 1-alpha 1 OS=Rattus norvegicus GN=Eef1a1 PE=1 SV=1 - [EF1A1_RAT]	7E+09	1E+10	1E+10	2E+10	2E+10	2E+10
P67779	Prohibitin OS=Rattus norvegicus GN=Phb PE=1 SV=1 - [PHB_RAT]	3E+09	2E+09	2E+09	5E+09	4E+09	4E+09
G3V6C4	UDP-glucose 6-dehydrogenase OS=Rattus norvegicus GN=Ugdh PE=3 SV=1 - [G3V6C4_RAT]	2E+09	2E+09	2E+09	3E+09	4E+09	4E+09
Q6PDW4	Proteasome subunit beta type OS=Rattus norvegicus GN=Psmb1 PE=2 SV=1 - [Q6PDW4_RAT]	2E+08	2E+08	2E+08	1E+09	6E+08	8E+08
D4AAV1	Protein Amdhd1 OS=Rattus norvegicus GN=Amdhd1 PE=4 SV=1 - [D4AAV1_RAT]	1E+09	2E+09	1E+09	4E+09	3E+09	4E+09
F7EVC6	Protein Hebp1 OS=Rattus norvegicus GN=Hebp1 PE=4 SV=1 - [F7EVC6_RAT]	4E+08	4E+08	4E+08	7E+08	6E+08	7E+08
Q5HZY2	GTP-binding protein SAR1b OS=Rattus norvegicus GN=Sar1b PE=2 SV=1 - [SAR1B_RAT]	7E+08	6E+08	4E+08	2E+09	1E+09	2E+09
P05178	Cytochrome P450 2C6 OS=Rattus norvegicus GN=Cyp2c6 PE=2 SV=2 - [CP2C6_RAT]	4E+09	2E+09	2E+09	4E+09	4E+09	4E+09
P21775	3-ketoacyl-CoA thiolase A, peroxisomal OS=Rattus norvegicus GN=Acaa1a PE=1 SV=2 - [THIKA_RAT]	4E+09	3E+09	1E+09	6E+09	8E+09	5E+09
P63029	Translationally-controlled tumor protein OS=Rattus norvegicus GN=Tpt1 PE=1 SV=1 - [TCTP_RAT]	6E+08	6E+08	8E+07	1E+09	9E+08	1E+09
M0R8Q2	Protein LOC100360791 OS=Rattus norvegicus GN=LOC100360791 PE=4 SV=1 - [M0R8Q2_RAT]	6E+08	5E+08	8E+07	1E+09	9E+08	1E+09
Q64573	Liver carboxylesterase 4 OS=Rattus norvegicus PE=2 SV=2 - [EST4_RAT]	6E+09	1E+10	7E+09	1E+10	1E+10	2E+10
D4A830	Protein Ppa2 OS=Rattus norvegicus GN=Ppa2 PE=4 SV=1 - [D4A830_RAT]	8E+08	5E+08	3E+08	1E+09	1E+09	1E+09
B2RZA9	Protein Ube2l3 OS=Rattus norvegicus GN=Ube2l3 PE=2 SV=1 - [B2RZA9_RAT]	4E+08	4E+08	6E+08	1E+09	1E+09	1E+09
B0BNJ4	Ethylmalonic encephalopathy 1 OS=Rattus norvegicus GN=Ethe1 PE=2 SV=1 - [B0BNJ4_RAT]	2E+09	2E+09	8E+08	6E+09	3E+09	5E+09
P15651	Short-chain specific acyl-CoA dehydrogenase, mitochondrial OS=Rattus norvegicus GN=Acads PE=1 SV=2 - [ACADS_RAT]	3E+09	3E+09	2E+09	9E+09	7E+09	5E+09
Q3T1J1	Eukaryotic translation initiation factor 5A-1 OS=Rattus norvegicus GN=Eif5a PE=1 SV=3 - [IF5A1_RAT]	8E+08	6E+08	7E+08	2E+09	2E+09	1E+09
O35567	Bifunctional purine biosynthesis protein PURH OS=Rattus norvegicus GN=Atic PE=1 SV=2 - [PUR9_RAT]	6E+08	6E+08	5E+08	1E+09	1E+09	1E+09
P06761	78 kDa glucose-regulated protein OS=Rattus norvegicus GN=Hspa5 PE=1 SV=1 - [GRP78_RAT]	1E+10	2E+10	1E+10	2E+10	3E+10	2E+10
Q9EQX9	Ubiquitin-conjugating enzyme E2 N OS=Rattus norvegicus GN=Ube2n PE=1 SV=1 - [UBE2N_RAT]	5E+08	6E+08	7E+08	2E+09	2E+09	9E+08
D3ZZR9	Peptidyl-prolyl cis-trans isomerase OS=Rattus norvegicus GN=Fkbp2 PE=4 SV=1 - [D3ZZR9_RAT]	6E+08	8E+08	1E+09	2E+09	2E+09	2E+09
P18163	Long-chain-fatty-acid--CoA ligase 1 OS=Rattus norvegicus GN=Acsl1 PE=1 SV=1 - [ACSL1_RAT]	7E+09	8E+09	5E+09	1E+10	1E+10	1E+10
Q6PDV6	40S ribosomal protein S14 OS=Rattus norvegicus GN=Rps14 PE=2 SV=1 - [Q6PDV6_RAT]	6E+08	6E+08	8E+08	2E+09	2E+09	1E+09
P23358	60S ribosomal protein L12 OS=Rattus norvegicus GN=Rpl12 PE=2 SV=1 - [RL12_RAT]	5E+08	5E+08	5E+08	2E+09	2E+09	2E+09
P68035	Actin, alpha cardiac muscle 1 OS=Rattus norvegicus GN=Actc1 PE=2 SV=1 - [ACTC_RAT]	1E+10	1E+10	9E+09	3E+10	2E+10	2E+10
M0RC77	Kynurenine formamidase OS=Rattus norvegicus GN=Afmid PE=3 SV=1 - [M0RC77_RAT]	2E+09	1E+09	6E+08	3E+09	2E+09	2E+09
P26772	10 kDa heat shock protein, mitochondrial OS=Rattus norvegicus GN=Hspe1 PE=1 SV=3 - [CH10_RAT]	1E+09	2E+09	2E+09	5E+09	5E+09	4E+09
P20814	Cytochrome P450 2C13, male-specific OS=Rattus norvegicus GN=Cyp2c13 PE=1 SV=1 - [CP2CD_RAT]	3E+09	3E+09	1E+09	5E+09	5E+09	4E+09
Q6P6R2	Dihydrolipoyl dehydrogenase, mitochondrial OS=Rattus norvegicus GN=Dld PE=1 SV=1 - [DLDH_RAT]	3E+09	3E+09	2E+09	4E+09	5E+09	5E+09
P70473	Alpha-methylacyl-CoA racemase OS=Rattus norvegicus GN=Amacr PE=1 SV=3 - [AMACR_RAT]	3E+09	3E+09	2E+09	1E+10	7E+09	5E+09

F8WFI0	Taste receptor type 1 member 2 OS=Rattus norvegicus GN=Aldh4a1 PE=3 SV=2 - [F8WFI0_RAT]	1E+10	2E+10	1E+10	2E+10	2E+10	3E+10
Q5M9H2	Acyl-Coenzyme A dehydrogenase, very long chain OS=Rattus norvegicus GN=Acadvl PE=2 SV=1 - [Q5M9H2_RAT]	2E+09	3E+09	2E+09	5E+09	7E+09	6E+09
Q6UPE0	Choline dehydrogenase, mitochondrial OS=Rattus norvegicus GN=Chdh PE=1 SV=1 - [CHDH_RAT]	5E+09	4E+09	3E+09	7E+09	7E+09	9E+09
Q5XIT9	Methylcrotonoyl-CoA carboxylase beta chain, mitochondrial OS=Rattus norvegicus GN=Mccc2 PE=2 SV=1 - [MCCB_RAT]	1E+09	1E+09	8E+08	2E+09	2E+09	2E+09
Q9WUS0	Adenylate kinase 4, mitochondrial OS=Rattus norvegicus GN=Ak4 PE=2 SV=1 - [KAD4_RAT]	2E+09	2E+09	2E+09	3E+09	3E+09	3E+09
P62982	Ubiquitin-40S ribosomal protein S27a OS=Rattus norvegicus GN=Rps27a PE=1 SV=2 - [RS27A_RAT]	2E+09	2E+09	2E+09	8E+09	8E+09	6E+09
P07871	3-ketoacyl-CoA thiolase B, peroxisomal OS=Rattus norvegicus GN=Acaa1b PE=1 SV=2 - [THIKB_RAT]	4E+09	3E+09	1E+09	6E+09	8E+09	5E+09
P10111	Peptidyl-prolyl cis-trans isomerase A OS=Rattus norvegicus GN=Ppia PE=1 SV=2 - [PPIA_RAT]	3E+09	3E+09	1E+09	1E+10	7E+09	1E+10
D4A8D3	Ferritin OS=Rattus norvegicus GN=LOC100363177 PE=3 SV=1 - [D4A8D3_RAT]	2E+09	2E+09	9E+08	6E+09	4E+09	4E+09
P84083	ADP-ribosylation factor 5 OS=Rattus norvegicus GN=Arf5 PE=1 SV=2 - [ARF5_RAT]	7E+08	7E+08	4E+08	2E+09	1E+09	2E+09
O70199	UDP-glucose 6-dehydrogenase OS=Rattus norvegicus GN=Ugdh PE=2 SV=1 - [UGDH_RAT]	2E+09	2E+09	2E+09	3E+09	4E+09	4E+09
P61972	Nuclear transport factor 2 OS=Rattus norvegicus GN=Nutf2 PE=1 SV=1 - [NTF2_RAT]	0	2E+07	0	7E+07	5E+07	1E+08
Q66HF3	Electron transfer flavoprotein-ubiquinone oxidoreductase, mitochondrial OS=Rattus norvegicus GN=Etfdh PE=2 SV=1 - [Q66HF3_RAT]	2E+09	2E+09	2E+09	5E+09	5E+09	4E+09
Q5U204	Ragulator complex protein LAMTOR3 OS=Rattus norvegicus GN=Lamtor3 PE=2 SV=1 - [LTOR3_RAT]	2E+07	7E+07	3E+07	2E+08	1E+08	1E+08
P62332	ADP-ribosylation factor 6 OS=Rattus norvegicus GN=Arf6 PE=1 SV=2 - [ARF6_RAT]	2E+08	2E+08	2E+08	8E+08	7E+08	7E+08
Q6PEC0	Bis(5'-nucleosyl)-tetraphosphatase [asymmetrical] OS=Rattus norvegicus GN=Nudt2 PE=2 SV=3 - [AP4A_RAT]	4E+07	2E+07	4E+07	1E+08	5E+07	1E+08
P12928-2	Isoform L-type of Pyruvate kinase PKLR OS=Rattus norvegicus GN=Pklr - [KPYR_RAT]	3E+09	3E+09	2E+09	4E+09	5E+09	5E+09
P09527	Ras-related protein Rab-7a OS=Rattus norvegicus GN=Rab7a PE=1 SV=2 - [RAB7A_RAT]	4E+08	3E+08	4E+08	9E+08	7E+08	7E+08
P45592	Cofilin-1 OS=Rattus norvegicus GN=Cfl1 PE=1 SV=3 - [COF1_RAT]	1E+09	1E+09	1E+09	3E+09	3E+09	2E+09
P17764	Acetyl-CoA acetyltransferase, mitochondrial OS=Rattus norvegicus GN=Acat1 PE=1 SV=1 - [THIL_RAT]	8E+09	6E+09	3E+09	1E+10	1E+10	1E+10
Q5U3Y8	Basic transcription factor 3 OS=Rattus norvegicus GN=Btf3 PE=2 SV=1 - [Q5U3Y8_RAT]	3E+08	1E+08	1E+08	5E+08	4E+08	4E+08
D3ZCI0	Glycerol kinase OS=Rattus norvegicus GN=Gk PE=3 SV=2 - [D3ZCI0_RAT]	2E+09	2E+09	1E+09	3E+09	3E+09	3E+09
P12007	Isovaleryl-CoA dehydrogenase, mitochondrial OS=Rattus norvegicus GN=Ivd PE=1 SV=2 - [IVD_RAT]	2E+09	3E+09	2E+09	6E+09	5E+09	5E+09
Q7TPB1	T-complex protein 1 subunit delta OS=Rattus norvegicus GN=Cct4 PE=1 SV=3 - [TCPD_RAT]	5E+08	7E+08	6E+08	1E+09	1E+09	1E+09
G3V734	2,4-dienoyl CoA reductase 1, mitochondrial, isoform CRA_a OS=Rattus norvegicus GN=Decr1 PE=4 SV=1 - [G3V734_RAT]	6E+09	5E+09	4E+09	8E+09	7E+09	8E+09
Q64428	Trifunctional enzyme subunit alpha, mitochondrial OS=Rattus norvegicus GN=Hadha PE=1 SV=2 - [ECHA_RAT]	2E+09	3E+09	3E+09	6E+09	6E+09	5E+09
P29117	Peptidyl-prolyl cis-trans isomerase F, mitochondrial OS=Rattus norvegicus GN=Ppif PE=1 SV=2 - [PPIF_RAT]	3E+08	6E+08	8E+08	2E+09	2E+09	1E+09
Q60587	Trifunctional enzyme subunit beta, mitochondrial OS=Rattus norvegicus GN=Hadhb PE=1 SV=1 - [ECHB_RAT]	4E+09	4E+09	2E+09	6E+09	6E+09	6E+09
Q66X93	Staphylococcal nuclease domain-containing protein 1 OS=Rattus norvegicus GN=Snd1 PE=2 SV=1 - [SND1_RAT]	2E+09	1E+09	1E+09	2E+09	2E+09	2E+09
P35738	2-oxoisovalerate dehydrogenase subunit beta, mitochondrial OS=Rattus norvegicus GN=Bckdhb PE=1 SV=3 - [ODBB_RAT]	2E+09	1E+09	7E+08	2E+09	2E+09	2E+09
P35704	Peroxiredoxin-2 OS=Rattus norvegicus GN=Prdx2 PE=1 SV=3 - [PRDX2_RAT]	1E+09	9E+08	1E+09	2E+09	2E+09	2E+09
Q8R491	EH domain-containing protein 3 OS=Rattus norvegicus GN=Ehd3 PE=1 SV=2 - [EHD3_RAT]	5E+08	4E+08	3E+08	8E+08	1E+09	6E+08
D3ZT90	Glutaryl-Coenzyme A dehydrogenase (Predicted) OS=Rattus norvegicus GN=Gcdh PE=3 SV=1 - [D3ZT90_RAT]	3E+09	2E+09	2E+09	4E+09	4E+09	4E+09
P27364	3 beta-hydroxysteroid dehydrogenase type 5 OS=Rattus norvegicus GN=Hsd3b5 PE=2 SV=3 - [3BHS5_RAT]	2E+09	2E+09	1E+09	5E+09	3E+09	4E+09
Q6P7Q4	Lactoylglutathione lyase OS=Rattus norvegicus GN=Glo1 PE=1 SV=3 - [LGUL_RAT]	7E+08	3E+08	4E+08	1E+09	8E+08	1E+09
Q68FZ8	Propionyl coenzyme A carboxylase, beta polypeptide OS=Rattus norvegicus GN=Pccb PE=2 SV=1 - [Q68FZ8_RAT]	2E+09	2E+09	9E+08	2E+09	2E+09	3E+09
P02761	Major urinary protein OS=Rattus norvegicus PE=1 SV=1 - [MUP_RAT]	2E+09	3E+09	3E+09	1E+10	6E+09	6E+09
O08557	N(G),N(G)-dimethylarginine dimethylaminohydrolase 1 OS=Rattus norvegicus GN=Ddah1 PE=1 SV=3 - [DDAH1_RAT]	6E+08	6E+08	3E+08	1E+09	8E+08	1E+09
P48037	Annexin A6 OS=Rattus norvegicus GN=Anxa6 PE=1 SV=2 - [ANXA6_RAT]	1E+09	1E+09	9E+08	2E+09	2E+09	2E+09
B0BNL2	Peptidylprolyl cis/trans isomerase, NIMA-interacting 1 OS=Rattus norvegicus GN=LOC364561 PE=2 SV=1 - [B0BNL2_RAT]	3E+07	3E+07	5E+07	6E+07	1E+08	1E+08
P34058	Heat shock protein HSP 90-beta OS=Rattus norvegicus GN=Hsp90ab1 PE=1 SV=4 - [HS90B_RAT]	8E+09	1E+10	9E+09	1E+10	2E+10	2E+10
P62246	40S ribosomal protein S15a OS=Rattus norvegicus GN=Rps15a PE=1 SV=2 - [RS15A_RAT]	7E+08	3E+08	2E+08	1E+09	1E+09	1E+09
Q5XFX0	Transgelin-2 OS=Rattus norvegicus GN=Tagln2 PE=2 SV=1 - [TAGL2_RAT]	2E+08	2E+08	3E+08	6E+08	7E+08	7E+08
P38983	40S ribosomal protein SA OS=Rattus norvegicus GN=Rpsa PE=1 SV=3 - [RSSA_RAT]	3E+09	2E+09	1E+09	5E+09	5E+09	4E+09
P32551	Cytochrome b-c1 complex subunit 2, mitochondrial OS=Rattus norvegicus GN=Uqcrc2 PE=1 SV=2 - [QCR2_RAT]	2E+09	1E+09	7E+08	3E+09	3E+09	3E+09
Q641Z6	EH domain-containing protein 1 OS=Rattus norvegicus GN=Ehd1 PE=1 SV=1 - [EHD1_RAT]	4E+08	4E+08	3E+08	7E+08	8E+08	6E+08
B2RZD6	Ndufa4 protein OS=Rattus norvegicus GN=Ndufa4 PE=4 SV=1 - [B2RZD6_RAT]	2E+09	7E+08	1E+09	3E+09	4E+09	3E+09
Q920L2	Succinate dehydrogenase [ubiquinone] flavoprotein subunit, mitochondrial OS=Rattus norvegicus GN=Sdha PE=1 SV=1 - [SDHA_RAT]	2E+09	2E+09	2E+09	3E+09	3E+09	3E+09
Q5BJY9	Keratin, type I cytoskeletal 18 OS=Rattus norvegicus GN=Krt18 PE=1 SV=3 - [K1C18_RAT]	9E+08	1E+09	5E+08	3E+09	3E+09	3E+09
B5DFH4	Papss2 protein OS=Rattus norvegicus GN=Papss2 PE=2 SV=1 - [B5DFH4_RAT]	9E+08	1E+09	7E+08	2E+09	2E+09	2E+09
Q9Z0V5	Peroxiredoxin-4 OS=Rattus norvegicus GN=Prdx4 PE=2 SV=1 - [PRDX4_RAT]	4E+09	4E+09	3E+09	1E+10	6E+09	8E+09
P09811	Glycogen phosphorylase, liver form OS=Rattus norvegicus GN=Pygl PE=1 SV=5 - [PYGL_RAT]	3E+09	4E+09	3E+09	7E+09	6E+09	7E+09
P62909	40S ribosomal protein S3 OS=Rattus norvegicus GN=Rps3 PE=1 SV=1 - [RS3_RAT]	1E+09	8E+08	6E+08	1E+09	2E+09	2E+09
Q5XFW8	Protein SEC13 homolog OS=Rattus norvegicus GN=Sec13 PE=1 SV=1 - [SEC13_RAT]	5E+08	4E+08	2E+08	8E+08	8E+08	8E+08
F1LR47	Uncharacterized protein OS=Rattus norvegicus PE=3 SV=2 - [F1LR47_RAT]	3E+09	1E+09	1E+09	4E+09	4E+09	4E+09
P32232	Cystathionine beta-synthase OS=Rattus norvegicus GN=Cbs PE=1 SV=3 - [CBS_RAT]	2E+09	2E+09	2E+09	5E+09	3E+09	5E+09

P01836	Ig kappa chain C region, A allele OS=Rattus norvegicus PE=1 SV=1 - [KACA_RAT]	3E+08	4E+08	2E+08	8E+08	5E+08	6E+08
P51635	Alcohol dehydrogenase [NADP(+)] OS=Rattus norvegicus GN=Akr1a1 PE=1 SV=2 - [AK1A1_RAT]	3E+09	3E+09	2E+09	7E+09	6E+09	5E+09
P05544	Serine protease inhibitor A3L OS=Rattus norvegicus GN=Serpina3l PE=1 SV=3 - [SPA3L_RAT]	1E+09	1E+09	1E+09	1E+09	2E+09	1E+09
Q6AXM8	Serum paraoxonase/arylesterase 2 OS=Rattus norvegicus GN=Pon2 PE=2 SV=1 - [PON2_RAT]	5E+08	5E+08	2E+08	2E+09	1E+09	9E+08
Q5XIC0	Enoyl-CoA delta isomerase 2, mitochondrial OS=Rattus norvegicus GN=Eci2 PE=1 SV=1 - [ECI2_RAT]	2E+09	2E+09	9E+08	4E+09	4E+09	3E+09
P50398	Rab GDP dissociation inhibitor alpha OS=Rattus norvegicus GN=Gdi1 PE=1 SV=1 - [GDIA_RAT]	1E+09	1E+09	8E+08	1E+09	2E+09	2E+09
M0RC65	Cofilin 2, muscle (Predicted), isoform CRA_b OS=Rattus norvegicus GN=Cfl2 PE=4 SV=1 - [M0RC65_RAT]	8E+08	6E+08	7E+08	2E+09	2E+09	1E+09
Q63570	26S protease regulatory subunit 6B OS=Rattus norvegicus GN=Psmc4 PE=1 SV=1 - [PRS6B_RAT]	4E+08	4E+08	3E+08	5E+08	6E+08	5E+08
P56571	ES1 protein homolog, mitochondrial OS=Rattus norvegicus PE=1 SV=2 - [ES1_RAT]	2E+09	2E+09	5E+08	4E+09	2E+09	3E+09
Q66HF1	NADH-ubiquinone oxidoreductase 75 kDa subunit, mitochondrial OS=Rattus norvegicus GN=Ndufs1 PE=1 SV=1 - [NDUS1_RAT]	1E+09	1E+09	9E+08	2E+09	2E+09	2E+09
D4A465	Protein Lamtor2 OS=Rattus norvegicus GN=Lamtor2 PE=4 SV=1 - [D4A465_RAT]	1E+06	2E+07	2E+07	8E+07	6E+07	9E+07
D4AEL0	Protein LOC691083 OS=Rattus norvegicus GN=LOC691083 PE=4 SV=1 - [D4AEL0_RAT]	2E+08	2E+08	2E+08	8E+08	6E+08	8E+08
P05197	Elongation factor 2 OS=Rattus norvegicus GN=Eef2 PE=1 SV=4 - [EF2_RAT]	4E+09	4E+09	3E+09	6E+09	6E+09	5E+09
Q9QXQ0	Alpha-actinin-4 OS=Rattus norvegicus GN=Actn4 PE=1 SV=2 - [ACTN4_RAT]	1E+09	1E+09	1E+09	2E+09	2E+09	1E+09
D3ZXS8	Huntingtin interacting protein 2 (Predicted), isoform CRA_a OS=Rattus norvegicus GN=Ube2k PE=3 SV=1 - [D3ZXS8_RAT]	1E+08	1E+08	2E+08	5E+08	3E+08	4E+08
P20059	Hemopexin OS=Rattus norvegicus GN=Hpx PE=1 SV=3 - [HEMO_RAT]	1E+09	2E+09	1E+09	2E+09	3E+09	2E+09
Q6AYD9	Nucleoside diphosphate-linked moiety X motif 19, mitochondrial OS=Rattus norvegicus GN=Nudt19 PE=2 SV=1 - [NUD19_RAT]	2E+08	1E+08	9E+07	3E+08	2E+08	2E+08
G3V8F9	Alpha-methylacyl-CoA racemase OS=Rattus norvegicus GN=Amacr PE=4 SV=1 - [G3V8F9_RAT]	3E+09	3E+09	2E+09	1E+10	7E+09	5E+09
P20673	Argininosuccinate lyase OS=Rattus norvegicus GN=Asl PE=2 SV=1 - [ARLY_RAT]	4E+09	3E+09	2E+09	7E+09	6E+09	6E+09
P62870	Transcription elongation factor B polypeptide 2 OS=Rattus norvegicus GN=Tceb2 PE=1 SV=1 - [ELOB_RAT]	0	1E+08	0	4E+08	3E+08	3E+08
Q6P3V8	Eukaryotic translation initiation factor 4A1 OS=Rattus norvegicus GN=Eif4a1 PE=1 SV=1 - [Q6P3V8_RAT]	1E+09	1E+09	9E+08	2E+09	2E+09	2E+09
B2RZ27	Protein Sh3bgrl3 OS=Rattus norvegicus GN=Sh3bgrl3 PE=4 SV=1 - [B2RZ27_RAT]	0	2E+07	0	6E+07	7E+07	8E+07
P19945	60S acidic ribosomal protein P0 OS=Rattus norvegicus GN=Rplp0 PE=1 SV=2 - [RLA0_RAT]	2E+09	9E+08	5E+08	3E+09	3E+09	3E+09
P17077	60S ribosomal protein L9 OS=Rattus norvegicus GN=Rpl9 PE=1 SV=1 - [RL9_RAT]	8E+08	4E+08	2E+08	1E+09	1E+09	1E+09
F1LPY7	Trans-2-enoyl-CoA reductase, mitochondrial OS=Rattus norvegicus GN=Mecr PE=4 SV=1 - [F1LPY7_RAT]	6E+08	4E+08	2E+08	8E+08	7E+08	8E+08
P07151	Beta-2-microglobulin OS=Rattus norvegicus GN=B2m PE=1 SV=1 - [B2MG_RAT]	0	0	6E+07	2E+08	1E+08	3E+08
P07633	Propionyl-CoA carboxylase beta chain, mitochondrial OS=Rattus norvegicus GN=Pccb PE=2 SV=1 - [PCCB_RAT]	2E+09	2E+09	9E+08	2E+09	2E+09	3E+09
F1LPK7	Plastin 3 (T-isoform), isoform CRA_a OS=Rattus norvegicus GN=Pls3 PE=4 SV=2 - [F1LPK7_RAT]	9E+08	9E+08	6E+08	1E+09	2E+09	2E+09
P14480	Fibrinogen beta chain OS=Rattus norvegicus GN=Fgb PE=1 SV=4 - [FIBB_RAT]	8E+08	5E+08	4E+08	8E+08	1E+09	1E+09
P55051	Fatty acid-binding protein, brain OS=Rattus norvegicus GN=Fabp7 PE=1 SV=2 - [FABP7_RAT]	2E+06	3E+08	1E+08	7E+08	1E+09	4E+08
D4ABR6	Annexin (Fragment) OS=Rattus norvegicus GN=Anxa6 PE=3 SV=2 - [D4ABR6_RAT]	1E+09	1E+09	9E+08	2E+09	2E+09	2E+09
D4AB01	Histidine triad nucleotide binding protein 2 (Predicted), isoform CRA_a OS=Rattus norvegicus GN=Hint2 PE=4 SV=1 - [D4AB01_RAT]	2E+09	1E+09	2E+09	4E+09	3E+09	3E+09
P62832	60S ribosomal protein L23 OS=Rattus norvegicus GN=Rpl23 PE=2 SV=1 - [RL23_RAT]	8E+08	6E+08	1E+09	2E+09	2E+09	2E+09
Q9EQ76	Dimethylaniline monooxygenase [N-oxide-forming] 3 OS=Rattus norvegicus GN=Fmo3 PE=1 SV=1 - [FMO3_RAT]	2E+09	1E+09	9E+08	3E+09	2E+09	3E+09
Q562C9	1,2-dihydroxy-3-keto-5-methylthiopentene dioxygenase OS=Rattus norvegicus GN=Adi1 PE=2 SV=1 - [MTND_RAT]	3E+08	2E+08	3E+08	6E+08	7E+08	5E+08
Q63010	Liver carboxylesterase B-1 OS=Rattus norvegicus PE=1 SV=1 - [EST5_RAT]	5E+09	8E+09	6E+09	1E+10	1E+10	1E+10
Q06647	ATP synthase subunit O, mitochondrial OS=Rattus norvegicus GN=Atp5o PE=1 SV=1 - [ATPO_RAT]	4E+09	3E+09	3E+09	8E+09	8E+09	8E+09
D3ZIC2	Protein Uroc1 OS=Rattus norvegicus GN=Uroc1 PE=3 SV=2 - [D3ZIC2_RAT]	3E+09	4E+09	3E+09	6E+09	5E+09	7E+09
P04916	Retinol-binding protein 4 OS=Rattus norvegicus GN=Rbp4 PE=1 SV=1 - [RET4_RAT]	3E+08	4E+08	8E+07	1E+09	6E+08	1E+09
F1LQ55	Non-specific lipid-transfer protein OS=Rattus norvegicus GN=Scp2 PE=4 SV=2 - [F1LQ55_RAT]	2E+09	2E+09	1E+09	4E+09	3E+09	3E+09
Q3T1K5	F-actin-capping protein subunit alpha-2 OS=Rattus norvegicus GN=Capza2 PE=1 SV=1 - [CAZA2_RAT]	4E+08	2E+08	3E+08	8E+08	6E+08	7E+08
D3ZD09	Cytochrome c oxidase subunit 6B1 OS=Rattus norvegicus GN=Cox6b1 PE=4 SV=1 - [D3ZD09_RAT]	9E+08	4E+08	6E+08	2E+09	3E+09	3E+09
M0RDC5	Acyl-CoA-binding protein (Fragment) OS=Rattus norvegicus GN=Dbi PE=4 SV=1 - [M0RDC5_RAT]	2E+09	2E+09	2E+09	5E+09	4E+09	5E+09
Q5VLR5	BWK4 OS=Rattus norvegicus GN=Erp44 PE=2 SV=1 - [Q5VLR5_RAT]	2E+09	1E+09	7E+08	2E+09	2E+09	2E+09
Q8VID1	Dehydrogenase/reductase SDR family member 4 OS=Rattus norvegicus GN=Dhrs4 PE=2 SV=2 - [DHRS4_RAT]	6E+08	4E+08	2E+08	9E+08	8E+08	9E+08
Q6NYB7	Ras-related protein Rab-1A OS=Rattus norvegicus GN=Rab1A PE=1 SV=3 - [RAB1A_RAT]	9E+08	7E+08	4E+08	1E+09	1E+09	1E+09
Q6AY18	Protein Sar1a OS=Rattus norvegicus GN=Sar1a PE=2 SV=1 - [Q6AY18_RAT]	7E+08	5E+08	4E+08	2E+09	1E+09	2E+09
Q561S0	NADH dehydrogenase [ubiquinone] 1 alpha subcomplex subunit 10, mitochondrial OS=Rattus norvegicus GN=Ndufa10 PE=1 SV=1 - [NDUAA_RAT]	9E+08	6E+08	3E+08	2E+09	2E+09	1E+09
Q63108	Carboxylesterase 1E OS=Rattus norvegicus GN=Ces1e PE=2 SV=1 - [EST1E_RAT]	3E+09	5E+09	6E+09	8E+09	9E+09	1E+10
Q7M0E3	Destrin OS=Rattus norvegicus GN=Dstn PE=1 SV=3 - [DEST_RAT]	4E+08	5E+08	2E+08	1E+09	6E+08	1E+09
Q62636	Ras-related protein Rap-1b OS=Rattus norvegicus GN=Rap1b PE=2 SV=2 - [RAP1B_RAT]	2E+08	2E+08	5E+07	5E+08	3E+08	4E+08
P85970	Actin-related protein 2/3 complex subunit 2 OS=Rattus norvegicus GN=Arpc2 PE=1 SV=1 - [ARPC2_RAT]	4E+08	3E+08	2E+08	6E+08	4E+08	5E+08
D3ZLT1	NADH dehydrogenase (Ubiquinone) 1 beta subcomplex, 7 (Predicted) OS=Rattus norvegicus GN=Ndufb7 PE=4 SV=1 - [D3ZLT1_RAT]	6E+07	7E+07	0	2E+08	3E+08	2E+08
Q80Z29	Nicotinamide phosphoribosyltransferase OS=Rattus norvegicus GN=Nampt PE=1 SV=1 - [NAMPT_RAT]	9E+08	6E+08	5E+08	1E+09	2E+09	2E+09
F1LXA0	NADH dehydrogenase (Ubiquinone) 1 alpha subcomplex, 12 (Predicted), isoform CRA_b OS=Rattus norvegicus GN=Ndufa12 PE=4 SV=2 - [F1LXA0_RAT]	3E+08	5E+08	4E+08	7E+08	1E+09	8E+08
Q5XHZ0	Heat shock protein 75 kDa, mitochondrial OS=Rattus norvegicus GN=Trap1 PE=1 SV=1 - [TRAP1_RAT]	4E+09	6E+09	5E+09	7E+09	9E+09	8E+09

B0K008	Eukaryotic translation initiation factor 1 OS=Rattus norvegicus GN=Eif1l1 PE=4 SV=1 - [B0K008_RAT]	0	5E+07	5E+07	2E+08	3E+08	3E+08
Q5XIM9	T-complex protein 1 subunit beta OS=Rattus norvegicus GN=Cct2 PE=1 SV=3 - [TCPB_RAT]	8E+08	9E+08	9E+08	2E+09	1E+09	1E+09
G3V7I0	Peroxiredoxin 3 OS=Rattus norvegicus GN=Prdx3 PE=4 SV=1 - [G3V7I0_RAT]	2E+09	2E+09	4E+08	4E+09	2E+09	4E+09
B0K010	Protein Txndc17 OS=Rattus norvegicus GN=Txndc17 PE=2 SV=1 - [B0K010_RAT]	4E+08	4E+08	7E+08	1E+09	1E+09	1E+09
B2GV15	Dihydrolipoamide branched chain transacylase E2 OS=Rattus norvegicus GN=Dbt PE=2 SV=1 - [B2GV15_RAT]	1E+09	1E+09	7E+08	2E+09	2E+09	2E+09
F1M953	Stress-70 protein, mitochondrial OS=Rattus norvegicus GN=Hspa9 PE=3 SV=1 - [F1M953_RAT]	6E+09	6E+09	4E+09	9E+09	1E+10	1E+10
P05545	Serine protease inhibitor A3K OS=Rattus norvegicus GN=Serpina3k PE=1 SV=3 - [SPA3K_RAT]	2E+09	2E+09	2E+09	2E+09	3E+09	2E+09
D4A5L9	Protein LOC679794 OS=Rattus norvegicus GN=LOC679794 PE=3 SV=1 - [D4A5L9_RAT]	1E+09	1E+09	2E+08	4E+09	2E+09	3E+09
Q8VBU2-2	Isoform 2 of Protein NDRG2 OS=Rattus norvegicus GN=Ndrg2 - [NDRG2_RAT]	1E+09	1E+09	7E+08	2E+09	2E+09	2E+09
Q8VBU2	Protein NDRG2 OS=Rattus norvegicus GN=Ndrg2 PE=1 SV=1 - [NDRG2_RAT]	1E+09	1E+09	7E+08	2E+09	2E+09	2E+09
F7FLF2	Protein LOC100360057 (Fragment) OS=Rattus norvegicus GN=LOC100363800 PE=4 SV=1 - [F7FLF2_RAT]	4E+08	5E+08	8E+08	2E+09	2E+09	1E+09
F1M8L5	Propionyl-CoA carboxylase alpha chain, mitochondrial OS=Rattus norvegicus GN=Pcca PE=4 SV=2 - [F1M8L5_RAT]	9E+08	1E+09	9E+08	2E+09	2E+09	2E+09
P24368	Peptidyl-prolyl cis-trans isomerase B OS=Rattus norvegicus GN=Ppib PE=1 SV=3 - [PPIB_RAT]	9E+08	1E+09	8E+08	3E+09	3E+09	2E+09
O35820	2'-deoxynucleoside 5'-phosphate N-hydrolase 1 OS=Rattus norvegicus GN=Dnph1 PE=1 SV=1 - [DNPH1_RAT]	0	1E+08	0	1E+08	2E+08	2E+08
P27605	Hypoxanthine-guanine phosphoribosyltransferase OS=Rattus norvegicus GN=Hprt1 PE=1 SV=1 - [HPRT_RAT]	1E+09	1E+09	6E+08	2E+09	2E+09	1E+09
Q4FZT0	Stomatin-like protein 2, mitochondrial OS=Rattus norvegicus GN=Stoml2 PE=1 SV=1 - [STML2_RAT]	4E+08	4E+08	2E+08	8E+08	9E+08	6E+08
Q5U211	Sorting nexin-3 OS=Rattus norvegicus GN=Snx3 PE=1 SV=1 - [SNX3_RAT]	7E+07	9E+07	0	3E+08	2E+08	2E+08
F1LLV6	Protein Ces1f OS=Rattus norvegicus GN=Ces1f PE=4 SV=2 - [F1LLV6_RAT]	5E+09	8E+09	6E+09	1E+10	1E+10	1E+10
P08503	Medium-chain specific acyl-CoA dehydrogenase, mitochondrial OS=Rattus norvegicus GN=Acadm PE=1 SV=1 - [ACADM_RAT]	5E+09	5E+09	3E+09	8E+09	7E+09	6E+09
P19468	Glutamate--cysteine ligase catalytic subunit OS=Rattus norvegicus GN=Gclc PE=1 SV=2 - [GSH1_RAT]	6E+08	5E+08	6E+08	8E+08	9E+08	8E+08
D3ZHB3	40S ribosomal protein S12 OS=Rattus norvegicus GN=LOC100360573 PE=3 SV=2 - [D3ZHB3_RAT]	4E+08	5E+08	7E+08	2E+09	3E+09	2E+09
D3ZLK9	ATP-dependent (S)-NAD(P)H-hydrate dehydratase OS=Rattus norvegicus GN=Carkd PE=3 SV=1 - [D3ZLK9_RAT]	4E+08	3E+08	1E+08	7E+08	5E+08	8E+08
Q5RKH2	Galactokinase 1 OS=Rattus norvegicus GN=Galk1 PE=2 SV=1 - [Q5RKH2_RAT]	5E+08	7E+08	3E+08	1E+09	2E+09	8E+08
Q9Z311	Trans-2-enoyl-CoA reductase, mitochondrial OS=Rattus norvegicus GN=Mecr PE=2 SV=1 - [MECR_RAT]	6E+08	4E+08	2E+08	8E+08	7E+08	8E+08
Q5XIH7	Prohibitin-2 OS=Rattus norvegicus GN=Phb2 PE=1 SV=1 - [PHB2_RAT]	2E+09	1E+09	1E+09	3E+09	3E+09	3E+09
P82995	Heat shock protein HSP 90-alpha OS=Rattus norvegicus GN=Hsp90aa1 PE=1 SV=3 - [HS90A_RAT]	7E+09	1E+10	8E+09	1E+10	1E+10	1E+10
P61589	Transforming protein RhoA OS=Rattus norvegicus GN=Rhoa PE=1 SV=1 - [RHOA_RAT]	3E+08	3E+08	6E+07	6E+08	5E+08	8E+08
B2RYP0	Protein Rhoc OS=Rattus norvegicus GN=Rhoc PE=2 SV=1 - [B2RYP0_RAT]	2E+08	2E+08	1E+08	6E+08	4E+08	8E+08
Q6P791	Ragulator complex protein LAMTOR1 OS=Rattus norvegicus GN=Lamtor1 PE=1 SV=1 - [LTOR1_RAT]	2E+07	4E+07	3E+07	1E+08	1E+08	1E+08
P00388	NADPH--cytochrome P450 reductase OS=Rattus norvegicus GN=Por PE=1 SV=3 - [NCPR_RAT]	1E+09	2E+09	2E+09	3E+09	3E+09	3E+09
P97852	Peroxisomal multifunctional enzyme type 2 OS=Rattus norvegicus GN=Hsd17b4 PE=1 SV=3 - [DHB4_RAT]	3E+09	3E+09	2E+09	4E+09	4E+09	4E+09
P16232-2	Isoform 11-HSD1B of Corticosteroid 11-beta-dehydrogenase isozyme 1 OS=Rattus norvegicus GN=Hsd11b1 - [DHI1_RAT]	1E+09	2E+09	9E+08	3E+09	2E+09	3E+09
Q63569	26S protease regulatory subunit 6A OS=Rattus norvegicus GN=Psmc3 PE=2 SV=1 - [PRS6A_RAT]	5E+08	4E+08	2E+08	7E+08	9E+08	7E+08
Q68FR6	Elongation factor 1-gamma OS=Rattus norvegicus GN=Eef1g PE=1 SV=3 - [EF1G_RAT]	2E+09	1E+09	1E+09	2E+09	3E+09	2E+09
P62914	60S ribosomal protein L11 OS=Rattus norvegicus GN=Rpl11 PE=1 SV=2 - [RL11_RAT]	8E+08	6E+08	8E+08	2E+09	3E+09	2E+09
P17220	Proteasome subunit alpha type-2 OS=Rattus norvegicus GN=Psma2 PE=1 SV=3 - [PSA2_RAT]	6E+08	9E+08	1E+08	2E+09	1E+09	1E+09
D4A7D7	Hexose-6-phosphate dehydrogenase (Glucose 1-dehydrogenase) (Predicted), isoform CRA_b OS=Rattus norvegicus GN=H6pd PE=4 SV=1 - [D4A7D7_RAT]	7E+08	1E+09	8E+08	2E+09	2E+09	2E+09
B0BNA5	Coactosin-like protein OS=Rattus norvegicus GN=Cotl1 PE=1 SV=1 - [COTL1_RAT]	1E+08	1E+08	2E+08	3E+08	5E+08	3E+08
Q9WVJ4	Synaptojanin-2-binding protein OS=Rattus norvegicus GN=Synj2bp PE=1 SV=2 - [SYJ2B_RAT]	7E+07	8E+07	4E+07	3E+08	3E+08	3E+08
P11915	Non-specific lipid-transfer protein OS=Rattus norvegicus GN=Scp2 PE=1 SV=3 - [NLTP_RAT]	2E+09	2E+09	1E+09	4E+09	3E+09	3E+09
O89035	Mitochondrial dicarboxylate carrier OS=Rattus norvegicus GN=Slc25a10 PE=2 SV=1 - [O89035_RAT]	5E+08	4E+08	3E+08	8E+08	8E+08	1E+09
M0R9D0	Protein LOC100909983 OS=Rattus norvegicus GN=Anp32a PE=4 SV=1 - [M0R9D0_RAT]	3E+08	3E+08	6E+07	8E+08	8E+08	7E+08
P05065	Fructose-bisphosphate aldolase A OS=Rattus norvegicus GN=Aldoa PE=1 SV=2 - [ALDOA_RAT]	3E+08	2E+08	1E+08	5E+08	5E+08	5E+08
F7FIH7	Protein LOC100909412 OS=Rattus norvegicus GN=LOC688457 PE=3 SV=1 - [F7FIH7_RAT]	2E+09	3E+09	3E+09	1E+10	6E+09	6E+09
F1M8E9	Putative lysozyme C-2 OS=Rattus norvegicus GN=Lyz2 PE=3 SV=1 - [F1M8E9_RAT]	7E+07	1E+08	0	3E+08	2E+08	2E+08
D3ZB30	Polypyrimidine tract binding protein 1, isoform CRA_c OS=Rattus norvegicus GN=Ptbp1 PE=4 SV=1 - [D3ZB30_RAT]	6E+08	4E+08	3E+08	9E+08	8E+08	8E+08
P0C0A1	Vacuolar protein-sorting-associated protein 25 OS=Rattus norvegicus GN=Vps25 PE=1 SV=1 - [VPS25_RAT]	5E+07	6E+07	7E+07	1E+08	1E+08	1E+08
P09606	Glutamine synthetase OS=Rattus norvegicus GN=Glul PE=1 SV=3 - [GLNA_RAT]	3E+09	3E+09	1E+09	7E+09	6E+09	5E+09
Q9JHW0	Proteasome subunit beta type-7 OS=Rattus norvegicus GN=Psmb7 PE=1 SV=1 - [PSB7_RAT]	4E+07	1E+08	0	2E+08	2E+08	2E+08
P26284	Pyruvate dehydrogenase E1 component subunit alpha, somatic form, mitochondrial OS=Rattus norvegicus GN=Pdha1 PE=1 SV=2 - [ODPA_RAT]	2E+09	2E+09	8E+08	3E+09	4E+09	3E+09
P40307	Proteasome subunit beta type-2 OS=Rattus norvegicus GN=Psmb2 PE=1 SV=1 - [PSB2_RAT]	5E+08	7E+08	7E+08	2E+09	1E+09	2E+09
G3V9E4	Acylamino-acid-releasing enzyme OS=Rattus norvegicus GN=Apeh PE=4 SV=1 - [G3V9E4_RAT]	5E+08	6E+08	4E+08	6E+08	7E+08	7E+08
P04639	Apolipoprotein A-I OS=Rattus norvegicus GN=Apoa1 PE=1 SV=2 - [APOA1_RAT]	8E+08	1E+09	3E+08	2E+09	2E+09	2E+09
P25235	Dolichyl-diphosphooligosaccharide--protein glycosyltransferase subunit 2 OS=Rattus norvegicus GN=Rpn2 PE=2 SV=2 - [RPN2_RAT]	3E+08	2E+08	1E+08	4E+08	6E+08	6E+08
Q10758	Keratin, type II cytoskeletal 8 OS=Rattus norvegicus GN=Krt8 PE=1 SV=3 - [K2C8_RAT]	9E+08	1E+09	6E+08	5E+09	4E+09	3E+09
D3ZIF6	Protein LOC100360095 OS=Rattus norvegicus GN=LOC100360095 PE=4 SV=1 - [D3ZIF6_RAT]	0	6E+07	1E+07	4E+08	1E+08	4E+08

Q58FK9	Kynurenine--oxoglutarate transaminase 3 OS=Rattus norvegicus GN=Ccbl2 PE=2 SV=1 - [KAT3_RAT]	4E+09	3E+09	2E+09	8E+09	6E+09	7E+09
G3V9N2	Peroxisomal membrane protein 2 OS=Rattus norvegicus GN=Pxmp2 PE=4 SV=1 - [G3V9N2_RAT]	9E+07	4E+07	4E+07	3E+08	2E+08	2E+08
G3V6W6	Protein Psmc6 OS=Rattus norvegicus GN=Psmc6 PE=3 SV=1 - [G3V6W6_RAT]	6E+08	7E+08	3E+08	1E+09	1E+09	8E+08
Q5BJN1	Protein Stard10 OS=Rattus norvegicus GN=Stard10 PE=2 SV=1 - [Q5BJN1_RAT]	4E+09	3E+09	2E+09	8E+09	6E+09	6E+09
Q8CG45	Aflatoxin B1 aldehyde reductase member 2 OS=Rattus norvegicus GN=Akr7a2 PE=1 SV=2 - [ARK72_RAT]	3E+09	3E+09	2E+09	5E+09	4E+09	5E+09
P05182	Cytochrome P450 2E1 OS=Rattus norvegicus GN=Cyp2e1 PE=1 SV=4 - [CP2E1_RAT]	2E+09	1E+09	7E+08	3E+09	4E+09	3E+09
Q7TP48	Adipocyte plasma membrane-associated protein OS=Rattus norvegicus GN=Apmap PE=2 SV=2 - [APMAP_RAT]	5E+08	3E+08	2E+08	8E+08	7E+08	6E+08
F1M0Q9	Protein Pm20d1 (Fragment) OS=Rattus norvegicus GN=Pm20d1 PE=4 SV=2 - [F1M0Q9_RAT]	4E+08	4E+08	2E+08	7E+08	6E+08	7E+08
Q5XIE6	3-hydroxyisobutyryl-CoA hydrolase, mitochondrial OS=Rattus norvegicus GN=Hibch PE=1 SV=2 - [HIBCH_RAT]	5E+08	4E+08	2E+08	7E+08	7E+08	8E+08
B5DFC3	Protein Sec23a OS=Rattus norvegicus GN=Sec23a PE=2 SV=1 - [B5DFC3_RAT]	6E+08	8E+08	7E+08	1E+09	2E+09	1E+09
Q6PDU7	ATP synthase subunit g, mitochondrial OS=Rattus norvegicus GN=Atp5l PE=1 SV=2 - [ATP5L_RAT]	3E+08	5E+08	3E+08	7E+08	1E+09	1E+09
B0BMW0	RAB14, member RAS oncogene family OS=Rattus norvegicus GN=Rab14 PE=2 SV=1 - [B0BMW0_RAT]	6E+08	4E+08	4E+08	8E+08	7E+08	8E+08
P24473	Glutathione S-transferase kappa 1 OS=Rattus norvegicus GN=Gstk1 PE=1 SV=3 - [GSTK1_RAT]	1E+09	1E+09	1E+09	4E+09	2E+09	3E+09
P20788	Cytochrome b-c1 complex subunit Rieske, mitochondrial OS=Rattus norvegicus GN=Uqcrfs1 PE=1 SV=2 - [UCRI_RAT]	4E+08	2E+08	2E+08	9E+08	5E+08	9E+08
Q7TQ16	Cytochrome b-c1 complex subunit 8 OS=Rattus norvegicus GN=Uqcrq PE=3 SV=1 - [QCR8_RAT]	8E+08	6E+08	9E+08	1E+09	2E+09	1E+09
P62198	26S protease regulatory subunit 8 OS=Rattus norvegicus GN=Psmc5 PE=1 SV=1 - [PRS8_RAT]	4E+08	3E+08	2E+08	5E+08	5E+08	5E+08
D4A7G9	Protein RGD1564804 OS=Rattus norvegicus GN=RGD1564804 PE=4 SV=1 - [D4A7G9_RAT]	5E+07	1E+08	1E+08	3E+08	3E+08	2E+08
P09118	Uricase OS=Rattus norvegicus GN=Uox PE=1 SV=3 - [URIC_RAT]	1E+09	5E+08	2E+08	2E+09	1E+09	2E+09
P09139-2	Isoform Peroxisomal of Serine--pyruvate aminotransferase, mitochondrial OS=Rattus norvegicus GN=Agxt - [SPYA_RAT]	6E+08	7E+08	2E+08	2E+09	1E+09	1E+09
F1LRV4	Heat shock 70 kDa protein 4 OS=Rattus norvegicus GN=Hspa4 PE=3 SV=1 - [F1LRV4_RAT]	8E+08	1E+09	8E+08	1E+09	1E+09	1E+09
P13086	Succinyl-CoA ligase [ADP/GDP-forming] subunit alpha, mitochondrial OS=Rattus norvegicus GN=Suclg1 PE=2 SV=2 - [SUCA_RAT]	5E+09	3E+09	2E+09	7E+09	6E+09	8E+09
D3ZNQ6	Protein Ube2m OS=Rattus norvegicus GN=Ube2m PE=3 SV=1 - [D3ZNQ6_RAT]	9E+07	1E+08	1E+08	2E+08	3E+08	3E+08
P11240	Cytochrome c oxidase subunit 5A, mitochondrial OS=Rattus norvegicus GN=Cox5a PE=1 SV=1 - [COX5A_RAT]	5E+08	6E+08	1E+08	2E+09	2E+09	2E+09
Q5BK81	Prostaglandin reductase 2 OS=Rattus norvegicus GN=Ptgr2 PE=2 SV=2 - [PTGR2_RAT]	5E+08	4E+08	2E+08	1E+09	1E+09	8E+08
Q6AYU5	Poly(RC) binding protein 2 OS=Rattus norvegicus GN=Pcbp2 PE=2 SV=1 - [Q6AYU5_RAT]	5E+08	4E+08	2E+08	9E+08	8E+08	7E+08
D4A133	Protein Atp6v1a OS=Rattus norvegicus GN=Atp6v1a PE=3 SV=1 - [D4A133_RAT]	2E+08	2E+08	2E+08	4E+08	6E+08	4E+08
P62836	Ras-related protein Rap-1A OS=Rattus norvegicus GN=Rap1a PE=1 SV=1 - [RAP1A_RAT]	2E+08	2E+08	5E+07	5E+08	3E+08	4E+08
P55159	Serum paraoxonase/arylesterase 1 OS=Rattus norvegicus GN=Pon1 PE=1 SV=3 - [PON1_RAT]	3E+09	3E+09	1E+09	8E+09	6E+09	5E+09
Q68FQ0	T-complex protein 1 subunit epsilon OS=Rattus norvegicus GN=Cct5 PE=1 SV=1 - [TCPE_RAT]	5E+08	5E+08	4E+08	7E+08	9E+08	9E+08
D4A4L5	Protein Isca2 OS=Rattus norvegicus GN=Isca2 PE=4 SV=1 - [D4A4L5_RAT]	9E+07	1E+08	1E+08	4E+08	4E+08	3E+08
Q920D2	Dihydrofolate reductase OS=Rattus norvegicus GN=Dhfr PE=2 SV=3 - [DYR_RAT]	5E+08	8E+08	5E+08	2E+09	1E+09	1E+09
O35078	D-amino-acid oxidase OS=Rattus norvegicus GN=Dao PE=2 SV=1 - [OXDA_RAT]	2E+08	2E+08	2E+08	4E+08	4E+08	3E+08
Q07071	Glucokinase regulatory protein OS=Rattus norvegicus GN=Gckr PE=1 SV=3 - [GCKR_RAT]	1E+09	1E+09	1E+09	2E+09	2E+09	2E+09
P28492-2	Isoform 2 of Glutaminase liver isoform, mitochondrial OS=Rattus norvegicus GN=Gls2 - [GLSL_RAT]	1E+09	1E+09	6E+08	3E+09	2E+09	2E+09
M0R9I6	Aminomethyltransferase OS=Rattus norvegicus GN=Amt PE=3 SV=1 - [M0R9I6_RAT]	9E+08	9E+08	7E+08	2E+09	2E+09	1E+09
P28480	T-complex protein 1 subunit alpha OS=Rattus norvegicus GN=Tcp1 PE=1 SV=1 - [TCPA_RAT]	3E+08	6E+08	4E+08	7E+08	7E+08	9E+08
Q641Y2	NADH dehydrogenase [ubiquinone] iron-sulfur protein 2, mitochondrial OS=Rattus norvegicus GN=Ndufs2 PE=1 SV=1 - [NDUS2_RAT]	6E+08	4E+08	2E+08	8E+08	1E+09	8E+08
P97532	3-mercaptopyruvate sulfurtransferase OS=Rattus norvegicus GN=Mpst PE=1 SV=3 - [THTM_RAT]	4E+09	4E+09	2E+09	7E+09	6E+09	6E+09
Q32PX2	Aminoacyl tRNA synthase complex-interacting multifunctional protein 2 OS=Rattus norvegicus GN=Aimp2 PE=2 SV=1 - [AIMP2_RAT]	2E+08	1E+08	4E+07	3E+08	2E+08	3E+08
P70584	Short/branched chain specific acyl-CoA dehydrogenase, mitochondrial OS=Rattus norvegicus GN=Acadsb PE=1 SV=1 - [ACDSB_RAT]	1E+09	1E+09	7E+08	1E+09	1E+09	1E+09
Q641Y0	Dolichyl-diphosphooligosaccharide--protein glycosyltransferase 48 kDa subunit OS=Rattus norvegicus GN=Ddost PE=2 SV=1 - [OST48_RAT]	4E+08	3E+08	2E+08	7E+08	7E+08	8E+08
D3ZFA8	Protein LOC100362366 OS=Rattus norvegicus GN=LOC100364909 PE=3 SV=1 - [D3ZFA8_RAT]	5E+08	5E+08	4E+08	2E+09	2E+09	2E+09
D3Z8D7	Protein LOC100361854 OS=Rattus norvegicus GN=LOC100361854 PE=4 SV=1 - [D3Z8D7_RAT]	6E+08	3E+08	1E+08	8E+08	1E+09	9E+08
Q920F5-2	Isoform Cytoplasmic+peroxisomal of Malonyl-CoA decarboxylase, mitochondrial OS=Rattus norvegicus GN=Mlycd - [DCMC_RAT]	7E+08	4E+08	3E+08	8E+08	9E+08	1E+09
D4ACV3	Histone H2A OS=Rattus norvegicus GN=Hist2h2ac PE=3 SV=1 - [D4ACV3_RAT]	1E+07	2E+08	2E+07	2E+08	3E+08	2E+08
Q9JLA3	UDP-glucose:glycoprotein glucosyltransferase 1 OS=Rattus norvegicus GN=Uggt1 PE=1 SV=2 - [UGGG1_RAT]	4E+08	7E+08	6E+08	9E+08	1E+09	1E+09
B2RYW3	NADH dehydrogenase (Ubiquinone) 1 beta subcomplex, 9 OS=Rattus norvegicus GN=Ndufb9 PE=2 SV=1 - [B2RYW3_RAT]	1E+08	5E+07	1E+08	2E+08	2E+08	3E+08
D3ZWV2	Glyceraldehyde-3-phosphate dehydrogenase OS=Rattus norvegicus PE=3 SV=2 - [D3ZWV2_RAT]	1E+10	8E+09	5E+09	2E+10	1E+10	2E+10
F1LW74	Protein Iqgap2 OS=Rattus norvegicus GN=Iqgap2 PE=4 SV=2 - [F1LW74_RAT]	1E+09	1E+09	7E+08	1E+09	2E+09	2E+09
Q6P502	T-complex protein 1 subunit gamma OS=Rattus norvegicus GN=Cct3 PE=1 SV=1 - [TCPG_RAT]	8E+08	1E+09	8E+08	2E+09	2E+09	2E+09
D3ZFJ6	Lactamase, beta (Predicted) OS=Rattus norvegicus GN=Lactb PE=4 SV=1 - [D3ZFJ6_RAT]	5E+08	4E+08	5E+08	9E+08	8E+08	8E+08
Q5XHY5	Threonine--tRNA ligase, cytoplasmic OS=Rattus norvegicus GN=Tars PE=2 SV=1 - [SYTC_RAT]	1E+09	1E+09	8E+08	1E+09	2E+09	1E+09
Q3MHS9	Chaperonin containing Tcp1, subunit 6A (Zeta 1) OS=Rattus norvegicus GN=Cct6a PE=2 SV=1 - [Q3MHS9_RAT]	5E+08	8E+08	5E+08	9E+08	1E+09	1E+09
Q5RJR8	Leucine-rich repeat-containing protein 59 OS=Rattus norvegicus GN=Lrrc59 PE=1 SV=1 - [LRC59_RAT]	1E+09	6E+08	3E+08	2E+09	2E+09	2E+09
P14882	Propionyl-CoA carboxylase alpha chain, mitochondrial OS=Rattus norvegicus GN=Pcca PE=1 SV=3 - [PCCA_RAT]	9E+08	1E+09	9E+08	2E+09	2E+09	2E+09
F1LQQ8	Beta-glucuronidase OS=Rattus norvegicus GN=Gusb PE=4 SV=2 - [F1LQQ8_RAT]	4E+08	2E+08	5E+08	6E+08	9E+08	8E+08

G3V9N8	AP-1 complex subunit beta-1 OS=Rattus norvegicus GN=Ap1b1 PE=4 SV=2 - [G3V9N8_RAT]	3E+08	5E+08	4E+08	8E+08	9E+08	1E+09
B5DEL8	NADH dehydrogenase (Ubiquinone) Fe-S protein 5 OS=Rattus norvegicus GN=LOC100361505 PE=4 SV=1 - [B5DEL8_RAT]	8E+07	6E+07	0	4E+08	4E+08	5E+08
Q4KM73	UMP-CMP kinase OS=Rattus norvegicus GN=Cmpk1 PE=1 SV=2 - [KCY_RAT]	9E+08	8E+08	8E+08	2E+09	2E+09	2E+09
P47942	Dihydropyrimidinase-related protein 2 OS=Rattus norvegicus GN=Dpysl2 PE=1 SV=1 - [DPYL2_RAT]	4E+08	6E+08	3E+08	7E+08	9E+08	8E+08
P02650	Apolipoprotein E OS=Rattus norvegicus GN=Apoe PE=1 SV=2 - [APOE_RAT]	1E+09	2E+09	1E+09	2E+09	2E+09	2E+09
F1LP30	Methylcrotonoyl-CoA carboxylase subunit alpha, mitochondrial OS=Rattus norvegicus GN=Mccc1 PE=3 SV=1 - [F1LP30_RAT]	7E+08	9E+08	5E+08	1E+09	1E+09	2E+09
D3Z9G9	Protein LOC690772 (Fragment) OS=Rattus norvegicus GN=RGD1560017 PE=3 SV=2 - [D3Z9G9_RAT]	3E+08	3E+08	1E+08	9E+08	9E+08	8E+08
Q4V8J2	Ubiquitin/ISG15-conjugating enzyme E2 L6 OS=Rattus norvegicus GN=Ube2l6 PE=2 SV=1 - [UB2L6_RAT]	3E+06	0	6E+06	4E+07	3E+07	6E+07
M0RDJ4	Glia maturation factor beta OS=Rattus norvegicus GN=Gmfb PE=4 SV=1 - [M0RDJ4_RAT]	2E+08	2E+08	3E+08	4E+08	5E+08	4E+08
P63159	High mobility group protein B1 OS=Rattus norvegicus GN=Hmgb1 PE=1 SV=2 - [HMGB1_RAT]	1E+08	4E+07	6E+07	2E+08	4E+08	4E+08
Q6AYK6	Calcyclin-binding protein OS=Rattus norvegicus GN=Cacybp PE=1 SV=1 - [CYBP_RAT]	2E+08	2E+08	1E+08	4E+08	3E+08	3E+08
Q63041	Alpha-1-macroglobulin OS=Rattus norvegicus GN=A1m PE=1 SV=1 - [A1M_RAT]	1E+09	2E+09	1E+09	2E+09	3E+09	2E+09
P07953	6-phosphofructo-2-kinase/fructose-2,6-bisphosphatase 1 OS=Rattus norvegicus GN=Pfkfb1 PE=1 SV=3 - [F261_RAT]	4E+08	2E+08	2E+08	6E+08	5E+08	6E+08
Q4FZU0	Acid phosphatase 6, lysophosphatidic OS=Rattus norvegicus GN=Acp6 PE=2 SV=1 - [Q4FZU0_RAT]	5E+08	4E+08	2E+08	9E+08	8E+08	6E+08
P35435	ATP synthase subunit gamma, mitochondrial OS=Rattus norvegicus GN=Atp5c1 PE=1 SV=2 - [ATPG_RAT]	1E+09	8E+08	8E+08	2E+09	2E+09	2E+09
P05183	Cytochrome P450 3A2 OS=Rattus norvegicus GN=Cyp3a2 PE=1 SV=2 - [CP3A2_RAT]	2E+09	9E+08	9E+08	3E+09	4E+09	3E+09
P08644-2	Isoform 2B of GTPase KRas OS=Rattus norvegicus GN=Kras - [RASK_RAT]	1E+07	6E+07	6E+07	2E+08	2E+08	2E+08
Q5BK63	NADH dehydrogenase [ubiquinone] 1 alpha subcomplex subunit 9, mitochondrial OS=Rattus norvegicus GN=Ndufa9 PE=1 SV=2 - [NDUA9_RAT]	5E+08	3E+08	3E+08	8E+08	5E+08	7E+08
P0C5H9	Mesencephalic astrocyte-derived neurotrophic factor OS=Rattus norvegicus GN=Manf PE=1 SV=1 - [MANF_RAT]	3E+08	6E+08	3E+08	1E+09	1E+09	1E+09
F1LR13	Atlastin-3 OS=Rattus norvegicus GN=Atl3 PE=4 SV=2 - [F1LR13_RAT]	1E+08	3E+08	1E+08	3E+08	3E+08	4E+08
P01041	Cystatin-B OS=Rattus norvegicus GN=Cstb PE=1 SV=1 - [CYTB_RAT]	3E+08	2E+08	2E+08	8E+08	6E+08	7E+08
P85972	Vinculin OS=Rattus norvegicus GN=Vcl PE=1 SV=1 - [VINC_RAT]	6E+08	9E+08	7E+08	9E+08	1E+09	1E+09
Q3KRF2	High density lipoprotein binding protein (Vigilin) OS=Rattus norvegicus GN=Hdlbp PE=2 SV=1 - [Q3KRF2_RAT]	1E+09	1E+09	8E+08	2E+09	2E+09	2E+09
G3V6H0	Protein LOC100363782 OS=Rattus norvegicus GN=LOC100363782 PE=3 SV=1 - [G3V6H0_RAT]	7E+08	5E+08	4E+08	1E+09	9E+08	1E+09
B1WC26	N-acetylneuraminic acid synthase OS=Rattus norvegicus GN=Nans PE=2 SV=1 - [B1WC26_RAT]	8E+08	5E+08	3E+08	1E+09	1E+09	1E+09
A0JN30	Canopy 2 homolog (Zebrafish) OS=Rattus norvegicus GN=Cnpy2 PE=2 SV=1 - [A0JN30_RAT]	4E+08	4E+08	1E+09	1E+09	2E+09	1E+09
B0BN93	26S proteasome non-ATPase regulatory subunit 13 OS=Rattus norvegicus GN=Psmd13 PE=1 SV=1 - [PSD13_RAT]	2E+08	2E+08	1E+08	5E+08	4E+08	3E+08
D3ZE15	Protein LOC100911483 OS=Rattus norvegicus GN=LOC100911483 PE=4 SV=1 - [D3ZE15_RAT]	3E+08	2E+08	1E+08	7E+08	7E+08	7E+08
Q497C3	UPF0585 protein C16orf13 homolog OS=Rattus norvegicus PE=2 SV=2 - [CP013_RAT]	7E+07	1E+08	0	2E+08	2E+08	3E+08
M0R6J9	Heterogeneous nuclear ribonucleoproteins A2/B1 OS=Rattus norvegicus GN=Hnrnpa2b1 PE=4 SV=1 - [M0R6J9_RAT]	9E+08	4E+08	2E+08	2E+09	1E+09	2E+09
P97615	Thioredoxin, mitochondrial OS=Rattus norvegicus GN=Txn2 PE=2 SV=1 - [THIOM_RAT]	3E+07	2E+07	0	2E+08	2E+08	3E+08
Q9JM53	Apoptosis-inducing factor 1, mitochondrial OS=Rattus norvegicus GN=Aifm1 PE=1 SV=1 - [AIFM1_RAT]	2E+09	1E+09	1E+09	2E+09	3E+09	2E+09
Q09073	ADP/ATP translocase 2 OS=Rattus norvegicus GN=Slc25a5 PE=1 SV=3 - [ADT2_RAT]	2E+09	2E+09	1E+09	3E+09	2E+09	4E+09
Q5XI34	Protein Ppp2r1a OS=Rattus norvegicus GN=Ppp2r1a PE=2 SV=1 - [Q5XI34_RAT]	9E+08	1E+09	7E+08	2E+09	2E+09	2E+09
Q6PEC4	S-phase kinase-associated protein 1 OS=Rattus norvegicus GN=Skp1 PE=2 SV=3 - [SKP1_RAT]	2E+07	2E+07	1E+08	2E+08	1E+08	1E+08
D4A197	Methylmalonyl CoA epimerase (Predicted), isoform CRA_d OS=Rattus norvegicus GN=Mcee PE=4 SV=1 - [D4A197_RAT]	2E+08	4E+08	4E+08	2E+09	8E+08	1E+09
Q68FT9	Selenocysteine lyase OS=Rattus norvegicus GN=Scly PE=1 SV=1 - [SCLY_RAT]	7E+08	6E+08	4E+08	8E+08	9E+08	9E+08
F1M891	Uncharacterized protein (Fragment) OS=Rattus norvegicus GN=Dpyd PE=4 SV=2 - [F1M891_RAT]	4E+08	7E+08	4E+08	1E+09	1E+09	2E+09
Q64536	[Pyruvate dehydrogenase (acetyl-transferring)] kinase isozyme 2, mitochondrial OS=Rattus norvegicus GN=Pdk2 PE=1 SV=1 - [PDK2_RAT]	1E+08	3E+08	1E+08	5E+08	5E+08	3E+08
Q4FZT8	SPRY domain-containing protein 4 OS=Rattus norvegicus GN=Spryd4 PE=2 SV=1 - [SPRY4_RAT]	4E+07	8E+07	3E+07	2E+08	2E+08	2E+08
P10867	L-gulonolactone oxidase OS=Rattus norvegicus GN=Gulo PE=1 SV=3 - [GGLO_RAT]	7E+08	9E+08	3E+08	2E+09	1E+09	2E+09
Q8CFN2	Cell division control protein 42 homolog OS=Rattus norvegicus GN=Cdc42 PE=1 SV=2 - [CDC42_RAT]	2E+08	2E+08	1E+08	7E+08	6E+08	7E+08
P18484	AP-2 complex subunit alpha-2 OS=Rattus norvegicus GN=Ap2a2 PE=1 SV=3 - [AP2A2_RAT]	2E+08	4E+08	3E+08	4E+08	5E+08	6E+08
D4A305	Coiled-coil domain containing 58 (Predicted), isoform CRA_c OS=Rattus norvegicus GN=Ccdc58 PE=1 SV=1 - [D4A305_RAT]	3E+08	2E+08	3E+08	8E+08	8E+08	5E+08
P19511	ATP synthase F(0) complex subunit B1, mitochondrial OS=Rattus norvegicus GN=Atp5f1 PE=1 SV=1 - [AT5F1_RAT]	1E+09	9E+08	4E+08	2E+09	2E+09	2E+09
P40329	Arginine--tRNA ligase, cytoplasmic OS=Rattus norvegicus GN=Rars PE=1 SV=2 - [SYRC_RAT]	3E+08	4E+08	3E+08	6E+08	7E+08	7E+08
E9PT65	Protein Rdx OS=Rattus norvegicus GN=Rdx PE=4 SV=1 - [E9PT65_RAT]	1E+09	1E+09	9E+08	2E+09	2E+09	2E+09
P62250	40S ribosomal protein S16 OS=Rattus norvegicus GN=Rps16 PE=1 SV=2 - [RS16_RAT]	6E+08	5E+08	5E+08	2E+09	2E+09	2E+09
P97576	GrpE protein homolog 1, mitochondrial OS=Rattus norvegicus GN=Grpel1 PE=1 SV=2 - [GRPE1_RAT]	2E+08	3E+08	2E+08	7E+08	5E+08	7E+08
Q4KM74	Vesicle-trafficking protein SEC22b OS=Rattus norvegicus GN=Sec22b PE=1 SV=3 - [SC22B_RAT]	7E+07	1E+08	0	2E+08	1E+08	2E+08
Q64602	Kynurenine/alpha-aminoadipate aminotransferase, mitochondrial OS=Rattus norvegicus GN=Aadat PE=1 SV=1 - [AADAT_RAT]	3E+09	3E+09	2E+09	5E+09	4E+09	4E+09
P29419	ATP synthase subunit e, mitochondrial OS=Rattus norvegicus GN=Atp5i PE=1 SV=3 - [ATP5I_RAT]	6E+08	1E+09	9E+08	3E+09	5E+09	3E+09
F1LP60	Moesin (Fragment) OS=Rattus norvegicus GN=Msn PE=4 SV=1 - [F1LP60_RAT]	1E+09	2E+09	1E+09	2E+09	3E+09	2E+09
Q4G069	Regulator of microtubule dynamics protein 1 OS=Rattus norvegicus GN=Rmdn1 PE=2 SV=1 - [RMD1_RAT]	1E+08	1E+08	2E+07	4E+08	2E+08	3E+08
Q3B7D0	Oxygen-dependent coproporphyrinogen-III oxidase, mitochondrial OS=Rattus norvegicus GN=Cpox PE=2 SV=1 - [HEM6_RAT]	5E+08	4E+08	3E+08	8E+08	7E+08	9E+08
B0BN81	40S ribosomal protein S5 OS=Rattus norvegicus GN=Rps5 PE=2 SV=1 - [B0BN81_RAT]	1E+09	8E+08	5E+08	2E+09	1E+09	3E+09

D3ZZR5	Protein Snrpa1 OS=Rattus norvegicus GN=Snrpa1 PE=4 SV=1 - [D3ZZR5_RAT]	2E+07	2E+07	0	8E+07	5E+07	5E+07
Q4G064	2-methoxy-6-polyprenyl-1,4-benzoquinol methylase, mitochondrial OS=Rattus norvegicus GN=Coq5 PE=2 SV=1 - [COQ5_RAT]	7E+07	1E+08	3E+07	1E+08	2E+08	1E+08
G3V9L3	Protein LOC100365414 OS=Rattus norvegicus GN=Dusp3 PE=4 SV=2 - [G3V9L3_RAT]	9E+07	9E+07	1E+08	2E+08	2E+08	2E+08
Q6PCU8	NADH dehydrogenase [ubiquinone] flavoprotein 3, mitochondrial OS=Rattus norvegicus GN=Ndufv3 PE=3 SV=1 - [NDUV3_RAT]	0	2E+07	4E+07	7E+07	1E+08	8E+07
D4ACB8	Chaperonin subunit 8 (Theta) (Predicted), isoform CRA_a OS=Rattus norvegicus GN=Cct8 PE=3 SV=1 - [D4ACB8_RAT]	7E+08	1E+09	7E+08	1E+09	1E+09	1E+09
Q9WVJ6	Protein Tgm2 OS=Rattus norvegicus GN=Tgm2 PE=2 SV=1 - [Q9WVJ6_RAT]	7E+08	4E+08	7E+08	1E+09	1E+09	8E+08
D4A253	Protein LOC100361547 (Fragment) OS=Rattus norvegicus GN=LOC100361547 PE=3 SV=2 - [D4A253_RAT]	3E+09	2E+09	1E+09	3E+09	3E+09	4E+09
P61980	Heterogeneous nuclear ribonucleoprotein K OS=Rattus norvegicus GN=Hnrnpk PE=1 SV=1 - [HNRPK_RAT]	1E+09	9E+08	8E+08	2E+09	2E+09	2E+09
P02706	Asialoglycoprotein receptor 1 OS=Rattus norvegicus GN=Asgr1 PE=1 SV=2 - [ASGR1_RAT]	5E+08	8E+08	4E+08	1E+09	1E+09	1E+09
Q5XIA5	Coenzyme A synthase OS=Rattus norvegicus GN=Coasy PE=2 SV=1 - [Q5XIA5_RAT]	4E+08	4E+08	4E+08	8E+08	9E+08	9E+08
P85834	Elongation factor Tu, mitochondrial OS=Rattus norvegicus GN=Tufm PE=1 SV=1 - [EFTU_RAT]	2E+09	1E+09	7E+08	3E+09	3E+09	2E+09
P63036	DnaJ homolog subfamily A member 1 OS=Rattus norvegicus GN=Dnaja1 PE=2 SV=1 - [DNJA1_RAT]	4E+08	3E+08	2E+08	7E+08	7E+08	5E+08
F1MAR6	Protein Prodh OS=Rattus norvegicus GN=Prodh PE=4 SV=2 - [F1MAR6_RAT]	4E+08	6E+08	3E+08	7E+08	9E+08	7E+08
P19225	Cytochrome P450 2C70 OS=Rattus norvegicus GN=Cyp2c70 PE=2 SV=1 - [CP270_RAT]	1E+09	7E+08	6E+08	2E+09	2E+09	2E+09
D4ABS2	Dynamin-1-like protein (Fragment) OS=Rattus norvegicus GN=Dnm1l PE=3 SV=2 - [D4ABS2_RAT]	2E+08	9E+07	1E+08	4E+08	3E+08	4E+08
P97536	Cullin-associated NEDD8-dissociated protein 1 OS=Rattus norvegicus GN=Cand1 PE=1 SV=1 - [CAND1_RAT]	6E+08	7E+08	6E+08	1E+09	1E+09	1E+09
Q6P7A7	Dolichyl-diphosphooligosaccharide--protein glycosyltransferase subunit 1 OS=Rattus norvegicus GN=Rpn1 PE=2 SV=1 - [Q6P7A7_RAT]	2E+08	2E+08	7E+07	4E+08	6E+08	5E+08
D3ZGU2	Protein Acss3 OS=Rattus norvegicus GN=Acss3 PE=4 SV=1 - [D3ZGU2_RAT]	1E+09	2E+09	1E+09	3E+09	3E+09	2E+09
Q8SEZ5	Cytochrome c oxidase subunit 2 OS=Rattus norvegicus GN=Mt-co2 PE=3 SV=1 - [Q8SEZ5_RAT]	1E+09	8E+08	4E+08	2E+09	2E+09	2E+09
G3V743	Glucosidase 1 OS=Rattus norvegicus GN=Mogs PE=4 SV=1 - [G3V743_RAT]	4E+08	2E+08	1E+08	6E+08	6E+08	6E+08
Q5M9I5	Cytochrome b-c1 complex subunit 6, mitochondrial OS=Rattus norvegicus GN=Uqcrh PE=3 SV=1 - [QCR6_RAT]	2E+08	1E+08	8E+07	7E+08	3E+08	7E+08
Q6TXE9	LRRGT00050 OS=Rattus norvegicus GN=Eprs PE=1 SV=1 - [Q6TXE9_RAT]	4E+08	6E+08	4E+08	8E+08	9E+08	8E+08
P41123	60S ribosomal protein L13 OS=Rattus norvegicus GN=Rpl13 PE=1 SV=2 - [RL13_RAT]	9E+08	8E+08	6E+08	2E+09	1E+09	2E+09
B0BNM1	NAD(P)H-hydrate epimerase OS=Rattus norvegicus GN=Apoa1bp PE=2 SV=1 - [NNRE_RAT]	7E+08	1E+09	5E+08	2E+09	1E+09	1E+09
P21913	Succinate dehydrogenase [ubiquinone] iron-sulfur subunit, mitochondrial OS=Rattus norvegicus GN=Sdhb PE=2 SV=2 - [SDHB_RAT]	1E+09	2E+09	7E+08	3E+09	2E+09	3E+09
G3V6I4	Protein Marc1 OS=Rattus norvegicus GN=Marc1 PE=4 SV=1 - [G3V6I4_RAT]	9E+08	5E+08	4E+08	1E+09	1E+09	1E+09
Q9WU49	Calcium-regulated heat stable protein 1 OS=Rattus norvegicus GN=Carhsp1 PE=1 SV=1 - [CHSP1_RAT]	5E+08	5E+08	7E+07	1E+09	9E+08	9E+08
Q64119	Myosin light polypeptide 6 OS=Rattus norvegicus GN=Myl6 PE=1 SV=3 - [MYL6_RAT]	7E+07	1E+08	1E+08	6E+08	7E+08	3E+08
Q6AXY8	Dehydrogenase/reductase (SDR family) member 1 OS=Rattus norvegicus GN=Dhrs1 PE=2 SV=1 - [Q6AXY8_RAT]	2E+08	1E+08	1E+08	2E+08	2E+08	3E+08
Q5M7T9	Threonine synthase-like 2 OS=Rattus norvegicus GN=Thnsl2 PE=2 SV=1 - [THNS2_RAT]	2E+08	3E+08	2E+08	4E+08	6E+08	5E+08
Q5M7U6	Actin-related protein 2 OS=Rattus norvegicus GN=Actr2 PE=1 SV=1 - [ARP2_RAT]	3E+08	3E+08	2E+08	5E+08	6E+08	6E+08
Q642A4	UPF0598 protein C8orf82 homolog OS=Rattus norvegicus PE=2 SV=1 - [CH082_RAT]	7E+07	1E+08	1E+08	2E+08	2E+08	3E+08
D3ZFY0	Protein Sephs1 OS=Rattus norvegicus GN=Sephs1 PE=4 SV=1 - [D3ZFY0_RAT]	3E+08	2E+08	1E+08	6E+08	5E+08	5E+08
R9PXU4	Thioredoxin reductase 1, cytoplasmic OS=Rattus norvegicus GN=Txnrd1 PE=3 SV=1 - [R9PXU4_RAT]	6E+08	5E+08	4E+08	9E+08	1E+09	1E+09
B1WC61	Acad9 protein OS=Rattus norvegicus GN=Acad9 PE=2 SV=1 - [B1WC61_RAT]	5E+08	7E+08	6E+08	8E+08	1E+09	1E+09
Q5XIG8	Serine-threonine kinase receptor-associated protein OS=Rattus norvegicus GN=Strap PE=1 SV=1 - [STRAP_RAT]	1E+08	1E+08	5E+07	3E+08	3E+08	2E+08
G3V709	Nicotinate phosphoribosyltransferase OS=Rattus norvegicus GN=Naprt1 PE=4 SV=1 - [G3V709_RAT]	1E+08	3E+08	2E+08	4E+08	6E+08	6E+08
Q63768	Adapter molecule crk OS=Rattus norvegicus GN=Crk PE=1 SV=1 - [CRK_RAT]	2E+08	7E+07	4E+07	2E+08	2E+08	3E+08
O35509	Ras-related protein Rab-11B OS=Rattus norvegicus GN=Rab11b PE=1 SV=4 - [RB11B_RAT]	2E+08	1E+08	1E+08	6E+08	3E+08	6E+08
D4AD70	Protein RGD1561636 OS=Rattus norvegicus GN=RGD1561636 PE=3 SV=1 - [D4AD70_RAT]	4E+08	3E+08	2E+08	8E+08	9E+08	1E+09
D4A5Q9	Glycine decarboxylase (Predicted), isoform CRA_a OS=Rattus norvegicus GN=Gldc PE=4 SV=1 - [D4A5Q9_RAT]	7E+08	1E+09	9E+08	2E+09	2E+09	2E+09
Q4AEF8	Coatomer subunit gamma-1 OS=Rattus norvegicus GN=Copg1 PE=2 SV=1 - [COPG1_RAT]	4E+08	5E+08	3E+08	5E+08	7E+08	8E+08
Q6AYT3	tRNA-splicing ligase RtcB homolog OS=Rattus norvegicus GN=Rtcb PE=2 SV=1 - [RTCB_RAT]	3E+08	2E+08	1E+08	4E+08	5E+08	5E+08
M0R6B4	Protein LOC100910002 OS=Rattus norvegicus GN=LOC100910002 PE=4 SV=1 - [M0R6B4_RAT]	8E+08	4E+08	2E+08	1E+09	1E+09	1E+09
Q5I0P2	Glycine cleavage system H protein, mitochondrial OS=Rattus norvegicus GN=Gcsh PE=2 SV=1 - [GCSH_RAT]	6E+08	7E+08	4E+08	2E+09	1E+09	2E+09
M0R735	Uncharacterized protein (Fragment) OS=Rattus norvegicus PE=4 SV=1 - [M0R735_RAT]	5E+07	8E+07	7E+07	2E+08	2E+08	1E+08
O55215	Ribosomal protein S2 OS=Rattus norvegicus GN=Rps2-ps6 PE=2 SV=1 - [O55215_RAT]	1E+09	6E+08	4E+08	2E+09	1E+09	1E+09
P97524	Very long-chain acyl-CoA synthetase OS=Rattus norvegicus GN=Slc27a2 PE=1 SV=1 - [S27A2_RAT]	9E+08	6E+08	4E+08	9E+08	1E+09	1E+09
B0BNA7	Eukaryotic translation initiation factor 3 subunit I OS=Rattus norvegicus GN=Eif3i PE=2 SV=1 - [EIF3I_RAT]	2E+08	2E+08	7E+07	3E+08	3E+08	3E+08
P10888	Cytochrome c oxidase subunit 4 isoform 1, mitochondrial OS=Rattus norvegicus GN=Cox4i1 PE=1 SV=1 - [COX41_RAT]	1E+09	5E+08	7E+08	2E+09	3E+09	2E+09
D3ZG43	NADH dehydrogenase (Ubiquinone) Fe-S protein 3 (Predicted), isoform CRA_c OS=Rattus norvegicus GN=Ndufs3 PE=3 SV=1 - [D3ZG43_RAT]	6E+08	3E+08	1E+08	7E+08	8E+08	1E+09
P07154	Cathepsin L1 OS=Rattus norvegicus GN=Ctsl PE=1 SV=2 - [CATL1_RAT]	8E+07	6E+07	4E+07	1E+08	2E+08	1E+08
Q7TPA1	Ab1-114 OS=Rattus norvegicus GN=Slc25a15 PE=2 SV=1 - [Q7TPA1_RAT]	3E+08	2E+08	1E+08	4E+08	4E+08	4E+08
F1LMZ8	26S proteasome non-ATPase regulatory subunit 11 OS=Rattus norvegicus GN=Psmd11 PE=3 SV=2 - [PSD11_RAT]	4E+08	3E+08	2E+08	5E+08	5E+08	4E+08
P60892	Ribose-phosphate pyrophosphokinase 1 OS=Rattus norvegicus GN=Prps1 PE=1 SV=2 - [PRPS1_RAT]	3E+08	2E+08	2E+08	5E+08	3E+08	4E+08
B5DF51	Membrane magnesium transporter 1 OS=Rattus norvegicus GN=Mmgt1 PE=2 SV=1 - [MMGT1_RAT]	6E+07	4E+07	0	1E+08	3E+08	2E+08

P51583	Multifunctional protein ADE2 OS=Rattus norvegicus GN=Paics PE=2 SV=3 - [PUR6_RAT]	2E+08	2E+08	2E+08	3E+08	4E+08	4E+08
G3V8B6	26S proteasome non-ATPase regulatory subunit 1 OS=Rattus norvegicus GN=Psmd1 PE=4 SV=1 - [G3V8B6_RAT]	9E+07	2E+08	3E+08	4E+08	6E+08	6E+08
Q6P3V9	60S ribosomal protein L4 OS=Rattus norvegicus GN=Rpl4 PE=2 SV=1 - [Q6P3V9_RAT]	2E+09	1E+09	7E+08	2E+09	2E+09	2E+09
B0BNE6	NADH dehydrogenase (Ubiquinone) Fe-S protein 8 (Predicted), isoform CRA_a OS=Rattus norvegicus GN=Ndufs8 PE=2 SV=1 - [B0BNE6_RAT]	2E+08	2E+08	2E+08	6E+08	4E+08	7E+08
B2RZ72	Actin related protein 2/3 complex, subunit 4 (Predicted), isoform CRA_a OS=Rattus norvegicus GN=Arpc4 PE=2 SV=1 - [B2RZ72_RAT]	3E+08	2E+08	2E+08	4E+08	4E+08	5E+08
F1LPM4	Amine oxidase [flavin-containing] B OS=Rattus norvegicus GN=Maob PE=4 SV=1 - [F1LPM4_RAT]	6E+08	4E+08	5E+08	1E+09	9E+08	1E+09
Q4KM65	Cleavage and polyadenylation specificity factor subunit 5 OS=Rattus norvegicus GN=Nudt21 PE=2 SV=1 - [CPSF5_RAT]	1E+08	4E+08	3E+08	1E+09	9E+08	5E+08
Q66HA6	ADP-ribosylation factor-like protein 8B OS=Rattus norvegicus GN=Arl8b PE=2 SV=1 - [ARL8B_RAT]	6E+07	3E+07	5E+07	1E+08	2E+08	1E+08
P20171	GTPase HRas OS=Rattus norvegicus GN=Hras PE=1 SV=2 - [RASH_RAT]	1E+07	5E+07	1E+07	1E+08	1E+08	2E+08
F1M572	Kynurenine--oxoglutarate transaminase 3 OS=Rattus norvegicus GN=Kat3 PE=4 SV=2 - [F1M572_RAT]	4E+09	3E+09	2E+09	8E+09	6E+09	7E+09
Q794E4	Heterogeneous nuclear ribonucleoprotein F OS=Rattus norvegicus GN=Hnrnpf PE=1 SV=3 - [HNRPF_RAT]	4E+08	2E+08	2E+08	6E+08	7E+08	7E+08
Q641X3	Beta-hexosaminidase subunit alpha OS=Rattus norvegicus GN=Hexa PE=2 SV=1 - [HEXA_RAT]	4E+08	4E+08	4E+08	5E+08	8E+08	8E+08
D4A904	N-acetylglutamate synthase (Predicted), isoform CRA_a OS=Rattus norvegicus GN=Nags PE=4 SV=1 - [D4A904_RAT]	3E+08	2E+08	2E+08	5E+08	3E+08	5E+08
D3ZJJ9	Uncharacterized protein OS=Rattus norvegicus GN=LOC100911814 PE=4 SV=2 - [D3ZJJ9_RAT]	7E+08	1E+09	9E+08	2E+09	2E+09	2E+09
B2GUZ5	F-actin-capping protein subunit alpha-1 OS=Rattus norvegicus GN=Capza1 PE=1 SV=1 - [CAZA1_RAT]	3E+08	2E+08	2E+08	6E+08	5E+08	5E+08
Q08163	Adenylyl cyclase-associated protein 1 OS=Rattus norvegicus GN=Cap1 PE=1 SV=3 - [CAP1_RAT]	3E+08	4E+08	3E+08	5E+08	5E+08	6E+08
B5DEJ6	Protein Tpk1 OS=Rattus norvegicus GN=Tpk1 PE=2 SV=1 - [B5DEJ6_RAT]	9E+07	1E+08	7E+07	2E+08	2E+08	2E+08
F1LT36	Protein RGD1564698 OS=Rattus norvegicus GN=RGD1564698 PE=4 SV=2 - [F1LT36_RAT]	9E+08	6E+08	7E+08	2E+09	2E+09	2E+09
Q6DGG0	Peptidyl-prolyl cis-trans isomerase D OS=Rattus norvegicus GN=Ppid PE=1 SV=3 - [PPID_RAT]	3E+08	3E+08	1E+08	7E+08	7E+08	5E+08
Q66HA8	Heat shock protein 105 kDa OS=Rattus norvegicus GN=Hsph1 PE=2 SV=1 - [HS105_RAT]	3E+08	5E+08	6E+08	9E+08	1E+09	1E+09
D3ZVI9	Parkinson disease 7 domain containing 1 (Predicted), isoform CRA_a OS=Rattus norvegicus GN=LOC100911365 PE=4 SV=1 - [D3ZVI9_RAT]	8E+06	2E+07	5E+07	8E+07	5E+07	7E+07
M0R6Y8	Phosphoglycerate kinase OS=Rattus norvegicus GN=RGD1560402 PE=3 SV=1 - [M0R6Y8_RAT]	9E+09	9E+09	4E+09	2E+10	2E+10	1E+10
B3DMA2	Acyl-CoA dehydrogenase family member 11 OS=Rattus norvegicus GN=Acad11 PE=1 SV=1 - [ACD11_RAT]	7E+08	1E+09	9E+08	2E+09	3E+09	2E+09
D3ZF07	Protein RGD1562402 OS=Rattus norvegicus GN=RGD1562402 PE=3 SV=1 - [D3ZF07_RAT]	4E+08	3E+08	5E+08	1E+09	1E+09	1E+09
F2Z3Q8	Importin subunit beta-1 OS=Rattus norvegicus GN=Kpnb1 PE=4 SV=1 - [F2Z3Q8_RAT]	3E+08	4E+08	3E+08	9E+08	9E+08	7E+08
P01015	Angiotensinogen OS=Rattus norvegicus GN=Agt PE=1 SV=1 - [ANGT_RAT]	2E+08	2E+08	1E+08	3E+08	3E+08	3E+08
P80067	Dipeptidyl peptidase 1 OS=Rattus norvegicus GN=Ctsc PE=1 SV=3 - [CATC_RAT]	1E+09	1E+09	1E+09	2E+09	3E+09	2E+09
B2RYJ7	ARP1 actin-related protein 1 homolog B (Yeast) OS=Rattus norvegicus GN=Actr1b PE=2 SV=1 - [B2RYJ7_RAT]	1E+08	1E+08	1E+08	2E+08	2E+08	2E+08
D3ZYL4	Mitochondrial ribosomal protein L50 (Predicted) OS=Rattus norvegicus GN=Mrpl50 PE=4 SV=1 - [D3ZYL4_RAT]	0	0	3E+07	8E+07	4E+07	7E+07
Q5XIN6	LETM1 and EF-hand domain-containing protein 1, mitochondrial OS=Rattus norvegicus GN=Letm1 PE=1 SV=1 - [LETM1_RAT]	5E+08	5E+08	4E+08	6E+08	7E+08	7E+08
D3Z941	Protein Mars OS=Rattus norvegicus GN=Mars PE=3 SV=1 - [D3Z941_RAT]	3E+08	3E+08	3E+08	4E+08	5E+08	5E+08
Q499P2	Leukotriene A-4 hydrolase OS=Rattus norvegicus GN=Lta4h PE=2 SV=1 - [Q499P2_RAT]	3E+08	3E+08	4E+08	6E+08	8E+08	6E+08
B2RYS8	NADH dehydrogenase (Ubiquinone) 1 beta subcomplex 8 OS=Rattus norvegicus GN=Ndufb8 PE=2 SV=1 - [B2RYS8_RAT]	1E+08	8E+07	8E+07	3E+08	2E+08	3E+08
B5DES0	Protein Snrpd2 OS=Rattus norvegicus GN=Snrpd2 PE=4 SV=1 - [B5DES0_RAT]	1E+08	6E+07	9E+07	3E+08	4E+08	2E+08
F1LSP2	Protein Acad10 OS=Rattus norvegicus GN=Acad10 PE=3 SV=2 - [F1LSP2_RAT]	5E+08	3E+08	5E+08	8E+08	1E+09	6E+08
G3V7W1	Programmed cell death 6 (Predicted), isoform CRA_a OS=Rattus norvegicus GN=Pdcd6 PE=4 SV=1 - [G3V7W1_RAT]	8E+07	8E+07	1E+08	3E+08	3E+08	2E+08
Q5FVN1	Starch-binding domain-containing protein 1 OS=Rattus norvegicus GN=Stbd1 PE=2 SV=1 - [STBD1_RAT]	2E+08	1E+08	1E+08	6E+08	3E+08	4E+08
F1LM33	Leucine-rich PPR motif-containing protein, mitochondrial OS=Rattus norvegicus GN=Lrpprc PE=4 SV=2 - [F1LM33_RAT]	3E+08	3E+08	3E+08	4E+08	7E+08	6E+08
P62907	60S ribosomal protein L10a OS=Rattus norvegicus GN=Rpl10a PE=1 SV=2 - [RL10A_RAT]	1E+08	1E+08	9E+07	4E+08	6E+08	7E+08
Q6PDV7	60S ribosomal protein L10 OS=Rattus norvegicus GN=Rpl10 PE=1 SV=3 - [RL10_RAT]	1E+08	4E+08	1E+08	9E+08	6E+08	7E+08
M0R907	Protein Snrpd3 OS=Rattus norvegicus GN=Snrpd3 PE=4 SV=1 - [M0R907_RAT]	1E+08	2E+07	6E+07	3E+08	4E+08	4E+08
M0R634	Uncharacterized protein (Fragment) OS=Rattus norvegicus PE=3 SV=1 - [M0R634_RAT]	1E+10	9E+09	6E+09	2E+10	2E+10	2E+10
Q5U2S7	Proteasome (Prosome, macropain) 26S subunit, non-ATPase, 3 OS=Rattus norvegicus GN=Psmd3 PE=2 SV=1 - [Q5U2S7_RAT]	1E+08	2E+08	2E+08	5E+08	4E+08	5E+08
P00787	Cathepsin B OS=Rattus norvegicus GN=Ctsb PE=1 SV=2 - [CATB_RAT]	8E+08	1E+09	2E+08	2E+09	1E+09	2E+09
Q641Y8	ATP-dependent RNA helicase DDX1 OS=Rattus norvegicus GN=Ddx1 PE=2 SV=1 - [DDX1_RAT]	1E+08	2E+08	1E+08	3E+08	3E+08	3E+08
G3V816	Nucleoside diphosphate kinase OS=Rattus norvegicus GN=Nme3 PE=3 SV=1 - [G3V816_RAT]	4E+07	3E+07	0	1E+08	8E+07	9E+07
G3V7V4	Ectonucleotide pyrophosphatase/phosphodiesterase family member 1 OS=Rattus norvegicus GN=Enpp1 PE=1 SV=2 - [G3V7V4_RAT]	2E+08	9E+08	5E+08	1E+09	1E+09	1E+09
Q07984	Translocon-associated protein subunit delta OS=Rattus norvegicus GN=Ssr4 PE=2 SV=1 - [SSRD_RAT]	2E+08	2E+08	1E+08	5E+08	5E+08	5E+08
F1M4T3	Protein Erh (Fragment) OS=Rattus norvegicus GN=Erh PE=4 SV=1 - [F1M4T3_RAT]	3E+07	0	0	1E+08	1E+08	1E+08
P15178	Aspartate--tRNA ligase, cytoplasmic OS=Rattus norvegicus GN=Dars PE=2 SV=1 - [SYDC_RAT]	3E+08	4E+08	3E+08	5E+08	6E+08	6E+08
P35565	Calnexin OS=Rattus norvegicus GN=Canx PE=1 SV=1 - [CALX_RAT]	3E+09	3E+09	2E+09	4E+09	4E+09	5E+09
D4AAE9	Protein Cisd2 OS=Rattus norvegicus GN=Cisd2 PE=4 SV=1 - [D4AAE9_RAT]	2E+08	1E+08	2E+08	4E+08	4E+08	4E+08
P41542	General vesicular transport factor p115 OS=Rattus norvegicus GN=Uso1 PE=1 SV=1 - [USO1_RAT]	3E+08	4E+08	2E+08	7E+08	9E+08	9E+08
D4A6G6	Protein LOC100362339 OS=Rattus norvegicus GN=LOC100362339 PE=4 SV=1 - [D4A6G6_RAT]	7E+08	6E+08	8E+08	2E+09	2E+09	2E+09
F1LPS8	Transcriptional activator protein Pur-alpha OS=Rattus norvegicus GN=Pura PE=4 SV=2 - [F1LPS8_RAT]	6E+08	5E+08	3E+08	1E+09	1E+09	8E+08
P19139	Casein kinase II subunit alpha OS=Rattus norvegicus GN=Csnk2a1 PE=1 SV=2 - [CSK21_RAT]	1E+08	1E+08	4E+07	3E+08	2E+08	2E+08

P62755	40S ribosomal protein S6 OS=Rattus norvegicus GN=Rps6 PE=1 SV=1 - [RS6_RAT]	5E+08	6E+08	4E+08	1E+09	1E+09	1E+09
O88202	60 kDa lysophospholipase OS=Rattus norvegicus GN=Aspg PE=1 SV=1 - [LPP60_RAT]	7E+08	4E+08	3E+08	1E+09	9E+08	1E+09
P53809	Phosphatidylcholine transfer protein OS=Rattus norvegicus GN=Pctp PE=2 SV=2 - [PPCT_RAT]	0	3E+07	0	1E+08	2E+08	8E+07
M0R959	Protein Mmab OS=Rattus norvegicus GN=Mmab PE=4 SV=1 - [M0R959_RAT]	0	3E+07	6E+07	1E+08	9E+07	1E+08
D3ZA93	Protein Acot13 OS=Rattus norvegicus GN=Acot13 PE=4 SV=1 - [D3ZA93_RAT]	1E+08	2E+08	2E+08	5E+08	5E+08	4E+08
B2RYS2	Cytochrome b-c1 complex subunit 7 OS=Rattus norvegicus GN=LOC685596 PE=3 SV=1 - [B2RYS2_RAT]	3E+08	2E+08	3E+08	1E+09	1E+09	9E+08
Q5U2U5	Perilipin OS=Rattus norvegicus GN=Plin2 PE=2 SV=1 - [Q5U2U5_RAT]	3E+08	3E+08	0	4E+08	5E+08	6E+08
B2RZ66	Protein Srp19 OS=Rattus norvegicus GN=Srp19 PE=2 SV=1 - [B2RZ66_RAT]	0	5E+07	0	6E+07	1E+08	1E+08
D4A4Q4	Protein Ociad2 OS=Rattus norvegicus GN=Ociad2 PE=4 SV=2 - [D4A4Q4_RAT]	3E+07	3E+07	0	5E+07	7E+07	1E+08
B0K031	60S ribosomal protein L7 OS=Rattus norvegicus GN=Rpl7 PE=2 SV=1 - [B0K031_RAT]	2E+09	1E+09	7E+08	2E+09	2E+09	2E+09
Q9JJ54-4	Isoform 4 of Heterogeneous nuclear ribonucleoprotein D0 OS=Rattus norvegicus GN=Hnrnpd - [HNRPD_RAT]	6E+08	2E+08	3E+08	1E+09	2E+09	1E+09
M0R7P0	Protein LOC100912027 OS=Rattus norvegicus GN=LOC100912027 PE=4 SV=1 - [M0R7P0_RAT]	3E+08	3E+08	5E+08	1E+09	1E+09	8E+08
M0RCF2	Uncharacterized protein OS=Rattus norvegicus PE=3 SV=1 - [M0RCF2_RAT]	9E+09	5E+09	4E+09	2E+10	1E+10	1E+10
P62828	GTP-binding nuclear protein Ran OS=Rattus norvegicus GN=Ran PE=1 SV=3 - [RAN_RAT]	5E+08	3E+08	2E+08	1E+09	8E+08	9E+08
P34067	Proteasome subunit beta type-4 OS=Rattus norvegicus GN=Psmb4 PE=1 SV=2 - [PSB4_RAT]	8E+07	3E+08	8E+07	5E+08	3E+08	4E+08
P62850-2	Isoform 2 of 40S ribosomal protein S24 OS=Rattus norvegicus GN=Rps24 - [RS24_RAT]	2E+08	4E+08	9E+07	7E+08	6E+08	7E+08
Q6AYD3	Proliferation-associated 2G4 OS=Rattus norvegicus GN=Pa2g4 PE=2 SV=1 - [Q6AYD3_RAT]	2E+08	3E+08	1E+08	4E+08	5E+08	4E+08
G3V6H5	Mitochondrial 2-oxoglutarate/malate carrier protein OS=Rattus norvegicus GN=Slc25a11 PE=3 SV=1 - [G3V6H5_RAT]	1E+08	9E+07	4E+07	2E+08	2E+08	2E+08
Q62902	Protein ERGIC-53 OS=Rattus norvegicus GN=Lman1 PE=1 SV=1 - [LMAN1_RAT]	6E+08	6E+08	2E+08	1E+09	1E+09	1E+09
Q9Z1P2	Alpha-actinin-1 OS=Rattus norvegicus GN=Actn1 PE=1 SV=1 - [ACTN1_RAT]	1E+09	1E+09	1E+09	2E+09	2E+09	1E+09
D3ZQB6	Cat eye syndrome chromosome region, candidate 5 homolog (Human) (Predicted) OS=Rattus norvegicus GN=Cecr5 PE=4 SV=1 - [D3ZQB6_RAT]	1E+08	8E+07	5E+07	3E+08	3E+08	2E+08
D3ZPP2	Protein Arl8a OS=Rattus norvegicus GN=Arl8a PE=3 SV=1 - [D3ZPP2_RAT]	6E+07	3E+07	5E+07	1E+08	2E+08	1E+08
D3ZJF4	Uncharacterized protein (Fragment) OS=Rattus norvegicus GN=LOC100360986 PE=4 SV=2 - [D3ZJF4_RAT]	7E+08	4E+08	8E+07	2E+09	2E+09	2E+09
P62718	60S ribosomal protein L18a OS=Rattus norvegicus GN=Rpl18a PE=2 SV=1 - [RL18A_RAT]	5E+08	4E+08	5E+08	1E+09	1E+09	1E+09
P49242	40S ribosomal protein S3a OS=Rattus norvegicus GN=Rps3a PE=1 SV=2 - [RS3A_RAT]	1E+09	7E+08	5E+08	2E+09	1E+09	2E+09
Q5RKJ9	RAB10, member RAS oncogene family OS=Rattus norvegicus GN=Rab10 PE=2 SV=1 - [Q5RKJ9_RAT]	7E+08	6E+08	5E+08	9E+08	9E+08	1E+09
P29314	40S ribosomal protein S9 OS=Rattus norvegicus GN=Rps9 PE=1 SV=4 - [RS9_RAT]	5E+08	2E+08	2E+08	1E+09	9E+08	8E+08
M0R5N4	Protein Pfdn4 OS=Rattus norvegicus GN=Pfdn4 PE=4 SV=1 - [M0R5N4_RAT]	0	2E+07	7E+07	2E+08	9E+07	2E+08
Q7TP78	Aa2-258 OS=Rattus norvegicus GN=Ndufa8 PE=2 SV=1 - [Q7TP78_RAT]	2E+08	1E+08	3E+08	5E+08	5E+08	4E+08
Q6MG66	LSM2 homolog, U6 small nuclear RNA associated [S. cerevisiae] OS=Rattus norvegicus GN=Lsm2 PE=4 SV=1 - [Q6MG66_RAT]	3E+07	0	0	2E+08	8E+07	2E+08
Q68FT7	Phenylalanyl-tRNA synthetase, beta subunit OS=Rattus norvegicus GN=Farsb PE=2 SV=1 - [Q68FT7_RAT]	1E+08	3E+08	2E+08	4E+08	4E+08	3E+08
Q64581	Cytochrome P450 3A18 OS=Rattus norvegicus GN=Cyp3a18 PE=2 SV=1 - [CP3AI_RAT]	6E+08	4E+08	2E+08	1E+09	1E+09	8E+08
Q66H80	Coatomer subunit delta OS=Rattus norvegicus GN=Arcn1 PE=2 SV=1 - [COPD_RAT]	3E+08	2E+08	8E+07	3E+08	4E+08	3E+08
F1LNF7	Isocitrate dehydrogenase [NAD] subunit alpha, mitochondrial OS=Rattus norvegicus GN=Idh3a PE=3 SV=1 - [F1LNF7_RAT]	5E+08	4E+08	2E+08	1E+09	1E+09	8E+08
Q498D5	Regulator of microtubule dynamics protein 2 OS=Rattus norvegicus GN=Rmdn2 PE=2 SV=1 - [RMD2_RAT]	9E+07	4E+07	3E+07	1E+08	2E+08	2E+08
P24051	40S ribosomal protein S27-like OS=Rattus norvegicus GN=Rps27l PE=1 SV=3 - [RS27L_RAT]	6E+07	2E+08	8E+07	2E+08	4E+08	4E+08
F1LNG8	Protein Mpv17 OS=Rattus norvegicus GN=Mpv17 PE=4 SV=1 - [F1LNG8_RAT]	0	0	6E+06	2E+07	3E+07	3E+07
G3V936	Citrate synthase OS=Rattus norvegicus GN=Cs PE=3 SV=1 - [G3V936_RAT]	2E+09	2E+09	8E+08	3E+09	3E+09	2E+09
D3ZVS2	L-2-hydroxyglutarate dehydrogenase (Predicted) OS=Rattus norvegicus GN=L2hgdh PE=4 SV=1 - [D3ZVS2_RAT]	3E+08	2E+08	1E+08	5E+08	5E+08	4E+08
P62944	AP-2 complex subunit beta OS=Rattus norvegicus GN=Ap2b1 PE=1 SV=1 - [AP2B1_RAT]	3E+08	3E+08	4E+08	8E+08	8E+08	7E+08
Q5RKI1	Eukaryotic initiation factor 4A-II OS=Rattus norvegicus GN=Eif4a2 PE=1 SV=1 - [IF4A2_RAT]	1E+09	1E+09	9E+08	2E+09	2E+09	2E+09
G3V6P7	Myosin, heavy polypeptide 9, non-muscle OS=Rattus norvegicus GN=LOC100911597 PE=4 SV=1 - [G3V6P7_RAT]	2E+08	2E+08	2E+08	1E+09	2E+09	3E+09
Q9QX80	CArG-binding factor A OS=Rattus norvegicus GN=Hnrnpab PE=2 SV=1 - [Q9QX80_RAT]	3E+08	1E+08	2E+08	7E+08	6E+08	5E+08
F1LUV3	Uncharacterized protein (Fragment) OS=Rattus norvegicus PE=3 SV=2 - [F1LUV3_RAT]	1E+09	9E+08	8E+08	3E+09	2E+09	2E+09
Q07439	Heat shock 70 kDa protein 1A/1B OS=Rattus norvegicus GN=Hspa1a PE=2 SV=2 - [HSP71_RAT]	3E+09	4E+09	4E+09	7E+09	8E+09	6E+09
Q04462	Valine--tRNA ligase OS=Rattus norvegicus GN=Vars PE=2 SV=2 - [SYVC_RAT]	4E+08	4E+08	3E+08	6E+08	7E+08	6E+08
F1LQJ7	Protein Pck2 OS=Rattus norvegicus GN=Pck2 PE=3 SV=2 - [F1LQJ7_RAT]	6E+08	9E+08	5E+08	1E+09	1E+09	1E+09
R9PXU9	Osteoclast-stimulating factor 1 (Fragment) OS=Rattus norvegicus GN=Ostf1 PE=4 SV=1 - [R9PXU9_RAT]	2E+08	1E+08	6E+07	5E+08	3E+08	6E+08
D4AEP0	Adenylosuccinate synthetase isozyme 2 OS=Rattus norvegicus GN=Adss PE=3 SV=1 - [D4AEP0_RAT]	3E+08	2E+08	1E+08	4E+08	3E+08	4E+08
R9PXZ2	Heterogeneous nuclear ribonucleoprotein Q (Fragment) OS=Rattus norvegicus GN=Syncrip PE=4 SV=1 - [R9PXZ2_RAT]	3E+08	4E+08	3E+08	7E+08	8E+08	5E+08
D4A7F9	Glyceraldehyde-3-phosphate dehydrogenase OS=Rattus norvegicus PE=3 SV=1 - [D4A7F9_RAT]	4E+09	2E+09	1E+09	6E+09	5E+09	5E+09
P06866	Haptoglobin OS=Rattus norvegicus GN=Hp PE=1 SV=3 - [HPT_RAT]	1E+09	6E+08	6E+08	2E+09	2E+09	2E+09
R9PXR7	Prostaglandin E synthase 3 (Fragment) OS=Rattus norvegicus GN=Ptges3 PE=1 SV=1 - [R9PXR7_RAT]	3E+08	5E+08	6E+08	1E+09	1E+09	1E+09
D3Z9X1	Uncharacterized protein (Fragment) OS=Rattus norvegicus PE=3 SV=2 - [D3Z9X1_RAT]	6E+09	7E+09	5E+09	1E+10	1E+10	1E+10
Q4FZT9	26S proteasome non-ATPase regulatory subunit 2 OS=Rattus norvegicus GN=Psmd2 PE=2 SV=1 - [PSMD2_RAT]	3E+08	5E+08	3E+08	8E+08	8E+08	6E+08
A3KN98	Bco2 protein OS=Rattus norvegicus GN=Bco2 PE=2 SV=1 - [A3KN98_RAT]	2E+08	2E+08	2E+08	4E+08	4E+08	3E+08

Q5XI86	Peptidyl-tRNA hydrolase 2 OS=Rattus norvegicus GN=Ptrh2 PE=1 SV=1 - [Q5XI86_RAT]	5E+07	3E+07	0	1E+08	8E+07	1E+08
F1M395	Protein LOC100361732 (Fragment) OS=Rattus norvegicus GN=LOC100361732 PE=4 SV=2 - [F1M395_RAT]	7E+08	5E+08	8E+08	2E+09	2E+09	2E+09
Q4V8J5	DNA-damage inducible protein 2 OS=Rattus norvegicus GN=Ddi2 PE=2 SV=1 - [Q4V8J5_RAT]	3E+08	2E+08	3E+08	4E+08	4E+08	4E+08
P33274	Cytochrome P450 4F1 OS=Rattus norvegicus GN=Cyp4f1 PE=2 SV=1 - [CP4F1_RAT]	5E+08	3E+08	2E+08	9E+08	9E+08	1E+09
D3ZAF6	ATP synthase subunit f, mitochondrial OS=Rattus norvegicus GN=Atp5j2 PE=1 SV=1 - [ATPK_RAT]	1E+09	5E+08	1E+09	1E+09	2E+09	2E+09
P23514	Coatomer subunit beta OS=Rattus norvegicus GN=Copb1 PE=1 SV=1 - [COPB_RAT]	5E+08	6E+08	3E+08	7E+08	9E+08	8E+08
Q5XIG9	Mitochondrial protein 18 kDa OS=Rattus norvegicus GN=Mtfp1 PE=2 SV=1 - [Q5XIG9_RAT]	0	0	3E+07	1E+08	1E+08	1E+08
Q06884	Cytochrome P-450 OS=Rattus norvegicus GN=Cyp3a73 PE=2 SV=1 - [Q06884_RAT]	1E+09	7E+08	7E+08	2E+09	3E+09	2E+09
D4A2W9	Protein Samhd1 OS=Rattus norvegicus GN=Samhd1 PE=4 SV=2 - [D4A2W9_RAT]	7E+07	4E+07	1E+08	2E+08	3E+08	2E+08
O35814	Stress-induced-phosphoprotein 1 OS=Rattus norvegicus GN=Stip1 PE=1 SV=1 - [STIP1_RAT]	1E+09	7E+08	5E+08	1E+09	1E+09	2E+09
P51590	Cytochrome P450 2J3 OS=Rattus norvegicus GN=Cyp2j3 PE=2 SV=1 - [CP2J3_RAT]	1E+08	8E+07	5E+07	3E+08	3E+08	3E+08
Q63413	Spliceosome RNA helicase Ddx39b OS=Rattus norvegicus GN=Ddx39b PE=1 SV=3 - [DX39B_RAT]	2E+08	3E+08	2E+08	3E+08	4E+08	4E+08
G3V9N0	Pabpc4 protein OS=Rattus norvegicus GN=Pabpc4 PE=2 SV=2 - [G3V9N0_RAT]	1E+09	7E+08	4E+08	1E+09	1E+09	2E+09
O54975	Xaa-Pro aminopeptidase 1 OS=Rattus norvegicus GN=Xpnpep1 PE=1 SV=1 - [XPP1_RAT]	3E+08	2E+08	3E+08	4E+08	5E+08	5E+08
M0R9M4	Uncharacterized protein (Fragment) OS=Rattus norvegicus PE=3 SV=1 - [M0R9M4_RAT]	3E+09	4E+09	3E+09	9E+09	7E+09	7E+09
P83732	60S ribosomal protein L24 OS=Rattus norvegicus GN=Rpl24 PE=2 SV=1 - [RL24_RAT]	5E+08	5E+08	5E+08	1E+09	2E+09	1E+09
G3V8Q6	Protein kinase, cAMP-dependent, regulatory, type 2, alpha, isoform CRA_a OS=Rattus norvegicus GN=Prkar2a PE=1 SV=1 - [G3V8Q6_RAT]	2E+08	2E+08	2E+08	3E+08	3E+08	4E+08
M0RCU5	Protein Urad OS=Rattus norvegicus GN=Urad PE=4 SV=1 - [M0RCU5_RAT]	1E+09	9E+08	6E+08	2E+09	1E+09	2E+09
D3ZP47	Phosphohistidine phosphatase 1 (Predicted), isoform CRA_a OS=Rattus norvegicus GN=Phpt1 PE=4 SV=1 - [D3ZP47_RAT]	3E+07	5E+07	8E+07	3E+08	2E+08	3E+08
P47875	Cysteine and glycine-rich protein 1 OS=Rattus norvegicus GN=Csrp1 PE=2 SV=2 - [CSRP1_RAT]	1E+08	6E+07	1E+08	2E+08	3E+08	2E+08
F1LPC7	Hepatoma-derived growth factor OS=Rattus norvegicus GN=Hdgf PE=1 SV=1 - [F1LPC7_RAT]	1E+08	9E+07	9E+07	2E+08	2E+08	2E+08
Q64638	UDP-glucuronosyltransferase 1-5 OS=Rattus norvegicus GN=Ugt1a5 PE=2 SV=1 - [UD15_RAT]	6E+09	4E+09	2E+09	7E+09	6E+09	7E+09
Q3KR94	Protein Vtn OS=Rattus norvegicus GN=Vtn PE=2 SV=1 - [Q3KR94_RAT]	2E+08	4E+08	4E+08	7E+08	6E+08	6E+08
D4ADM5	Uncharacterized protein OS=Rattus norvegicus GN=LOC680217 PE=4 SV=1 - [D4ADM5_RAT]	1E+09	1E+09	7E+08	3E+09	4E+09	2E+09
D4ADT5	Protein Ddx58 OS=Rattus norvegicus GN=Ddx58 PE=4 SV=2 - [D4ADT5_RAT]	2E+08	2E+08	1E+08	3E+08	4E+08	5E+08
Q5XIM7	Lysine--tRNA ligase OS=Rattus norvegicus GN=Kars PE=2 SV=1 - [Q5XIM7_RAT]	2E+08	3E+08	3E+08	6E+08	7E+08	6E+08
D3ZEB8	40S ribosomal protein S12 OS=Rattus norvegicus PE=3 SV=1 - [D3ZEB8_RAT]	3E+08	2E+08	4E+08	9E+08	1E+09	1E+09
F1M7X5	Dipeptidyl peptidase 4 OS=Rattus norvegicus GN=Dpp4 PE=4 SV=1 - [F1M7X5_RAT]	1E+09	1E+09	1E+09	2E+09	2E+09	2E+09
G3V8L1	PYD and CARD domain containing OS=Rattus norvegicus GN=Pycard PE=4 SV=1 - [G3V8L1_RAT]	0	2E+07	0	1E+08	6E+07	9E+07
Q66H15	Regulator of microtubule dynamics protein 3 OS=Rattus norvegicus GN=Rmdn3 PE=2 SV=1 - [RMD3_RAT]	7E+07	7E+07	7E+07	1E+08	1E+08	9E+07
G3V8A5	Vacuolar protein sorting-associated protein 35 OS=Rattus norvegicus GN=Vps35 PE=3 SV=1 - [G3V8A5_RAT]	5E+08	5E+08	7E+08	2E+09	1E+09	9E+08
Q5U302	Catenin (Cadherin associated protein), alpha 1 OS=Rattus norvegicus GN=Ctnna1 PE=1 SV=1 - [Q5U302_RAT]	1E+08	2E+08	2E+08	2E+08	4E+08	4E+08
Q9Z0J5-2	Isoform 2 of Thioredoxin reductase 2, mitochondrial OS=Rattus norvegicus GN=Txnrd2 - [TRXR2_RAT]	6E+08	4E+08	4E+08	7E+08	7E+08	9E+08
E9PST1	Protein RGD1310507 OS=Rattus norvegicus GN=RGD1310507 PE=4 SV=2 - [E9PST1_RAT]	1E+07	1E+08	1E+08	2E+08	3E+08	2E+08
Q9EST6	Acidic leucine-rich nuclear phosphoprotein 32 family member B OS=Rattus norvegicus GN=Anp32b PE=2 SV=1 - [AN32B_RAT]	3E+08	3E+08	2E+08	7E+08	6E+08	7E+08
F1LYI7	Protein LOC100913000 OS=Rattus norvegicus GN=Tmem256 PE=4 SV=2 - [F1LYI7_RAT]	1E+08	7E+07	1E+08	2E+08	3E+08	2E+08
Q5BJQ0	Chaperone activity of bc1 complex-like, mitochondrial OS=Rattus norvegicus GN=Adck3 PE=2 SV=1 - [ADCK3_RAT]	8E+08	6E+08	4E+08	9E+08	1E+09	1E+09
F1LNM4	Protein LOC100909666 OS=Rattus norvegicus GN=LOC100909666 PE=4 SV=2 - [F1LNM4_RAT]	3E+08	3E+08	3E+08	5E+08	5E+08	4E+08
D4A9L2	Protein Srsf1 OS=Rattus norvegicus GN=Srsf1 PE=4 SV=1 - [D4A9L2_RAT]	1E+07	0	1E+07	2E+08	1E+08	1E+08
M0R9D9	Uncharacterized protein (Fragment) OS=Rattus norvegicus PE=4 SV=1 - [M0R9D9_RAT]	1E+08	8E+07	5E+07	3E+08	2E+08	2E+08
H7C5Y5	60S ribosomal protein L6 OS=Rattus norvegicus GN=Rpl6 PE=3 SV=1 - [H7C5Y5_RAT]	1E+09	8E+08	4E+08	1E+09	2E+09	1E+09
P41565	Isocitrate dehydrogenase [NAD] subunit gamma 1, mitochondrial OS=Rattus norvegicus GN=Idh3g PE=2 SV=2 - [IDHG1_RAT]	2E+08	9E+07	8E+07	4E+08	3E+08	2E+08
P62278	40S ribosomal protein S13 OS=Rattus norvegicus GN=Rps13 PE=1 SV=2 - [RS13_RAT]	8E+08	5E+08	8E+08	2E+09	2E+09	2E+09
Q5FVL2	ER membrane protein complex subunit 8 OS=Rattus norvegicus GN=Emc8 PE=2 SV=1 - [EMC8_RAT]	6E+07	4E+07	0	2E+08	2E+08	2E+08
P62815	V-type proton ATPase subunit B, brain isoform OS=Rattus norvegicus GN=Atp6v1b2 PE=1 SV=1 - [VATB2_RAT]	3E+08	2E+08	2E+08	4E+08	5E+08	5E+08
D3ZS58	NADH dehydrogenase [ubiquinone] 1 alpha subcomplex subunit 2 OS=Rattus norvegicus GN=Ndufa2 PE=3 SV=1 - [D3ZS58_RAT]	3E+08	3E+08	3E+08	7E+08	9E+08	6E+08
O08776-4	Isoform 4 of NADH dehydrogenase [ubiquinone] 1 alpha subcomplex assembly factor 3 OS=Rattus norvegicus GN=Ndufaf3 - [NDUF3_RAT]	5E+07	0	0	6E+07	6E+07	8E+07
Q6IMY8	Heterogeneous nuclear ribonucleoprotein U OS=Rattus norvegicus GN=Hnrnpu PE=2 SV=1 - [Q6IMY8_RAT]	6E+08	5E+08	5E+08	1E+09	1E+09	2E+09
Q3KR86	Mitochondrial inner membrane protein (Fragment) OS=Rattus norvegicus GN=Immt PE=1 SV=1 - [IMMT_RAT]	1E+08	2E+08	2E+08	6E+08	5E+08	4E+08
Q68A21	Transcriptional activator protein Pur-beta OS=Rattus norvegicus GN=Purb PE=1 SV=3 - [PURB_RAT]	1E+08	8E+07	7E+07	3E+08	2E+08	2E+08
M0RAS6	Uncharacterized protein OS=Rattus norvegicus PE=4 SV=1 - [M0RAS6_RAT]	2E+08	1E+08	8E+07	4E+08	4E+08	3E+08
G3V940	Coronin OS=Rattus norvegicus GN=Coro1b PE=3 SV=1 - [G3V940_RAT]	2E+08	2E+08	3E+08	5E+08	5E+08	4E+08
P08516	Cytochrome P450 4A10 OS=Rattus norvegicus GN=Cyp4a10 PE=1 SV=2 - [CP4AA_RAT]	2E+08	1E+08	7E+07	7E+08	5E+08	5E+08
B2RYQ2	Protein Ppp2r4 OS=Rattus norvegicus GN=Ppp2r4 PE=2 SV=1 - [B2RYQ2_RAT]	1E+08	8E+07	1E+08	3E+08	2E+08	2E+08
D4ADE8	DEAD/H (Asp-Glu-Ala-Asp/His) box polypeptide 3, X-linked OS=Rattus norvegicus GN=Ddx3x PE=4 SV=2 - [D4ADE8_RAT]	3E+08	5E+08	3E+08	6E+08	7E+08	7E+08
B5DFN4	Prefoldin 5 (Predicted), isoform CRA_a OS=Rattus norvegicus GN=Pfdn5 PE=2 SV=1 - [B5DFN4_RAT]	0	2E+06	0	1E+08	5E+07	1E+08

D4A774	Uncharacterized protein (Fragment) OS=Rattus norvegicus GN=LOC100912610 PE=3 SV=2 - [D4A774_RAT]	2E+09	1E+09	2E+09	5E+09	6E+09	5E+09
D3ZYW2	Heterogeneous nuclear ribonucleoprotein H OS=Rattus norvegicus GN=Hnrnph1 PE=4 SV=1 - [D3ZYW2_RAT]	5E+08	3E+08	2E+08	9E+08	8E+08	8E+08
F8WG91	ADP-ribosylation factor-like protein 3 (Fragment) OS=Rattus norvegicus GN=Arl3 PE=3 SV=1 - [F8WG91_RAT]	0	7E+07	5E+07	2E+08	1E+08	1E+08
G3V6K0	COMM domain containing 5, isoform CRA_a OS=Rattus norvegicus GN=Commd5 PE=4 SV=1 - [G3V6K0_RAT]	0	6E+07	2E+07	2E+08	1E+08	9E+07
B2RYN3	Eukaryotic translation elongation factor 1 epsilon 1 OS=Rattus norvegicus GN=Eef1e1 PE=2 SV=1 - [B2RYN3_RAT]	8E+07	5E+07	2E+08	3E+08	2E+08	3E+08
F1M5X1	Protein Rrbp1 OS=Rattus norvegicus GN=Rrbp1 PE=4 SV=2 - [F1M5X1_RAT]	1E+09	2E+09	1E+09	2E+09	3E+09	2E+09
D4A8P9	Protein Iars2 OS=Rattus norvegicus GN=Iars2 PE=3 SV=2 - [D4A8P9_RAT]	2E+08	5E+08	3E+08	9E+08	6E+08	6E+08
F7EUK4	Kininogen-1 OS=Rattus norvegicus GN=Kng1l1 PE=4 SV=1 - [F7EUK4_RAT]	2E+08	4E+07	1E+08	4E+08	5E+08	4E+08
P62859	40S ribosomal protein S28 OS=Rattus norvegicus GN=Rps28 PE=1 SV=1 - [RS28_RAT]	6E+08	5E+08	4E+08	2E+09	2E+09	1E+09
P60901	Proteasome subunit alpha type-6 OS=Rattus norvegicus GN=Psma6 PE=1 SV=1 - [PSA6_RAT]	2E+08	4E+08	2E+08	9E+08	6E+08	6E+08
Q5XI38	Lymphocyte cytosolic protein 1 OS=Rattus norvegicus GN=Lcp1 PE=2 SV=1 - [Q5XI38_RAT]	4E+08	4E+08	3E+08	8E+08	9E+08	8E+08
D4AB17	Protein LOC100912917 OS=Rattus norvegicus GN=LOC100912917 PE=4 SV=2 - [D4AB17_RAT]	3E+08	4E+08	3E+08	1E+09	1E+09	1E+09
G3V805	EKC/KEOPS complex subunit Tprkb OS=Rattus norvegicus GN=LOC100910911 PE=3 SV=1 - [G3V805_RAT]	0	2E+07	3E+07	5E+07	5E+07	5E+07
Q71UF4	Histone-binding protein RBBP7 OS=Rattus norvegicus GN=Rbbp7 PE=2 SV=1 - [RBBP7_RAT]	2E+08	8E+07	9E+07	3E+08	2E+08	2E+08
Q06645	ATP synthase F(0) complex subunit C1, mitochondrial OS=Rattus norvegicus GN=Atp5g1 PE=1 SV=1 - [AT5G1_RAT]	1E+07	9E+06	2E+07	7E+07	4E+07	1E+08
Q3B8P4	Decapping enzyme, scavenger OS=Rattus norvegicus GN=Dcps PE=2 SV=1 - [Q3B8P4_RAT]	6E+07	7E+07	3E+07	2E+08	1E+08	1E+08
Q06C60	BolA-like 1 (E. coli) (Predicted) OS=Rattus norvegicus GN=Bola1 PE=2 SV=1 - [Q06C60_RAT]	2E+07	2E+07	2E+07	6E+07	6E+07	5E+07
F1LXI9	Uncharacterized protein (Fragment) OS=Rattus norvegicus PE=4 SV=2 - [F1LXI9_RAT]	3E+09	2E+09	1E+09	8E+09	6E+09	5E+09
G3V6I5	Protein Dnaja3 OS=Rattus norvegicus GN=Dnaja3 PE=3 SV=1 - [G3V6I5_RAT]	3E+08	2E+08	2E+08	5E+08	4E+08	4E+08
D3ZW08	Adenylosuccinate lyase (Predicted) OS=Rattus norvegicus GN=Adsl PE=4 SV=1 - [D3ZW08_RAT]	2E+08	1E+08	1E+08	3E+08	3E+08	2E+08
G3V7K6	Single-stranded DNA-binding protein OS=Rattus norvegicus GN=Ssbp1 PE=3 SV=1 - [G3V7K6_RAT]	4E+07	6E+07	1E+07	1E+08	1E+08	1E+08
Q5M823	NudC domain-containing protein 2 OS=Rattus norvegicus GN=Nudcd2 PE=2 SV=1 - [NUDC2_RAT]	0	5E+07	7E+07	2E+08	2E+08	1E+08
D3ZPR0	Chromosome segregation 1-like (S. cerevisiae) (Predicted) OS=Rattus norvegicus GN=Cse1l PE=4 SV=1 - [D3ZPR0_RAT]	2E+08	2E+08	1E+08	3E+08	3E+08	2E+08
P51869	Cytochrome P450 4F4 OS=Rattus norvegicus GN=Cyp4f4 PE=2 SV=1 - [CP4F4_RAT]	6E+08	2E+08	2E+08	8E+08	8E+08	1E+09
P62994	Growth factor receptor-bound protein 2 OS=Rattus norvegicus GN=Grb2 PE=1 SV=1 - [GRB2_RAT]	4E+07	3E+07	0	2E+08	6E+07	1E+08
D3ZWS2	Protein 1600014C10Rik OS=Rattus norvegicus GN=LOC690000 PE=4 SV=1 - [D3ZWS2_RAT]	0	7E+07	8E+07	2E+08	2E+08	1E+08
P37397	Calponin-3 OS=Rattus norvegicus GN=Cnn3 PE=1 SV=1 - [CNN3_RAT]	7E+07	4E+07	7E+07	2E+08	2E+08	2E+08
P36970-2	Isoform Cytoplasmic of Phospholipid hydroperoxide glutathione peroxidase, mitochondrial OS=Rattus norvegicus GN=Gpx4 - [GPX41_RAT]	1E+08	2E+08	2E+08	4E+08	5E+08	4E+08
D4ADD7	Glutaredoxin 5 homolog (S. cerevisiae) (Predicted), isoform CRA_b OS=Rattus norvegicus GN=Glrx5 PE=4 SV=1 - [D4ADD7_RAT]	3E+08	4E+08	7E+07	1E+09	8E+08	8E+08
Q9QZH8	Arylacetamide deacetylase OS=Rattus norvegicus GN=Aadac PE=2 SV=3 - [AAAD_RAT]	3E+08	3E+08	2E+08	5E+08	6E+08	6E+08
D3ZZV1	Mitochondrial import inner membrane translocase subunit TIM16 OS=Rattus norvegicus GN=Pam16 PE=4 SV=1 - [D3ZZV1_RAT]	0	1E+08	2E+08	4E+08	4E+08	4E+08
B2RZ79	Iron-sulfur cluster scaffold homolog (E. coli) OS=Rattus norvegicus GN=Iscu PE=2 SV=1 - [B2RZ79_RAT]	0	2E+07	0	3E+07	4E+07	3E+07
D3ZD23	ATP-binding cassette, sub-family E (OABP), member 1 OS=Rattus norvegicus GN=Abce1 PE=3 SV=1 - [D3ZD23_RAT]	2E+08	2E+08	2E+08	4E+08	3E+08	4E+08
D4A6X7	Protein Ppcs OS=Rattus norvegicus GN=Ppcs PE=4 SV=1 - [D4A6X7_RAT]	1E+08	1E+08	8E+07	2E+08	2E+08	2E+08
P80386	5'-AMP-activated protein kinase subunit beta-1 OS=Rattus norvegicus GN=Prkab1 PE=1 SV=4 - [AAKB1_RAT]	9E+07	7E+07	3E+07	2E+08	3E+08	2E+08
A9UMV7	Protein LOC686442 OS=Rattus norvegicus GN=LOC686442 PE=4 SV=1 - [A9UMV7_RAT]	2E+08	1E+08	3E+08	6E+08	5E+08	6E+08
Q6AY09	Heterogeneous nuclear ribonucleoprotein H2 OS=Rattus norvegicus GN=Hnrnph2 PE=1 SV=1 - [HNRH2_RAT]	4E+08	2E+08	1E+08	7E+08	6E+08	7E+08
F1LRT5	Bis(5'-adenosyl)-triphosphatase OS=Rattus norvegicus GN=Fhit PE=4 SV=1 - [F1LRT5_RAT]	0	0	5E+07	1E+08	8E+07	6E+07
Q6TUG0	DnaJ homolog subfamily B member 11 OS=Rattus norvegicus GN=Dnajb11 PE=2 SV=1 - [DJB11_RAT]	4E+08	4E+08	9E+07	9E+08	9E+08	7E+08
Q64648	Cytochrome P450 2C12, female-specific OS=Rattus norvegicus GN=LOC100361492 PE=2 SV=1 - [Q64648_RAT]	2E+09	2E+09	7E+08	4E+09	3E+09	2E+09
Q5XIV1	Phosphoglycerate kinase OS=Rattus norvegicus GN=Pgk2 PE=2 SV=1 - [Q5XIV1_RAT]	5E+09	5E+09	2E+09	9E+09	7E+09	6E+09
O55004	Ribonuclease 4 OS=Rattus norvegicus GN=Rnase4 PE=1 SV=1 - [RNAS4_RAT]	6E+07	3E+07	6E+07	1E+08	1E+08	1E+08
I6L9G6	Protein Tardbp OS=Rattus norvegicus GN=Tardbp PE=2 SV=1 - [I6L9G6_RAT]	4E+07	1E+08	0	7E+08	4E+08	5E+08
F1LSA2	Cytochrome P450 2B2 OS=Rattus norvegicus GN=Cyp2b2 PE=3 SV=2 - [F1LSA2_RAT]	2E+09	2E+09	1E+09	4E+09	3E+09	6E+09
D3ZC69	Protein RGD1563668 OS=Rattus norvegicus GN=RGD1563668 PE=4 SV=2 - [D3ZC69_RAT]	1E+07	0	0	1E+08	2E+08	1E+08
Q9EQS0	Transaldolase OS=Rattus norvegicus GN=Taldo1 PE=1 SV=2 - [TALDO_RAT]	1E+09	4E+08	4E+08	2E+09	1E+09	2E+09
F7EUB6	Fibrinogen alpha chain OS=Rattus norvegicus GN=Fga PE=4 SV=1 - [F7EUB6_RAT]	6E+08	6E+08	4E+08	9E+08	1E+09	1E+09
P01048	T-kininogen 1 OS=Rattus norvegicus GN=Map1 PE=1 SV=2 - [KNT1_RAT]	2E+08	1E+08	2E+08	4E+08	6E+08	5E+08
P35427	60S ribosomal protein L13a OS=Rattus norvegicus GN=Rpl13a PE=1 SV=2 - [RL13A_RAT]	2E+08	2E+08	2E+08	5E+08	4E+08	5E+08
D3ZJX5	Protein LOC687295 OS=Rattus norvegicus GN=LOC687295 PE=4 SV=2 - [D3ZJX5_RAT]	2E+08	1E+08	6E+07	4E+08	4E+08	3E+08
P20759	Ig gamma-1 chain C region OS=Rattus norvegicus PE=1 SV=1 - [IGHG1_RAT]	3E+08	3E+08	2E+08	1E+08	1E+08	1E+08
F7DLY1	EPS8-like 2 (Predicted), isoform CRA_d OS=Rattus norvegicus GN=Eps8l2 PE=1 SV=1 - [F7DLY1_RAT]	1E+08	7E+07	9E+07	2E+08	2E+08	1E+08
P15978	Class I histocompatibility antigen, Non-RT1.A alpha-1 chain OS=Rattus norvegicus GN=RT1-Aw2 PE=1 SV=1 - [HA11_RAT]	3E+08	2E+08	2E+08	7E+08	6E+08	4E+08
Q9Z1W6-4	Isoform 4 of Protein LYRIC OS=Rattus norvegicus GN=Mtdh - [LYRIC_RAT]	2E+08	1E+08	7E+07	3E+08	3E+08	3E+08
O35263	Platelet-activating factor acetylhydrolase IB subunit gamma OS=Rattus norvegicus GN=Pafah1b3 PE=2 SV=1 - [PA1B3_RAT]	2E+07	0	2E+07	1E+08	4E+07	1E+08
Q641X8	Eukaryotic translation initiation factor 3 subunit E OS=Rattus norvegicus GN=Eif3e PE=2 SV=1 - [EIF3E_RAT]	1E+08	9E+07	6E+07	2E+08	2E+08	2E+08

Q5XIC6	Proteasome (Prosome, macropain) 26S subunit, non-ATPase, 12 OS=Rattus norvegicus GN=Psmd12 PE=2 SV=1 - [Q5XIC6_RAT]	1E+08	9E+07	1E+08	1E+08	2E+08	2E+08
D4A565	NADH dehydrogenase (Ubiquinone) 1 beta subcomplex, 5 (Predicted), isoform CRA_b OS=Rattus norvegicus GN=Ndufb5 PE=4 SV=1 - [D4A565_RAT]	6E+07	8E+07	7E+07	2E+08	3E+08	2E+08
E9PSZ7	Protein Cyp2t1 OS=Rattus norvegicus GN=Cyp2t1 PE=3 SV=1 - [E9PSZ7_RAT]	1E+08	8E+07	6E+07	4E+08	4E+08	2E+08
B2RZD5	Protein LOC688712 OS=Rattus norvegicus GN=Rpl22l1 PE=2 SV=1 - [B2RZD5_RAT]	5E+07	3E+07	0	8E+07	2E+08	2E+08
Q5U2V4	Phospholipase B-like 1 OS=Rattus norvegicus GN=Plbd1 PE=2 SV=1 - [PLBL1_RAT]	4E+08	4E+08	2E+08	7E+08	7E+08	5E+08
P61515	Putative 60S ribosomal protein L37a OS=Rattus norvegicus GN=Rpl37a-ps1 PE=5 SV=2 - [RL37P_RAT]	6E+08	3E+08	5E+08	7E+08	1E+09	9E+08
B1WBY7	ER lipid raft associated 1 OS=Rattus norvegicus GN=Erlin1 PE=2 SV=1 - [B1WBY7_RAT]	0	7E+07	0	1E+08	1E+08	1E+08
P39052	Dynamin-2 OS=Rattus norvegicus GN=Dnm2 PE=1 SV=1 - [DYN2_RAT]	2E+08	3E+08	2E+08	3E+08	5E+08	4E+08
Q6P0K8	Junction plakoglobin OS=Rattus norvegicus GN=Jup PE=1 SV=1 - [PLAK_RAT]	3E+07	9E+07	1E+08	2E+08	3E+08	3E+08
G3V7Q6	Proteasome subunit beta type OS=Rattus norvegicus GN=Psmb5 PE=3 SV=1 - [G3V7Q6_RAT]	0	3E+07	0	1E+08	2E+08	1E+08
Q9JHL6	Carcinoembryonic antigen-related cell adhesion molecule 1 (Precursor) OS=Rattus norvegicus GN=Ceacam1 PE=2 SV=1 - [Q9JHL6_RAT]	2E+08	5E+08	2E+08	7E+08	8E+08	7E+08
D3ZU04	Uncharacterized protein OS=Rattus norvegicus GN=LOC102550734 PE=4 SV=1 - [D3ZU04_RAT]	4E+08	2E+08	4E+08	7E+08	1E+09	6E+08
Q1JU68	Eukaryotic translation initiation factor 3 subunit A OS=Rattus norvegicus GN=Eif3a PE=2 SV=2 - [EIF3A_RAT]	4E+08	6E+08	5E+08	8E+08	1E+09	9E+08
F1MAA3	Protein LOC100909464 OS=Rattus norvegicus GN=LOC100909464 PE=4 SV=1 - [F1MAA3_RAT]	0	3E+07	4E+07	8E+07	1E+08	1E+08
M0R6I3	Uncharacterized protein (Fragment) OS=Rattus norvegicus PE=3 SV=1 - [M0R6I3_RAT]	9E+08	2E+09	2E+09	4E+09	4E+09	3E+09
B5DEL7	Protein LOC100361694 OS=Rattus norvegicus GN=Sec61g PE=3 SV=1 - [B5DEL7_RAT]	0	0	5E+07	2E+08	2E+08	1E+08
B2GV73	Actin related protein 2/3 complex, subunit 3 OS=Rattus norvegicus GN=Arpc3 PE=2 SV=1 - [B2GV73_RAT]	1E+08	2E+08	1E+08	4E+08	4E+08	3E+08
P26453-2	Isoform 2 of Basigin OS=Rattus norvegicus GN=Bsg - [BASI_RAT]	8E+08	6E+08	4E+08	1E+09	1E+09	1E+09
D3ZE29	Uncharacterized protein (Fragment) OS=Rattus norvegicus PE=3 SV=2 - [D3ZE29_RAT]	1E+09	2E+09	9E+08	2E+09	2E+09	3E+09
D4A3V2	NADH dehydrogenase [ubiquinone] 1 alpha subcomplex subunit 6 OS=Rattus norvegicus GN=Ndufa6 PE=3 SV=1 - [D4A3V2_RAT]	1E+08	9E+07	3E+07	5E+08	3E+08	4E+08
D4AC36	Eukaryotic translation initiation factor 3, subunit 5 (Epsilon) (Predicted), isoform CRA_a OS=Rattus norvegicus GN=Eif3f PE=4 SV=2 - [D4AC36_RAT]	3E+08	2E+08	1E+08	4E+08	3E+08	4E+08
Q99J82	Integrin-linked protein kinase OS=Rattus norvegicus GN=Ilk PE=2 SV=1 - [ILK_RAT]	9E+07	1E+08	8E+07	2E+08	2E+08	2E+08
P35280	Ras-related protein Rab-8A OS=Rattus norvegicus GN=Rab8a PE=1 SV=2 - [RAB8A_RAT]	7E+08	7E+08	5E+08	9E+08	1E+09	1E+09
D3ZZA8	Protein Sec24a OS=Rattus norvegicus GN=Sec24a PE=4 SV=1 - [D3ZZA8_RAT]	2E+08	1E+08	9E+07	2E+08	3E+08	4E+08
Q63707	Dihydroorotate dehydrogenase (quinone), mitochondrial OS=Rattus norvegicus GN=Dhodh PE=1 SV=1 - [PYRD_RAT]	5E+07	0	0	1E+08	2E+08	1E+08
R9PXR4	Mitochondrial import receptor subunit TOM70 OS=Rattus norvegicus GN=Tomm70a PE=1 SV=1 - [R9PXR4_RAT]	7E+07	1E+08	5E+07	2E+08	3E+08	3E+08
Q2TA68	Dynamin-like 120 kDa protein, mitochondrial OS=Rattus norvegicus GN=Opa1 PE=1 SV=1 - [OPA1_RAT]	1E+08	1E+08	8E+07	2E+08	3E+08	2E+08
D3ZIY0	Uncharacterized protein OS=Rattus norvegicus PE=3 SV=1 - [D3ZIY0_RAT]	8E+09	7E+09	3E+09	1E+10	1E+10	1E+10
Q75WE7	von Willebrand factor A domain-containing protein 5A OS=Rattus norvegicus GN=Vwa5a PE=2 SV=1 - [VWA5A_RAT]	2E+08	3E+08	3E+08	4E+08	5E+08	4E+08
B5DEG8	LOC685144 protein OS=Rattus norvegicus GN=Sec24c PE=2 SV=1 - [B5DEG8_RAT]	4E+08	4E+08	4E+08	6E+08	8E+08	7E+08
P32198	Carnitine O-palmitoyltransferase 1, liver isoform OS=Rattus norvegicus GN=Cpt1a PE=1 SV=2 - [CPT1A_RAT]	3E+07	1E+08	5E+07	3E+08	4E+08	4E+08
D3ZPF2	Protein Mcat OS=Rattus norvegicus GN=Mcat PE=4 SV=1 - [D3ZPF2_RAT]	7E+07	4E+07	4E+07	2E+08	2E+08	1E+08
F1LY19	Protein Upf1 OS=Rattus norvegicus GN=Upf1 PE=4 SV=2 - [F1LY19_RAT]	1E+08	2E+08	2E+08	3E+08	4E+08	3E+08
G3V9Z3	Amine oxidase [flavin-containing] A OS=Rattus norvegicus GN=Maoa PE=4 SV=1 - [G3V9Z3_RAT]	3E+08	3E+08	2E+08	4E+08	5E+08	6E+08
Q9QX79	Fetuin-B OS=Rattus norvegicus GN=Fetub PE=2 SV=2 - [FETUB_RAT]	3E+08	3E+08	3E+08	5E+08	6E+08	5E+08
Q99MI5	Protein LOC100912604 OS=Rattus norvegicus GN=LOC100364487 PE=2 SV=1 - [Q99MI5_RAT]	1E+08	9E+07	1E+08	2E+08	2E+08	2E+08
B2GV28	Cyp2b1 protein OS=Rattus norvegicus GN=Cyp2b1 PE=2 SV=1 - [B2GV28_RAT]	2E+09	2E+09	1E+09	4E+09	3E+09	6E+09
Q4KM86	Gamma-glutamylaminecyclotransferase OS=Rattus norvegicus GN=Ggact PE=2 SV=1 - [GGACT_RAT]	3E+07	0	1E+08	2E+08	2E+08	2E+08
Q9ES53	Ubiquitin fusion degradation protein 1 homolog OS=Rattus norvegicus GN=Ufd1l PE=1 SV=1 - [UFD1_RAT]	0	1E+08	0	2E+08	2E+08	3E+08
Q6AY21	GTPase activating protein (SH3 domain) binding protein 2 OS=Rattus norvegicus GN=G3bp2 PE=2 SV=1 - [Q6AY21_RAT]	0	6E+07	0	1E+08	7E+07	2E+08
B0BN18	Prefoldin subunit 2 OS=Rattus norvegicus GN=Pfdn2 PE=2 SV=1 - [PFD2_RAT]	7E+07	8E+07	1E+08	3E+08	3E+08	1E+08
P17136	Small nuclear ribonucleoprotein-associated protein B (Fragment) OS=Rattus norvegicus GN=Snrpb PE=2 SV=1 - [RSMB_RAT]	6E+07	3E+07	5E+07	2E+08	2E+08	1E+08
Q66H61	Glutaminyl-tRNA synthetase OS=Rattus norvegicus GN=RGD1562301 PE=2 SV=1 - [Q66H61_RAT]	1E+08	2E+08	2E+08	3E+08	3E+08	3E+08
D3ZUQ2	Protein Naa10 OS=Rattus norvegicus GN=Naa10 PE=4 SV=1 - [D3ZUQ2_RAT]	0	1E+07	0	5E+07	5E+07	5E+07
D3ZUM4	Beta-galactosidase OS=Rattus norvegicus GN=Glb1 PE=1 SV=1 - [D3ZUM4_RAT]	2E+08	2E+08	1E+08	4E+08	4E+08	4E+08
P09895	60S ribosomal protein L5 OS=Rattus norvegicus GN=Rpl5 PE=1 SV=3 - [RL5_RAT]	3E+08	1E+08	2E+08	4E+08	7E+08	5E+08
F1M8Y4	Protein Deptor OS=Rattus norvegicus GN=Deptor PE=4 SV=2 - [F1M8Y4_RAT]	0	2E+07	5E+07	7E+07	7E+07	6E+07
Q6AYF8	Protein Serpinb9 OS=Rattus norvegicus GN=Serpinb9 PE=2 SV=1 - [Q6AYF8_RAT]	2E+08	5E+07	2E+07	3E+08	2E+08	2E+08
P61314	60S ribosomal protein L15 OS=Rattus norvegicus GN=Rpl15 PE=1 SV=2 - [RL15_RAT]	5E+08	4E+08	2E+08	1E+09	8E+08	1E+09
O35543	Hematopoietic prostaglandin D synthase OS=Rattus norvegicus GN=Hpgds PE=1 SV=3 - [HPGDS_RAT]	0	5E+07	0	1E+08	9E+07	1E+08
Q68FT1	Ubiquinone biosynthesis protein COQ9, mitochondrial OS=Rattus norvegicus GN=Coq9 PE=2 SV=2 - [COQ9_RAT]	2E+08	3E+08	2E+08	5E+08	3E+08	3E+08
F1LU48	Protein Ergic1 (Fragment) OS=Rattus norvegicus GN=Ergic1 PE=4 SV=1 - [F1LU48_RAT]	9E+07	2E+07	3E+07	1E+08	1E+08	2E+08
D3ZXP7	Actin-related protein 2/3 complex subunit 1A OS=Rattus norvegicus GN=Arpc1a PE=4 SV=2 - [D3ZXP7_RAT]	9E+07	7E+07	0	2E+08	1E+08	2E+08
Q3T1L0	Aldehyde dehydrogenase family 16 member A1 OS=Rattus norvegicus GN=Aldh16a1 PE=2 SV=1 - [A16A1_RAT]	3E+08	2E+08	3E+08	5E+08	4E+08	3E+08
Q99068	Alpha-2-macroglobulin receptor-associated protein OS=Rattus norvegicus GN=Lrpap1 PE=1 SV=2 - [AMRP_RAT]	1E+08	1E+08	8E+07	4E+08	4E+08	2E+08
Q9JHY1	Junctional adhesion molecule A OS=Rattus norvegicus GN=F11r PE=2 SV=1 - [JAM1_RAT]	1E+08	9E+07	3E+07	3E+08	5E+08	3E+08

B2GUZ6	Protein Rtn4ip1 OS=Rattus norvegicus GN=Rtn4ip1 PE=2 SV=1 - [B2GUZ6_RAT]	1E+08	1E+08	0	4E+08	3E+08	2E+08
D3ZQT0	Uncharacterized protein OS=Rattus norvegicus PE=3 SV=1 - [D3ZQT0_RAT]	6E+08	2E+09	1E+09	4E+09	3E+09	3E+09
P38656	Lupus La protein homolog OS=Rattus norvegicus GN=Ssb PE=2 SV=1 - [LA_RAT]	6E+07	6E+07	4E+07	1E+08	2E+08	1E+08
P55009	Allograft inflammatory factor 1 OS=Rattus norvegicus GN=Aif1 PE=2 SV=1 - [AIF1_RAT]	0	2E+07	0	1E+08	1E+08	8E+07
Q6P685	Eukaryotic translation initiation factor 2, subunit 2 (Beta) OS=Rattus norvegicus GN=Eif2s2 PE=2 SV=1 - [Q6P685_RAT]	1E+07	0	0	9E+07	5E+07	2E+08
Q6P756	Adaptin ear-binding coat-associated protein 2 OS=Rattus norvegicus GN=Necap2 PE=1 SV=2 - [NECP2_RAT]	0	0	2E+07	4E+07	3E+07	6E+07
Q5U2X6	Coiled-coil domain-containing protein 47 OS=Rattus norvegicus GN=Ccdc47 PE=2 SV=1 - [CCD47_RAT]	1E+08	6E+07	5E+06	2E+08	1E+08	2E+08
P23764	Glutathione peroxidase 3 OS=Rattus norvegicus GN=Gpx3 PE=2 SV=2 - [GPX3_RAT]	6E+07	4E+07	4E+07	1E+08	1E+08	8E+07
D3ZN21	Protein RGD1309586 OS=Rattus norvegicus GN=RGD1309586 PE=3 SV=1 - [D3ZN21_RAT]	3E+08	5E+08	3E+08	6E+08	7E+08	7E+08
P08661	Mannose-binding protein C OS=Rattus norvegicus GN=Mbl2 PE=1 SV=2 - [MBL2_RAT]	1E+08	1E+08	0	4E+08	3E+08	4E+08
D3Z9D5	Protein Zbtb8os OS=Rattus norvegicus GN=Zbtb8os PE=4 SV=2 - [D3Z9D5_RAT]	0	6E+07	0	1E+08	1E+08	1E+08
F1LQW3	Protein Sfpq (Fragment) OS=Rattus norvegicus GN=Sfpq PE=4 SV=1 - [F1LQW3_RAT]	7E+07	2E+08	1E+08	4E+08	3E+08	3E+08
F1MA54	[Pyruvate dehydrogenase (acetyl-transferring)] kinase isozyme 1, mitochondrial OS=Rattus norvegicus GN=Pdk1 PE=4 SV=2 - [F1MA54_RAT]	8E+07	3E+07	6E+07	0	0	0
Q4KM69	COP9 (Constitutive photomorphogenic) homolog, subunit 5 (Arabidopsis thaliana) OS=Rattus norvegicus GN=Cops5 PE=2 SV=1 - [Q4KM69_RAT]	6E+07	2E+07	0	1E+08	7E+07	9E+07
Q6AXX6	Redox-regulatory protein FAM213A OS=Rattus norvegicus GN=Fam213a PE=1 SV=1 - [F213A_RAT]	1E+08	5E+07	0	2E+08	2E+08	2E+08
F1LQX9	Nuclear protein localization protein 4 homolog OS=Rattus norvegicus GN=Nploc4 PE=4 SV=2 - [F1LQX9_RAT]	6E+07	1E+08	1E+08	2E+08	2E+08	2E+08
D3ZK05	Gasdermin domain containing 1 (Predicted), isoform CRA_b OS=Rattus norvegicus GN=Gsdmd PE=4 SV=1 - [D3ZK05_RAT]	4E+07	7E+07	4E+07	1E+08	1E+08	1E+08
Q5XI95	Alcohol dehydrogenase 6 OS=Rattus norvegicus GN=Adh6 PE=2 SV=1 - [ADH6_RAT]	2E+08	3E+08	2E+08	6E+08	5E+08	4E+08
Q99NB7	Acyl-coenzyme A thioesterase 12 OS=Rattus norvegicus GN=Acot12 PE=1 SV=1 - [ACO12_RAT]	6E+07	1E+08	7E+07	6E+08	3E+08	3E+08
F7F469	Protein Irgm2 OS=Rattus norvegicus GN=Igtp PE=4 SV=1 - [F7F469_RAT]	1E+08	1E+08	6E+07	2E+08	2E+08	2E+08
P20611	Lysosomal acid phosphatase OS=Rattus norvegicus GN=Acp2 PE=1 SV=1 - [PPAL_RAT]	3E+08	2E+08	2E+08	4E+08	6E+08	7E+08
B2RYI2	Signal recognition particle subunit SRP68 OS=Rattus norvegicus GN=Srp68 PE=2 SV=1 - [B2RYI2_RAT]	1E+08	0	5E+07	2E+08	1E+08	2E+08
P62275	40S ribosomal protein S29 OS=Rattus norvegicus GN=Rps29 PE=1 SV=2 - [RS29_RAT]	3E+08	8E+07	0	2E+09	1E+09	2E+09
D4A233	Poly A binding protein, cytoplasmic 2 (Predicted) OS=Rattus norvegicus GN=Pabpc2 PE=4 SV=1 - [D4A233_RAT]	1E+09	9E+08	5E+08	1E+09	2E+09	2E+09
Q5XIF3	NADH dehydrogenase [ubiquinone] iron-sulfur protein 4, mitochondrial OS=Rattus norvegicus GN=Ndufs4 PE=1 SV=1 - [NDUS4_RAT]	9E+07	2E+08	1E+08	4E+08	5E+08	5E+08
P06765	Platelet factor 4 OS=Rattus norvegicus GN=Pf4 PE=1 SV=1 - [PLF4_RAT]	0	3E+07	0	6E+07	1E+08	7E+07
Q9WU82	Catenin beta-1 OS=Rattus norvegicus GN=Ctnnb1 PE=1 SV=1 - [CTNB1_RAT]	7E+07	9E+07	7E+07	2E+08	4E+08	4E+08
D3ZCH6	Protein Rnaset2 OS=Rattus norvegicus GN=Rnaset2 PE=3 SV=1 - [D3ZCH6_RAT]	3E+07	3E+07	2E+07	8E+07	7E+07	7E+07
Q5HZY0	UBX domain-containing protein 4 OS=Rattus norvegicus GN=Ubxn4 PE=2 SV=1 - [UBXN4_RAT]	1E+08	1E+08	3E+07	2E+08	3E+08	2E+08
F1LT58	Importin subunit alpha (Fragment) OS=Rattus norvegicus GN=Kpna6 PE=3 SV=2 - [F1LT58_RAT]	0	0	3E+07	5E+07	7E+07	9E+07
M0RBL5	Uncharacterized protein (Fragment) OS=Rattus norvegicus PE=3 SV=1 - [M0RBL5_RAT]	8E+09	6E+09	5E+09	1E+10	1E+10	1E+10
Q5M7A7	CB1 cannabinoid receptor-interacting protein 1 OS=Rattus norvegicus GN=Cnrip1 PE=1 SV=1 - [CNRP1_RAT]	0	1E+07	0	2E+07	3E+07	3E+07
B2GV24	E3 UFM1-protein ligase 1 OS=Rattus norvegicus GN=Ufl1 PE=2 SV=1 - [UFL1_RAT]	8E+07	2E+08	9E+07	2E+08	2E+08	2E+08
P53534	Glycogen phosphorylase, brain form (Fragment) OS=Rattus norvegicus GN=Pygb PE=1 SV=3 - [PYGB_RAT]	1E+09	3E+09	1E+09	3E+09	4E+09	4E+09
P51886	Lumican OS=Rattus norvegicus GN=Lum PE=2 SV=1 - [LUM_RAT]	8E+07	1E+08	8E+07	2E+08	3E+08	2E+08
F1M9N5	Ubiquitin conjugation factor E4 A OS=Rattus norvegicus GN=Ube4a PE=4 SV=1 - [F1M9N5_RAT]	9E+07	2E+08	1E+08	3E+08	3E+08	4E+08
P12001	60S ribosomal protein L18 OS=Rattus norvegicus GN=Rpl18 PE=2 SV=2 - [RL18_RAT]	2E+09	1E+09	1E+09	3E+09	2E+09	4E+09
E9PTD2	Phosphatidylinositol-binding clathrin assembly protein OS=Rattus norvegicus GN=Picalm PE=4 SV=2 - [E9PTD2_RAT]	2E+08	2E+08	2E+08	3E+08	3E+08	4E+08
A1A5R4	Acyl-CoA thioesterase 5 OS=Rattus norvegicus GN=Acot5 PE=2 SV=1 - [A1A5R4_RAT]	7E+08	1E+09	6E+08	2E+09	2E+09	1E+09
F1LRI5	Protein Gcn1l1 OS=Rattus norvegicus GN=Gcn1l1 PE=4 SV=2 - [F1LRI5_RAT]	5E+07	2E+08	1E+08	2E+08	2E+08	2E+08
P0C089	Phosphatidylglycerophosphatase and protein-tyrosine phosphatase 1 OS=Rattus norvegicus GN=Ptpmt1 PE=2 SV=1 - [PTPM1_RAT]	2E+07	3E+07	8E+06	6E+07	6E+07	6E+07
P55063	Heat shock 70 kDa protein 1-like OS=Rattus norvegicus GN=Hspa1l PE=2 SV=2 - [HS71L_RAT]	4E+09	5E+09	5E+09	1E+10	1E+10	1E+10
B5DEY0	Pls1 protein OS=Rattus norvegicus GN=Pls1 PE=2 SV=1 - [B5DEY0_RAT]	9E+08	8E+08	5E+08	1E+09	2E+09	1E+09
B0K034	Protein Dtd2 OS=Rattus norvegicus GN=Dtd2 PE=2 SV=1 - [B0K034_RAT]	0	3E+07	4E+07	7E+07	8E+07	8E+07
G3V9R8	Heterogeneous nuclear ribonucleoprotein C (C1/C2) OS=Rattus norvegicus GN=Hnrnpc PE=1 SV=2 - [G3V9R8_RAT]	5E+07	1E+08	5E+07	5E+08	2E+08	3E+08
D3ZFR9	Protein Rdh13 OS=Rattus norvegicus GN=Rdh13 PE=3 SV=1 - [D3ZFR9_RAT]	8E+06	2E+07	0	2E+07	2E+07	3E+07
M0R8B6	Protein Tubb1 OS=Rattus norvegicus GN=Tubb1 PE=3 SV=1 - [M0R8B6_RAT]	3E+09	3E+09	2E+09	5E+09	6E+09	4E+09
Q5U214	Protein Snrpa OS=Rattus norvegicus GN=Snrpa PE=2 SV=1 - [Q5U214_RAT]	0	1E+07	0	1E+08	1E+08	1E+08
D3ZF12	Signal peptidase complex subunit 3 OS=Rattus norvegicus GN=Spcs3 PE=4 SV=1 - [D3ZF12_RAT]	5E+07	4E+07	0	2E+08	2E+08	2E+08
Q9Z118	Polypyrimidine tract-binding protein 3 OS=Rattus norvegicus GN=Ptbp3 PE=2 SV=1 - [PTBP3_RAT]	0	2E+08	6E+07	2E+08	3E+08	3E+08
D3ZKG9	Clustered mitochondria protein homolog OS=Rattus norvegicus GN=Cluh PE=3 SV=2 - [D3ZKG9_RAT]	2E+08	2E+08	2E+08	3E+08	3E+08	3E+08
P07340	Sodium/potassium-transporting ATPase subunit beta-1 OS=Rattus norvegicus GN=Atp1b1 PE=1 SV=1 - [AT1B1_RAT]	4E+08	2E+08	1E+08	4E+08	6E+08	5E+08
D4AD15	Protein LOC100911431 OS=Rattus norvegicus GN=Eif4g1 PE=4 SV=1 - [D4AD15_RAT]	1E+08	1E+08	1E+08	2E+08	2E+08	2E+08
M0RCS2	Protein Tbl2 OS=Rattus norvegicus GN=Tbl2 PE=4 SV=1 - [M0RCS2_RAT]	1E+08	6E+07	0	2E+08	2E+08	2E+08
D3ZH98	Uncharacterized protein OS=Rattus norvegicus PE=3 SV=1 - [D3ZH98_RAT]	3E+08	2E+09	2E+09	3E+09	4E+09	3E+09
B5DFC8	Eukaryotic translation initiation factor 3 subunit C OS=Rattus norvegicus GN=Eif3c PE=2 SV=1 - [EIF3C_RAT]	1E+08	1E+08	2E+08	4E+08	2E+08	3E+08

Q6AZ50	Ubiquitin-like-conjugating enzyme ATG3 OS=Rattus norvegicus GN=Atg3 PE=2 SV=1 - [ATG3_RAT]	3E+07	3E+07	3E+07	1E+08	9E+07	7E+07
Q5XIE0	Acidic leucine-rich nuclear phosphoprotein 32 family member E OS=Rattus norvegicus GN=Anp32e PE=2 SV=1 - [AN32E_RAT]	1E+07	3E+07	0	7E+07	7E+07	8E+07
B5DEQ4	Protein Snrpb2 OS=Rattus norvegicus GN=Snrpb2 PE=2 SV=1 - [B5DEQ4_RAT]	0	1E+07	0	9E+07	7E+07	9E+07
F1LM66	Protein Eftud2 OS=Rattus norvegicus GN=Eftud2 PE=4 SV=1 - [F1LM66_RAT]	5E+07	0	0	9E+07	2E+08	2E+08
Q641Z7	Acid sphingomyelinase-like phosphodiesterase 3a OS=Rattus norvegicus GN=Smpdl3a PE=2 SV=1 - [ASM3A_RAT]	1E+08	7E+07	9E+07	2E+08	2E+08	2E+08
D3ZFX4	Phosphoglucomutase 3 (Predicted), isoform CRA_a OS=Rattus norvegicus GN=Pgm3 PE=3 SV=1 - [D3ZFX4_RAT]	7E+07	8E+07	6E+07	9E+07	1E+08	1E+08
D4A7U6	LSM3 homolog, U6 small nuclear RNA associated (S. cerevisiae) (Predicted) OS=Rattus norvegicus GN=Lsm3 PE=4 SV=1 - [D4A7U6_RAT]	0	4E+07	6E+07	1E+08	2E+08	1E+08
Q5RJR2	Twinfilin-1 OS=Rattus norvegicus GN=Twf1 PE=2 SV=1 - [TWF1_RAT]	6E+07	3E+07	4E+07	1E+08	1E+08	1E+08
A9UMV9	Ndufa7 protein OS=Rattus norvegicus GN=Ndufa7l PE=4 SV=1 - [A9UMV9_RAT]	0	1E+08	9E+07	4E+08	5E+08	5E+08
G3V8V3	Phosphorylase OS=Rattus norvegicus GN=Pygm PE=3 SV=1 - [G3V8V3_RAT]	2E+09	4E+09	2E+09	4E+09	4E+09	5E+09
A2RRT9	Cytochrome P450 4V2 OS=Rattus norvegicus GN=Cyp4v2 PE=2 SV=1 - [CP4V2_RAT]	8E+07	0	0	2E+08	2E+08	2E+08
F1LX07	Protein Slc25a12 (Fragment) OS=Rattus norvegicus GN=Slc25a12 PE=3 SV=2 - [F1LX07_RAT]	9E+08	9E+08	8E+08	2E+09	2E+09	2E+09
P80385	5'-AMP-activated protein kinase subunit gamma-1 OS=Rattus norvegicus GN=Prkag1 PE=1 SV=3 - [AAKG1_RAT]	4E+07	2E+07	3E+07	5E+07	8E+07	7E+07
G3V7T6	Protein Sf3b1 OS=Rattus norvegicus GN=Sf3b1 PE=4 SV=1 - [G3V7T6_RAT]	5E+07	0	3E+07	2E+08	2E+08	2E+08
F7ELI0	Protein Drg1 OS=Rattus norvegicus GN=Drg1 PE=4 SV=1 - [F7ELI0_RAT]	3E+07	0	0	1E+08	1E+08	8E+07
G3V7L0	Adrenodoxin, mitochondrial OS=Rattus norvegicus GN=Fdx1 PE=4 SV=1 - [G3V7L0_RAT]	0	2E+07	1E+07	7E+07	9E+07	5E+07
P42667	Signal peptidase complex catalytic subunit SEC11A OS=Rattus norvegicus GN=Sec11a PE=2 SV=1 - [SC11A_RAT]	7E+07	3E+07	6E+07	1E+08	1E+08	9E+07
Q63377	Sodium/potassium-transporting ATPase subunit beta-3 OS=Rattus norvegicus GN=Atp1b3 PE=2 SV=1 - [AT1B3_RAT]	2E+08	8E+07	2E+07	5E+08	4E+08	3E+08
D3ZA85	Histone cell cycle regulation defective interacting protein 5 (Predicted), isoform CRA_a OS=Rattus norvegicus GN=Nfu1 PE=4 SV=1 - [D3ZA85_RAT]	5E+07	4E+07	0	1E+08	1E+08	2E+08
D3ZUY8	Adaptor protein complex AP-2, alpha 1 subunit (Predicted) OS=Rattus norvegicus GN=Ap2a1 PE=1 SV=1 - [D3ZUY8_RAT]	8E+07	2E+08	2E+08	4E+08	5E+08	5E+08
P97840-2	Isoform Short of Galectin-9 OS=Rattus norvegicus GN=Lgals9 - [LEG9_RAT]	0	3E+07	2E+07	7E+07	7E+07	9E+07
F1LM03	Cytochrome P450 2C55 OS=Rattus norvegicus GN=Cyp2c24 PE=3 SV=2 - [F1LM03_RAT]	1E+09	1E+09	7E+08	3E+09	2E+09	2E+09
P61805	Dolichyl-diphosphooligosaccharide--protein glycosyltransferase subunit DAD1 OS=Rattus norvegicus GN=Dad1 PE=3 SV=3 - [DAD1_RAT]	7E+07	2E+08	1E+08	4E+08	5E+08	5E+08
Q63042	FAD-linked sulfhydryl oxidase ALR OS=Rattus norvegicus GN=Gfer PE=1 SV=2 - [ALR_RAT]	5E+07	4E+07	7E+07	8E+07	1E+08	2E+08
Q9EPB1	Dipeptidyl peptidase 2 OS=Rattus norvegicus GN=Dpp7 PE=1 SV=1 - [DPP2_RAT]	8E+07	7E+07	1E+08	1E+08	1E+08	1E+08
B3GNI6-3	Isoform 3 of Septin-11 OS=Rattus norvegicus GN=Sept11 - [SEP11_RAT]	2E+08	2E+08	8E+07	3E+08	4E+08	3E+08
D4AB06	Protein Haus5 OS=Rattus norvegicus GN=Haus5 PE=4 SV=2 - [D4AB06_RAT]	2E+08	1E+08	9E+07	4E+08	5E+08	3E+08
D3ZVL1	Protein LOC100912115 OS=Rattus norvegicus GN=LOC100912115 PE=4 SV=2 - [D3ZVL1_RAT]	6E+07	0	0	2E+08	1E+08	2E+08
M0R961	Far upstream element-binding protein 2 OS=Rattus norvegicus GN=Khsrp PE=4 SV=1 - [M0R961_RAT]	4E+08	1E+08	2E+08	5E+08	5E+08	7E+08
F1M5K4	Glyceraldehyde-3-phosphate dehydrogenase (Fragment) OS=Rattus norvegicus PE=3 SV=2 - [F1M5K4_RAT]	1E+10	1E+10	1E+10	3E+10	3E+10	4E+10
D3ZZZ9	Catenin (Cadherin associated protein), delta 1 (Predicted), isoform CRA_a OS=Rattus norvegicus GN=Ctnnd1 PE=1 SV=1 - [D3ZZZ9_RAT]	4E+07	1E+08	6E+07	5E+08	9E+08	5E+08
P19939	Apolipoprotein C-I OS=Rattus norvegicus GN=Apoc1 PE=2 SV=1 - [APOC1_RAT]	2E+07	0	0	2E+08	2E+08	2E+08
P63322	Ras-related protein Ral-A OS=Rattus norvegicus GN=Rala PE=1 SV=1 - [RALA_RAT]	2E+07	1E+07	0	9E+07	7E+07	5E+07
B5DEQ3	Probable Xaa-Pro aminopeptidase 3 OS=Rattus norvegicus GN=Xpnpep3 PE=1 SV=1 - [XPP3_RAT]	1E+08	7E+07	1E+08	2E+08	2E+08	2E+08
D3Z9J8	Sodium-coupled neutral amino acid transporter 2 OS=Rattus norvegicus GN=Sat2 PE=4 SV=1 - [D3Z9J8_RAT]	0	0	9E+06	1E+08	3E+07	8E+07
F1M4C0	2'-5'-oligoadenylate synthase 1A OS=Rattus norvegicus GN=Oas1a PE=4 SV=2 - [F1M4C0_RAT]	3E+07	0	2E+07	1E+08	9E+07	1E+08
Q6AY59	Molybdopterin synthase catalytic subunit OS=Rattus norvegicus GN=Mocs2 PE=2 SV=1 - [MOC2B_RAT]	6E+07	3E+07	8E+07	2E+08	1E+08	1E+08
Q7TP54	Ab2-162 OS=Rattus norvegicus GN=Fam65b PE=2 SV=1 - [Q7TP54_RAT]	3E+09	2E+09	3E+09	9E+09	8E+09	5E+09
G3V9A4	2'-5'-oligoadenylate synthase 1A OS=Rattus norvegicus GN=Oas1a PE=4 SV=1 - [G3V9A4_RAT]	3E+07	0	4E+07	1E+08	9E+07	1E+08
Q63186	Translation initiation factor eIF-2B subunit delta OS=Rattus norvegicus GN=Eif2b4 PE=2 SV=1 - [EI2BD_RAT]	0	3E+07	0	5E+07	6E+07	5E+07
F1LLX8	Lysosome-associated membrane glycoprotein 2 OS=Rattus norvegicus GN=Lamp2 PE=4 SV=1 - [F1LLX8_RAT]	6E+08	7E+08	3E+08	1E+09	9E+08	1E+09
Q5RJQ4	NAD-dependent protein deacetylase sirtuin-2 OS=Rattus norvegicus GN=Sirt2 PE=1 SV=1 - [SIR2_RAT]	0	2E+07	0	5E+07	3E+07	3E+07
Q8VI04	Isoaspartyl peptidase/L-asparaginase OS=Rattus norvegicus GN=Asrgl1 PE=1 SV=1 - [ASGL1_RAT]	0	2E+07	0	3E+07	4E+07	4E+07
G3V7F6	Protein RGD1561590 OS=Rattus norvegicus GN=RGD1561590 PE=4 SV=1 - [G3V7F6_RAT]	0	2E+07	0	7E+07	7E+07	2E+08
D4A9D6	DEAH (Asp-Glu-Ala-His) box polypeptide 9 (Predicted) OS=Rattus norvegicus GN=Dhx9 PE=4 SV=1 - [D4A9D6_RAT]	1E+07	0	0	2E+08	1E+08	2E+08
Q9JK15	Arf-GAP with dual PH domain-containing protein 2 OS=Rattus norvegicus GN=Adap2 PE=1 SV=1 - [ADAP2_RAT]	1E+07	0	0	3E+07	3E+07	2E+07
D4AE41	RNA binding motif protein, X-linked-like-1 OS=Rattus norvegicus GN=Rbmxl1 PE=3 SV=1 - [RMXL1_RAT]	1E+08	0	5E+07	2E+08	3E+08	1E+08
Q9JMJ4	Pre-mRNA-processing factor 19 OS=Rattus norvegicus GN=Prpf19 PE=2 SV=2 - [PRP19_RAT]	1E+08	2E+08	8E+07	2E+08	4E+08	4E+08
O70513	Galectin-3-binding protein OS=Rattus norvegicus GN=Lgals3bp PE=1 SV=2 - [LG3BP_RAT]	3E+07	3E+07	4E+07	2E+08	2E+08	2E+08
Q6TUH9	Corticosteroid 11-beta-dehydrogenase isozyme 1 OS=Rattus norvegicus GN=Hsd11b1 PE=2 SV=1 - [Q6TUH9_RAT]	1E+09	2E+09	9E+08	3E+09	2E+09	3E+09
G3V6F5	ElaC homolog 2 (E. coli) OS=Rattus norvegicus GN=Elac2 PE=4 SV=1 - [G3V6F5_RAT]	4E+07	0	0	8E+07	1E+08	1E+08
G3V8Q8	Protein Sec23ip OS=Rattus norvegicus GN=Sec23ip PE=4 SV=1 - [G3V8Q8_RAT]	1E+08	1E+08	9E+07	2E+08	1E+08	2E+08
Q9QZR6-3	Isoform 3 of Septin-9 OS=Rattus norvegicus GN=Sept9 - [SEPT9_RAT]	1E+08	8E+07	1E+08	2E+08	3E+08	2E+08
P62634	Cellular nucleic acid-binding protein OS=Rattus norvegicus GN=Cnbp PE=2 SV=1 - [CNBP_RAT]	1E+08	1E+08	2E+08	4E+08	3E+08	2E+08
Q99JC6	Protein Tapbp OS=Rattus norvegicus GN=Tapbp PE=2 SV=1 - [Q99JC6_RAT]	0	6E+07	6E+07	1E+08	2E+08	2E+08
G3V9T9	Tyrosine-protein phosphatase non-receptor type OS=Rattus norvegicus GN=Ptpn6 PE=3 SV=1 - [G3V9T9_RAT]	0	0	4E+07	1E+08	6E+07	1E+08

Q5M7T6	ATPase, H+ transporting, lysosomal 38kDa, V0 subunit d1 OS=Rattus norvegicus GN=Atp6v0d1 PE=2 SV=1 - [Q5M7T6_RAT]	9E+07	6E+07	5E+07	3E+08	3E+08	1E+08
D3ZZN4	Protein LOC100362338 OS=Rattus norvegicus GN=LOC100362338 PE=4 SV=1 - [D3ZZN4_RAT]	0	1E+08	6E+07	5E+08	8E+08	5E+08
Q6P734	Plasma protease C1 inhibitor OS=Rattus norvegicus GN=Serping1 PE=2 SV=1 - [IC1_RAT]	6E+07	6E+07	8E+07	2E+08	2E+08	2E+08
E9PTI6	Protein Raly OS=Rattus norvegicus GN=Raly PE=4 SV=1 - [E9PTI6_RAT]	0	2E+07	0	2E+08	1E+08	1E+08
F1LMP7	Granulins OS=Rattus norvegicus GN=Grn PE=2 SV=1 - [F1LMP7_RAT]	6E+07	0	2E+08	3E+08	3E+08	2E+08
P62804	Histone H4 OS=Rattus norvegicus GN=Hist1h4b PE=1 SV=2 - [H4_RAT]	1E+08	1E+08	3E+08	5E+08	1E+09	5E+08
Q9JJH5-2	Isoform 1 of 6-phosphofructo-2-kinase/fructose-2,6-bisphosphatase 2 OS=Rattus norvegicus GN=Pfkfb2 - [F262_RAT]	0	4E+07	1E+08	4E+08	4E+08	4E+08
Q8CGU6	Nicastrin OS=Rattus norvegicus GN=Ncstn PE=1 SV=1 - [NICA_RAT]	8E+06	7E+07	4E+07	1E+08	1E+08	1E+08
Q62940	E3 ubiquitin-protein ligase NEDD4 OS=Rattus norvegicus GN=Nedd4 PE=1 SV=1 - [NEDD4_RAT]	6E+07	3E+07	6E+07	9E+07	9E+07	1E+08
Q63203	Low affinity immunoglobulin gamma Fc region receptor II OS=Rattus norvegicus GN=Fcgr2 PE=2 SV=1 - [FCGR2_RAT]	2E+08	1E+08	8E+07	3E+08	4E+08	3E+08
P08753	Guanine nucleotide-binding protein G(k) subunit alpha OS=Rattus norvegicus GN=Gnai3 PE=1 SV=3 - [GNAI3_RAT]	3E+08	3E+08	3E+08	6E+08	7E+08	5E+08
Q5U2R9	Protein Scfd2 OS=Rattus norvegicus GN=Scfd2 PE=2 SV=1 - [Q5U2R9_RAT]	1E+07	2E+07	2E+07	4E+07	3E+07	4E+07
F1LPG9	Protein Fam21c OS=Rattus norvegicus GN=Fam21c PE=4 SV=2 - [F1LPG9_RAT]	0	0	8E+06	2E+07	3E+07	2E+07
Q5XI29	Cleavage and polyadenylation specificity factor subunit 7 OS=Rattus norvegicus GN=Cpsf7 PE=2 SV=1 - [CPSF7_RAT]	0	9E+05	1E+06	2E+07	1E+07	1E+07
D3ZTR1	Protein Mrps17 OS=Rattus norvegicus GN=Mrps17 PE=4 SV=1 - [D3ZTR1_RAT]	2E+07	1E+07	0	4E+07	8E+07	4E+07
F1M2S3	Protein RGD1561118 (Fragment) OS=Rattus norvegicus GN=RGD1561118 PE=4 SV=1 - [F1M2S3_RAT]	8E+08	5E+08	3E+08	8E+08	1E+09	1E+09
E9PTU4	Myosin-11 OS=Rattus norvegicus GN=Myh11 PE=4 SV=2 - [E9PTU4_RAT]	5E+07	0	9E+07	2E+08	4E+08	3E+08
Q63210	Guanine nucleotide-binding protein subunit alpha-12 OS=Rattus norvegicus GN=Gna12 PE=1 SV=3 - [GNA12_RAT]	5E+08	3E+08	3E+08	6E+08	7E+08	5E+08
Q63921	Prostaglandin G/H synthase 1 OS=Rattus norvegicus GN=Ptgs1 PE=2 SV=2 - [PGH1_RAT]	4E+07	0	2E+07	1E+08	1E+08	1E+08
G3V8T9	Apoptosis regulator BAX OS=Rattus norvegicus GN=Bax PE=4 SV=1 - [G3V8T9_RAT]	0	1E+07	0	5E+07	6E+07	5E+07
F1M9N7	Arf-GAP domain and FG repeat-containing protein 1 OS=Rattus norvegicus GN=Agfg1 PE=4 SV=1 - [F1M9N7_RAT]	5E+06	0	5E+06	2E+07	1E+07	1E+07
F1LQP9	Protein Tnpo1 (Fragment) OS=Rattus norvegicus GN=Tnpo1 PE=4 SV=2 - [F1LQP9_RAT]	5E+07	7E+07	4E+07	9E+07	1E+08	8E+07
P43244	Matrin-3 OS=Rattus norvegicus GN=Matr3 PE=1 SV=2 - [MATR3_RAT]	1E+08	0	0	2E+08	2E+08	3E+08
Q568Z8	Ddx17 protein OS=Rattus norvegicus GN=Ddx17 PE=2 SV=1 - [Q568Z8_RAT]	9E+07	0	0	2E+08	2E+08	2E+08
O35821	Myb-binding protein 1A OS=Rattus norvegicus GN=Mybbp1a PE=2 SV=2 - [MBB1A_RAT]	3E+07	0	0	1E+08	9E+07	9E+07
F1M446	Protein AI314180 (Fragment) OS=Rattus norvegicus GN=AI314180 PE=4 SV=2 - [F1M446_RAT]	1E+07	0	2E+07	4E+07	5E+07	4E+07
Q63556	Serine protease inhibitor A3M (Fragment) OS=Rattus norvegicus GN=Serpina3m PE=2 SV=1 - [SPA3M_RAT]	5E+07	5E+08	5E+08	1E+09	1E+09	1E+09
G3V9Y1	Myosin, heavy polypeptide 10, non-muscle, isoform CRA_b OS=Rattus norvegicus GN=Myh10 PE=4 SV=1 - [G3V9Y1_RAT]	6E+07	0	0	2E+08	5E+08	3E+08
Q9JJ22-2	Isoform 2 of Endoplasmic reticulum aminopeptidase 1 OS=Rattus norvegicus GN=Erap1 - [ERAP1_RAT]	7E+06	1E+08	8E+07	2E+08	2E+08	2E+08
Q56R17	Importin subunit alpha OS=Rattus norvegicus GN=Kpna4 PE=2 SV=1 - [Q56R17_RAT]	5E+07	5E+07	4E+07	1E+08	1E+08	1E+08
Q6MG08	ATP-binding cassette sub-family F member 1 OS=Rattus norvegicus GN=Abcf1 PE=1 SV=1 - [ABCF1_RAT]	5E+07	7E+07	4E+07	1E+08	1E+08	1E+08
Q5RJS3	Family with sequence similarity 96, member A OS=Rattus norvegicus GN=Fam96a PE=2 SV=1 - [Q5RJS3_RAT]	0	1E+07	2E+07	3E+07	4E+07	3E+07
F1LMT9	Protein Trim47l OS=Rattus norvegicus GN=Trim47l PE=4 SV=1 - [F1LMT9_RAT]	3E+08	5E+09	0	9E+09	7E+09	1E+10
P62997	Transformer-2 protein homolog beta OS=Rattus norvegicus GN=Tra2b PE=1 SV=1 - [TRA2B_RAT]	8E+07	0	6E+07	1E+08	2E+08	2E+08
D3ZFQ8	Cytochrome c-1 (Predicted), isoform CRA_b OS=Rattus norvegicus GN=Cyc1 PE=4 SV=2 - [D3ZFQ8_RAT]	9E+08	9E+08	1E+09	2E+09	1E+09	1E+09
D4AB93	Uncharacterized protein OS=Rattus norvegicus PE=3 SV=1 - [D4AB93_RAT]	1E+07	1E+07	0	5E+07	3E+07	3E+07
F1LWK4	Afadin (Fragment) OS=Rattus norvegicus GN=Mllt4 PE=4 SV=2 - [F1LWK4_RAT]	0	0	3E+07	1E+08	4E+07	1E+08
D3ZM09	Protein Sars2 OS=Rattus norvegicus GN=Sars2 PE=3 SV=1 - [D3ZM09_RAT]	0	0	6E+07	3E+08	2E+08	2E+08
F7FC39	Protein Spta1 OS=Rattus norvegicus GN=Spta1 PE=1 SV=1 - [F7FC39_RAT]	2E+08	7E+07	1E+08	4E+08	2E+08	3E+08
Q9QUH3	Apolipoprotein A-V OS=Rattus norvegicus GN=Apoa5 PE=2 SV=1 - [APOA5_RAT]	7E+07	4E+07	0	1E+08	2E+08	1E+08
G3V7J9	Alcohol dehydrogenase class 4 mu/sigma chain OS=Rattus norvegicus GN=Adh7 PE=3 SV=1 - [G3V7J9_RAT]	1E+08	3E+06	1E+08	4E+08	4E+08	3E+08
F1LR71	Protein Aspscr1 OS=Rattus norvegicus GN=Aspscr1 PE=4 SV=2 - [F1LR71_RAT]	4E+07	2E+07	3E+07	6E+07	6E+07	6E+07
P55062	Bax inhibitor 1 OS=Rattus norvegicus GN=Tmbim6 PE=2 SV=2 - [BI1_RAT]	4E+07	4E+07	0	6E+07	8E+07	8E+07
G3V6G7	Polyribonucleotide nucleotidyltransferase 1 OS=Rattus norvegicus GN=Pnpt1 PE=4 SV=1 - [G3V6G7_RAT]	0	7E+07	3E+07	8E+07	1E+08	9E+07
Q9QUR2-2	Isoform 2 of Dynactin subunit 4 OS=Rattus norvegicus GN=Dctn4 - [DCTN4_RAT]	0	0	8E+07	2E+08	3E+08	3E+08
D3ZN76	Protein Sec16a OS=Rattus norvegicus GN=Sec16a PE=4 SV=1 - [D3ZN76_RAT]	5E+07	0	4E+07	7E+07	1E+08	1E+08
Q63648	Merlin (Fragment) OS=Rattus norvegicus GN=Nf2 PE=1 SV=1 - [MERL_RAT]	2E+08	2E+08	2E+08	3E+08	2E+08	3E+08
M0R762	Protein Smg1 OS=Rattus norvegicus GN=Smg1 PE=4 SV=1 - [M0R762_RAT]	5E+08	3E+08	1E+09	3E+09	4E+09	2E+09
D3ZGR6	Protein Ccdc138 OS=Rattus norvegicus GN=Ccdc138 PE=4 SV=1 - [D3ZGR6_RAT]	1E+08	8E+07	1E+08	2E+08	3E+08	3E+08
O35162	Heat shock 70 kDa protein 13 OS=Rattus norvegicus GN=Hspa13 PE=2 SV=2 - [HSP13_RAT]	8E+07	6E+07	5E+07	1E+08	1E+08	1E+08
P84903	Stromal interaction molecule 1 OS=Rattus norvegicus GN=Stim1 PE=1 SV=1 - [STIM1_RAT]	0	0	2E+07	8E+07	7E+07	8E+07
D3ZCG3	Protein Mon2 (Fragment) OS=Rattus norvegicus GN=Mon2 PE=4 SV=2 - [D3ZCG3_RAT]	0	1E+07	2E+07	5E+07	3E+07	5E+07
F1LP64-2	Isoform 2 of E3 ubiquitin-protein ligase TRIP12 OS=Rattus norvegicus GN=Trip12 - [TRIPC_RAT]	2E+06	0	0	3E+07	3E+07	1E+07
M0RDL0	Protein Prdm16 (Fragment) OS=Rattus norvegicus GN=Prdm16 PE=4 SV=1 - [M0RDL0_RAT]	4E+08	2E+08	2E+08	1E+09	1E+09	9E+08
D4A885	Protein Abca13 OS=Rattus norvegicus GN=Abca13 PE=4 SV=2 - [D4A885_RAT]	3E+07	6E+07	3E+07	1E+08	1E+08	8E+07
F1LPM1	Protocadherin Fat 2 OS=Rattus norvegicus GN=Fat2 PE=4 SV=2 - [F1LPM1_RAT]	4E+08	1E+08	2E+08	6E+08	9E+08	7E+08

Table S2 101 proteins in oxidation-reduction biological process.

ID	Gene Name
P32755	4-hydroxyphenylpyruvate dioxygenase(Hpd)
D4A253	Cytochrome P450, family 2, subfamily c, polypeptide 7-like(LOC100361547)
O35078	D-amino-acid oxidase(Dao)
B0BNJ4	ETHE1, persulfide dioxygenase(Ethe1)
P50398	GDP dissociation inhibitor 1(Gdi1)
D3ZVS2	L-2-hydroxyglutarate dehydrogenase(L2hgdh)
Q641Y2	NADH dehydrogenase (ubiquinone) Fe-S protein 2(Ndufs2)
D3ZG43	NADH dehydrogenase (ubiquinone) Fe-S protein 3(Ndufs3)
B0BNE6	NADH:ubiquinone oxidoreductase core subunit S8(Ndufs8)
F1LXA0	NADH:ubiquinone oxidoreductase subunit A12(Ndufa12)
D3ZS58	NADH:ubiquinone oxidoreductase subunit A2(Ndufa2)
B2RZD6	NADH:ubiquinone oxidoreductase subunit A4(Ndufa4)
D4A3V2	NADH:ubiquinone oxidoreductase subunit A6(Ndufa6)
Q7TP78	NADH:ubiquinone oxidoreductase subunit A8(Ndufa8)
Q5BK63	NADH:ubiquinone oxidoreductase subunit A9(Ndufa9)
D3ZLT1	NADH:ubiquinone oxidoreductase subunit B7(Ndufb7)
Q6PCU8	NADH:ubiquinone oxidoreductase subunit V3(Ndufv3)
B2RZ27	SH3 domain binding glutamate-rich protein like 3(Sh3bgrl3)
O70199, G3V6C4	UDP-glucose 6-dehydrogenase(Ugdh)
Q562C9	acireductone dioxygenase 1(Adi1)
F1LSP2	acyl-CoA dehydrogenase family, member 10(Acad10)
P15651	acyl-CoA dehydrogenase, C-2 to C-3 short chain(Acads)
P08503	acyl-CoA dehydrogenase, C-4 to C-12 straight chain(Acadm)
P70584	acyl-CoA dehydrogenase, short/branched chain(Acadsb)
Q5XI95	alcohol dehydrogenase 6 (class V)(Adh6)
G3V7J9	alcohol dehydrogenase 7 (class IV), mu or sigma polypeptide(Adh7)
Q4QQW3	alcohol dehydrogenase, iron containing, 1(Adhfe1)
Q3T1L0	aldehyde dehydrogenase 16 family, member A1(Aldh16a1)
D3ZXY4	aldehyde dehydrogenase 8 family, member A1(Aldh8a1)
Q9JLJ3	aldehyde dehydrogenase 9 family, member A1(Aldh9a1)
P51635	aldo-keto reductase family 1 member A1(Akr1a1)
Q8CG45	aldo-keto reductase family 7, member A2(Akr7a2)
Q9JM53	apoptosis inducing factor, mitochondria associated 1(Aifm1)
A3KN98	beta-carotene oxygenase 2(Bco2)
P35738	branched chain keto acid dehydrogenase E1 subunit beta(Bckdhb)
Q6UPE0	choline dehydrogenase(Chdh)
Q3B7D0	coproporphyrinogen oxidase(Cpox)
F1LR47, P05178	cytochrome P450, family 2, subfamily C, polypeptide 6, variant 1(Cyp2c6v1)
B2GV28	cytochrome P450, family 2, subfamily b, polypeptide 1(Cyp2b1)
F1LSA2	cytochrome P450, family 2, subfamily b, polypeptide 2(Cyp2b2)

Q64648	cytochrome P450, family 2, subfamily c, polypeptide 12(Cyp2c12)
P20814	cytochrome P450, family 2, subfamily c, polypeptide 13(Cyp2c13)
P19225	cytochrome P450, family 2, subfamily c, polypeptide 22(Cyp2c22)
F1LM03	cytochrome P450, family 2, subfamily c, polypeptide 55-like(LOC100361492)
P05182	cytochrome P450, family 2, subfamily e, polypeptide 1(Cyp2e1)
P51590	cytochrome P450, family 2, subfamily j, polypeptide 3(Cyp2j3)
E9PSZ7	cytochrome P450, family 2, subfamily t, polypeptide 1(Cyp2t1)
Q64581	cytochrome P450, family 3, subfamily a, polypeptide 18(Cyp3a18)
Q06884	cytochrome P450, family 3, subfamily a, polypeptide 23/polypeptide 1(Cyp3a23/3a1)
P08516	cytochrome P450, family 4, subfamily a, polypeptide 1(Cyp4a1)
P33274	cytochrome P450, family 4, subfamily f, polypeptide 1(Cyp4f1)
P51869	cytochrome P450, family 4, subfamily f, polypeptide 4(Cyp4f4)
P08683	cytochrome P450, subfamily 2, polypeptide 11(Cyp2c11)
P00388	cytochrome p450 oxidoreductase(Por)
Q8VID1	dehydrogenase/reductase 4(Dhrs4)
Q920D2	dihydrofolate reductase(Dhfr)
Q63707	dihydroorotate dehydrogenase (quinone)(Dhodh)
F1M891	dihydropyrimidine dehydrogenase(Dpyd)
Q63342, Q5RKL4	dimethylglycine dehydrogenase(Dmgdh)
Q9EQ76	flavin containing monooxygenase 3(Fmo3)
D4ADD7	glutaredoxin 5(Glrx5)
Q9ESH6	glutaredoxin(Glrx)
P24473	glutathione S-transferase kappa 1(Gstk1)
P04041	glutathione peroxidase 1(Gpx1)
P23764	glutathione peroxidase 3(Gpx3)
D3ZGY4	glyceraldehyde-3-phosphate dehydrogenase, pseudogene 2(Gapdh-ps2)
D4A5Q9	glycine decarboxylase(Gldc)
B0BN46	glyoxylate and hydroxypyruvate reductase(Grhpr)
Q63042	growth factor, augmenter of liver regeneration(Gfer)
P10867	gulonolactone (L-) oxidase(Gulo)
D4A7D7	hexose-6-phosphate dehydrogenase (glucose 1-dehydrogenase)(H6pd)
P27364	hydroxy-delta-5-steroid dehydrogenase, 3 beta- and steroid delta-isomerase 5(Hsd3b5)
Q60587	hydroxyacyl-CoA dehydrogenase/3-ketoacyl-CoA thiolase/enoyl-CoA hydratase (trifunctional protein), beta subunit(Hadhb)
Q6AYS8	hydroxysteroid (17-beta) dehydrogenase 11(Hsd17b11)
Q6TUH9	hydroxysteroid 11-beta dehydrogenase 1(Hsd11b1)
P04642	lactate dehydrogenase A(Ldha)
P08011	microsomal glutathione S-transferase 1(Mgst1)
G3V6I4	mitochondrial amidoxime reducing component 1(Marc1)
Q9Z311, F1LPY7	mitochondrial trans-2-enoyl-CoA reductase(Mecr)
G3V9Z3	monoamine oxidase A(Maoa)
P35704	peroxiredoxin 2(Prdx2)
G3V7I0	peroxiredoxin 3(Prdx3)
Q9Z0V5	peroxiredoxin 4(Prdx4)
P04176, Q6AYW2	phenylalanine hydroxylase(Pah)
F1MAR6	proline dehydrogenase 1(Prodh1)

Q5BK81	prostaglandin reductase 2(Ptgr2)
Q63921	prostaglandin-endoperoxide synthase 1(Ptgs1)
P11598	protein disulfide isomerase family A, member 3(Pdia3)
P61459	pterin-4 alpha-carbinolamine dehydratase 1(Pcbd1)
P26284	pyruvate dehydrogenase (lipoamide) alpha 1(Pdha1)
B2GUZ6	reticulon 4 interacting protein 1(Rtn4ip1)
D3ZFR9	retinol dehydrogenase 13(Rdh13)
Q6AXX6	similar to RIKEN cDNA 5730469M10(RGD1309676)
F1LUV3	similar to glyceraldehyde-3-phosphate dehydrogenase(LOC291543)
P27867	sorbitol dehydrogenase(Sord)
Q920L2	succinate dehydrogenase complex flavoprotein subunit A(Sdha)
P97615	thioredoxin 2(Txn2)
B0K010	thioredoxin domain containing 17(Txndc17)
R9PXU4	thioredoxin reductase 1(Txnrd1)
P20788	ubiquinol-cytochrome c reductase, Rieske iron-sulfur polypeptide 1(Uqcrfs1)
P09118	urate oxidase(Uox)

Table S3 Key proteins related to fatty acid β-oxidation among differentially expressed proteins.

UniProt ID	Gene Name	Protein Description	Fold-Change*
P15651	Acads	Short-chain specific acyl-CoA dehydrogenase	2.78
P70584	Acadsb	Short/branched chain specific acyl-CoA dehydrogenase	1.44
P08503	Acadm	Medium-chain specific acyl-CoA dehydrogenase	1.76
Q5M9H2	Acadvl	Acyl-Coenzyme A dehydrogenase, very long chain	2.55
Q64428	Hadha	Trifunctional enzyme subunit alpha	1.86
Q60587	Hadhb	Trifunctional enzyme subunit beta	1.98

*represents control/emodin

Table S4 Key proteins related to mitochondrial respiratory chain among differentially expressed proteins.

	UniProt ID	Gene Name	Protein Description	Fold-Change*
Complex I	D4A565	Ndufb5	NADH:ubiquinone oxidoreductase subunit B5	2.92
	D3ZLT1	Ndufb7	NADH:ubiquinone oxidoreductase subunit B7	5.41
	B2RYS8	Ndufb8	NADH:ubiquinone oxidoreductase subunit B8	2.69
	B2RYW3	Ndufb9	NADH:ubiquinone oxidoreductase subunit B9	2.63
	D3ZS58	Ndufa2	NADH:ubiquinone oxidoreductase subunit A2	2.46
	B2RZD6	Ndufa4	NADH:ubiquinone oxidoreductase subunit A4	2.85
	Q63362	Ndufa5	NADH:ubiquinone oxidoreductase subunit A5	3.12
	D4A3V2	Ndufa6	NADH:ubiquinone oxidoreductase subunit A6	5.28
	Q7TP78	Ndufa8	NADH:ubiquinone oxidoreductase subunit A8	2.19
	Q5BK63	Ndufa9	NADH:ubiquinone oxidoreductase subunit A9	1.83

	Q561S0	Ndufa10l1	NADH dehydrogenase (ubiquinone) 1 alpha subcomplex 10-like 1	2.39
	F1LXA0	Ndufa12	NADH:ubiquinone oxidoreductase subunit A12	1.95
	O08776-4	Ndufaf3	NADH dehydrogenase [ubiquinone] 1 alpha subcomplex assembly factor 3	3.99
	D3ZE15	LOC100911483	NADH dehydrogenase [ubiquinone] 1 alpha subcomplex subunit 13-like	3.34
	Q6PCU8	Ndufv3	NADH:ubiquinone oxidoreductase subunit V3	4.22
	Q66HF1	Ndufs1	NADH dehydrogenase (ubiquinone) Fe-S protein 1	1.66
	Q641Y2	Ndufs2	NADH dehydrogenase (ubiquinone) Fe-S protein 2	2.21
	D3ZG43	Ndufs3	NADH dehydrogenase (ubiquinone) Fe-S protein 3	2.31
	Q5XIF3	Ndufs4	NADH dehydrogenase [ubiquinone] iron-sulfur protein 4	3.43
	B5DEL8	Ndufs5	NADH dehydrogenase (Ubiquinone) Fe-S protein 5	9.23
	B0BNE6	Ndufs8	NADH dehydrogenase (Ubiquinone) Fe-S protein 8 (Predicted), isoform CRA_a	2.92
complex II	P21913	Sdhb	Succinate dehydrogenase [ubiquinone] iron-sulfur subunit	2.05
	Q920L2	Sdha	Succinate dehydrogenase [ubiquinone] flavoprotein subunit	1.81
complex III	Q5M9I5	Uqcrh	Ubiquinol-cytochrome c reductase hinge protein	4.22
	B2RYS2	Uqcrb	Ubiquinol-cytochrome c reductase binding protein	3.92
	P20788	Uqcrfs1	Ubiquinol-cytochrome c reductase, Rieske iron-sulfur polypeptide 1	2.72
	P32551	Uqcrc2	Ubiquinol cytochrome c reductase core protein 2	2.38
	Q7TQ16	Uqcrq	Ubiquinol-cytochrome c reductase, complex III subunit VII	1.95
	A9UMV7	Uqcr11	Ubiquinol-cytochrome c reductase, complex III subunit XI	2.62
complex IV	P11240	Cox5a	Cytochrome c oxidase subunit 5A	4.72
	D3ZD09	Cox6b1	Cytochrome c oxidase subunit 6B1	4.05
	P10888	Cox4i1	Cytochrome c oxidase subunit 4 isoform 1	2.86
	Q8SEZ5	Mt-co2	Cytochrome c oxidase subunit 2	2.81
complex V	Q06645	Atp5g1	ATP synthase F(0) complex subunit C1G1	6.04
	P29419	Atp5i	ATP synthase subunit e	4.23
	P31399	Atp5h	ATP synthase subunit d	3.24
	Q6PDU7	Atp5l	ATP synthase subunit g	2.89
	Q06647	Atp5o	ATP synthase subunit O	2.63
	P19511	Atp5f1	ATP synthase F(0) complex subunit B1	2.43
	P35435	Atp5c1	ATP synthase subunit gamma	1.90
	D3ZAF6	Atp5j2	ATP synthase subunit f	1.83

* represents control/emodin

Table S5 Key proteins related to TCA cycle among differentially expressed proteins.

UniProt ID	Gene Name	Protein Description	Fold-Change*
G3V936	Cs	Citrate synthase	2.14
F1LNF7	Idh3a	Isocitrate dehydrogenase [NAD] subunit alpha	2.91
P41565	Idh3g	Isocitrate dehydrogenase [NAD] subunit gamma 1	2.6
P21913	Sdhb	Succinate dehydrogenase [ubiquinone] iron-sulfur subunit	2.05
Q920L2	Sdha	Succinate dehydrogenase [ubiquinone] flavoprotein subunit	1.81

* represents control/emodin

Table S6. Key proteins related to redox regulatory among differentially expressed proteins.

UniProt ID	Gene Name	Protein Description	Fold-Change*
P97615	Txn2	Thioredoxin	14.31
B0K010	Txndc17	Thioredoxin domain-containing 17	2.61
R9PXU4	Txnrd1	Thioredoxin reductase 1	2.08
Q9Z0J5-2	Txnrd2	Thioredoxin reductase 2	1.81
Q9ESH6	Glrx	Glutaredoxin-1	2.47
D4ADD7	Glrx5	Glutaredoxin 5	3.61
P35704	Prdx2	Peroxiredoxin-2	1.86
G3V7I0	Prdx3	Peroxiredoxin 3	2.69
Q9Z0V5	Prdx4	Peroxiredoxin-4	2.42
P23764	Gpx3	Glutathione peroxidase 3	2.22
P04041	Gpx1	Glutathione peroxidase 1	2.01

* represents control/emodin

Table S7 Scoring table of molecular docking result.

Protein name	PDB ID	Ligand	Total Score	Crash	Polar
Acads	2VIG	Emodin	4.8051	-0.7668	3.2436
		COS	10.7553	-3.8062	8.52
Acadsb	2JIF	Emodin	6.2675	-2.4267	2.8973
		FAD	19.7277	-3.6705	11.7201
Acadm	2A1T	Emodin	7.1097	-3.4979	5.8098
		FAD	20.2133	-4.2482	12.4602
Acadvl	3B96	Emodin	7.6312	-1.583435	3.5051
		FAD	12.655	-3.0127	10.1526
Idh3a	6KDY	Emodin	4.6943	-0.7035	6.3764
		NAD	12.7655	-1.114	9.2587
Idgh3g	5YVT	Emodin	4.7041	-0.2437	5.3685
		NAI	10.5381	-1.9585	9.8481
Complex IV	5Z62	Emodin	8.0158	-0.5664	5.5888
		HEA	14.7109	-7.8227	5.4073
Glrx	4RQR	Emodin	2.9614	-0.5462	3.469
		COM	1.5058	-0.5763	0.9257
Glrx5	2WUL	Emodin	5.598	-3.7086	6.034
		GSH	11.5536	-2.0185	12.4215
Gpx1	2F8A	Emodin	1.5046	-0.8699	3.8805
		MLA	6.2831	-0.1747	6.5363
Gpx3	2R37	Emodin	3.3498	-0.6382	5.8442
		Cl	3.3498	-0.0185	1.2274
Txnrd1	3QFA	Emodin	5.7811	-2.2288	5.2985
		FAD	21.3381	-1.7014	12.5821
Txn2	4POK	Emodin	3.0639	-0.9066	3.3881
		COM	2.5964	-0.4912	1.342
Prdx4	4RQX	Emodin	2.8898	-0.3469	2.3873
		COM	1.1513	-0.2487	1.6925
